# The impact of financing cost differences on global energy and industry decarbonization

**DOI:** 10.1038/s41558-026-02756-0

**Published:** 2026-09-24

**Authors:** Paul Waidelich, Constance Crassier, Harmen Sytze de Boer, Paul Tautorat, Detlef P. van Vuuren, Bjarne Steffen

**Affiliations:** 1 Energy and Technology Policy Group, ETH Zurich, Zurich, Switzerland; 2 NERA, Paris, France; 3 PBL Netherlands Environmental Assessment Agency, The Hague, the Netherlands; 4 Copernicus Institute of Sustainable Development, Utrecht University, Utrecht, the Netherlands

**Keywords:** Climate-change mitigation, Socioeconomic scenarios, Energy economics

## Abstract

Integrated assessment models usually neglect differences in financing conditions across countries, sectors and time. This omission could distort outcomes concerning electricity generation and also other sectors. Here we estimate and implement dynamic, country-specific cost of capital (CoC) for eight energy and industry sectors in the IMAGE integrated assessment model. Compared with assuming a uniform low-risk CoC, accounting for developing countries’ investment risks increases global mitigation costs through 2100 for staying well below +2 °C by around 9% and shifts cost-effective mitigation efforts to lower-risk regions. Sector-level analysis shows that using granular CoC estimates reduces grid investments in developing countries, shifts centralized heat production towards natural gas and raises green hydrogen costs, slowing electrolysis uptake. In industries such as steel and cement, sector and country risks hinder capital-intensive carbon capture and electricity-based technologies. Our results reveal biases from omitting country-specific financing conditions and highlight the need for climate finance across many sectors.

## Abstract (web summary)

Financing costs vary widely across countries and sectors, influencing the feasibility of global decarbonization. This research shows that accounting for these differences increases mitigation costs and shifts investment patterns, highlighting the need for broader climate finance.

## Main

As global warming rapidly approaches +1.5 °C, climate change mitigation is increasingly urgent, requiring deep decarbonization across sectors^1,2^. For decision-makers, prudent climate policy and investment decisions require a good understanding of feasible and affordable decarbonization pathways for each climate-relevant sector. Integrated assessment models (IAMs) are a key tool to assess such pathways, informing reports by the Intergovernmental Panel on Climate Change^3,4^, as well as assessments of climate targets^5^, transition risks^6^ and investment needs^7,8^. Therefore, the accuracy of how technologies and climate-relevant sectors are represented in IAMs, including relevant feasibility constraints, is central to our understanding of deep decarbonization and subject to extensive research^9–13^.

A key feasibility constraint for the low-carbon transition is the cost of capital (CoC), defined as the rate of return expected by financiers to compensate them for the risks involved^14^. A higher CoC can substantially reduce the cost competitiveness of low-carbon technologies, such as renewable energy technologies or heat pumps, which are more capital-intensive than their fossil fuel-based alternatives^15–19^. In addition, higher interest rates and return expectations make new investments less attractive, thus favouring the prolonged use of existing, typically more carbon-intensive assets^20^. Despite the CoC’s importance, IAMs—and energy system and industry decarbonization models alike^21,22^—have historically represented financing conditions through uniform CoC assumptions that abstract from risk differences between countries, sectors and technologies^21,23,24^. While such an approach was, among other things, motivated by modelling complexity, and a lack of data and best practices^21^, it may bias results considerably^25^.

Recent studies have begun to address this issue by augmenting models with more granular CoC estimates for electricity generation, highlighting important implications for developing countries in particular ^26–31^. However, the narrow scope of these studies omits CoC effects on electricity networks, the remaining energy sector and other climate-relevant sectors such as industry. Notably, electricity generation accounts for only a quarter of global greenhouse gas emissions^2^ and, compared with other climate-relevant sectors, has systematically lower market risks^32^ and abatement costs^3^. Therefore, assessing how accounting for granular sector and country risks in CoC assumptions affects sectors beyond electricity generation is crucial to evaluating the overall impact of granular financing cost estimates on mitigation pathways and costs, which remains unknown so far.

In this Article, we address this gap in the extant literature through three contributions. First, we estimate empirical, sector- and country-differentiated CoC for eight climate-relevant energy and industry sectors, such as hydrogen production, cement and steel. By doing so, we extend CoC differentiation in IAMs from electricity generation alone to other energy supply sectors, grids and emission-intensive industries. Second, we introduce an empirically calibrated feedback effect of developing countries’ economic growth on investment risks, whereas extant studies either ignore this feedback or use coarse and stylized assumptions, such as uniform long-term convergence to risk levels of high-income countries^27,30^. Lastly, by implementing our dynamic, region- and sector-specific CoC into a well-established IAM with high geographic and technological resolution, the Integrated Model to Assess the Global Environment (IMAGE) model^33^, we quantify how the CoC shapes energy and industry decarbonization and mitigation costs, including sector-specific responses, the relative contributions of sector and country risk, and the downstream effects of CoC-related energy price shifts.

## Estimating financing costs of climate-relevant sectors

**Fig. 1:**
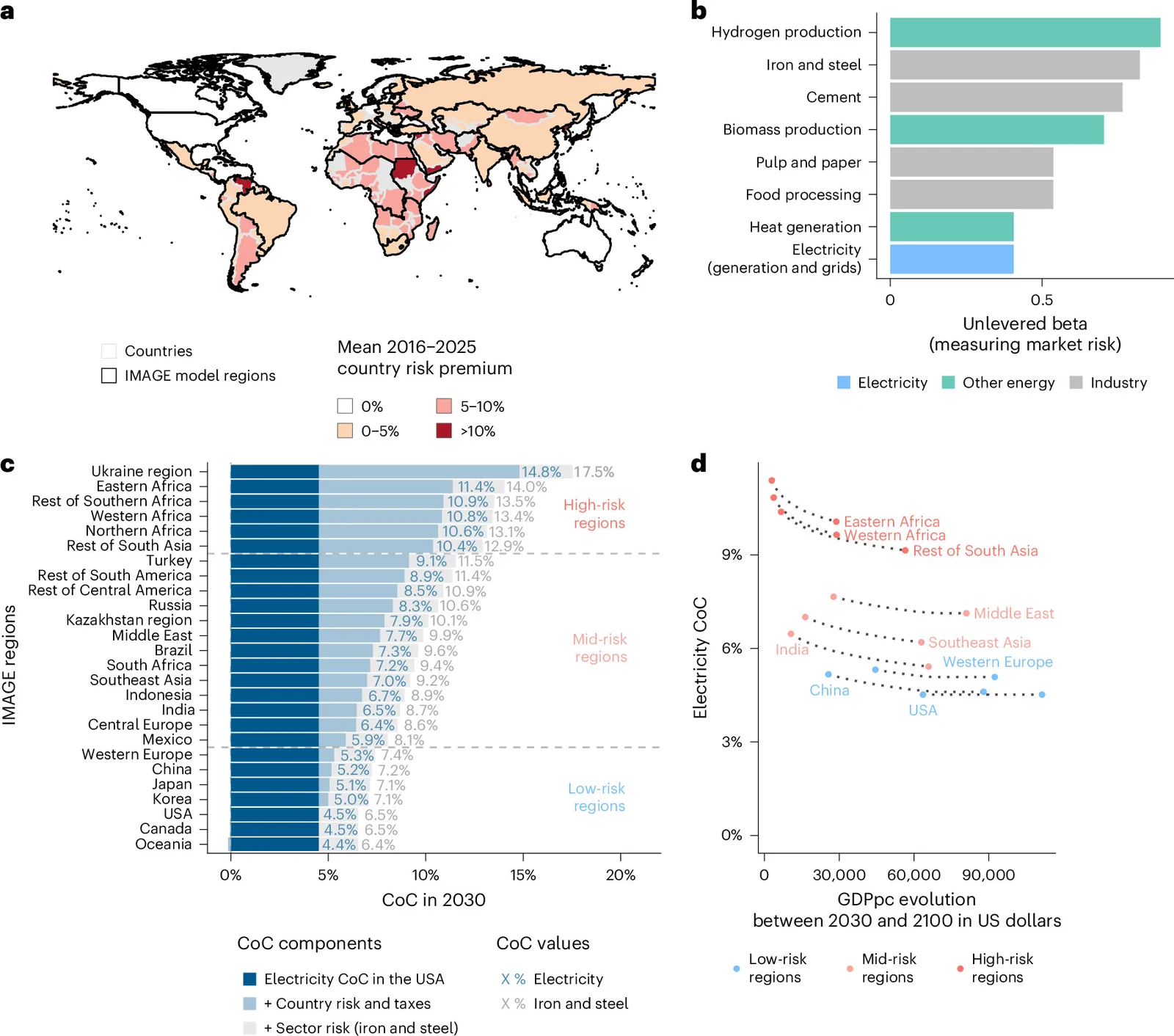
Deriving dynamic region- and sector-specific CoC estimates for the IMAGE model. **a**, Mean 2016–2025 country risk premium (start of year)^54^, with IMAGE model regions (countries) marked in black (grey). For territories coloured in grey, data are not available. **b**, Unlevered beta estimates of market risk by IMAGE sectors based on global industry-specific averages^32^. **c**, Resulting CoC estimates (nominal, after tax) for two example sectors (electricity, and iron and steel), disaggregated into the CoC without country risk for the US electricity sector (blue), CoC changes due to country risk and taxes (red; calculated as regional electricity CoC minus US electricity CoC), and additional sector risk (grey; calculated as regional iron and steel CoC minus regional electricity CoC). **d**, CoC reduction due to GDP per capita growth (SSP2) between 2030 and 2100 for an example sector (electricity), displayed for three example IMAGE model regions with the highest 2025 population per macroregion (high-, mid- and low-risk). Basemap in **a** from Natural Earth (https://www.naturalearthdata.com)^55^. Source data

While IMAGE, unlike many IAMs, explicitly represents the CoC in modelling investment decisions, its default version uses a CoC assumption uniform across regions, sectors and time^33,34^. To augment the model, we build on approaches and data sources used by recent studies that incorporate granular CoC for electricity generation in IAMs and similar models^21,26–28,30,31^. To capture risk differences, we take country risk premiums based on sovereign ratings (Fig. 1a) and sector-specific risk measures based on stock price patterns (Fig. 1b) from a widely used database^35^ and apply them to IMAGE by aggregating country variables to model regions and matching database industry groups to IMAGE’s sectors. The resulting region- and sector-specific estimates of current financing costs reveal substantial heterogeneity (Fig. 1c). For instance, the CoC of the electricity and steel sectors in the USA differ by approximately two percentage points, equivalent to the difference between electricity generation projects in the USA and those in India or Indonesia. Notably, country risk differences can be even larger, with the electricity CoC in high-risk regions, such as Eastern Africa or Ukraine, being around three times higher than in the USA. We categorize IMAGE’s model regions as high-, mid- or low-risk based on their CoC for the electricity sector (Fig. 1c, dashed lines). To capture how the CoC may evolve in the future, we estimate the empirical relationship between country risk premiums and income per capita growth, a key rating determinant^36–38^, and translate regions’ projected growth into CoC reductions (Fig. 1d; for details, see Methods).

**Fig. 2:**
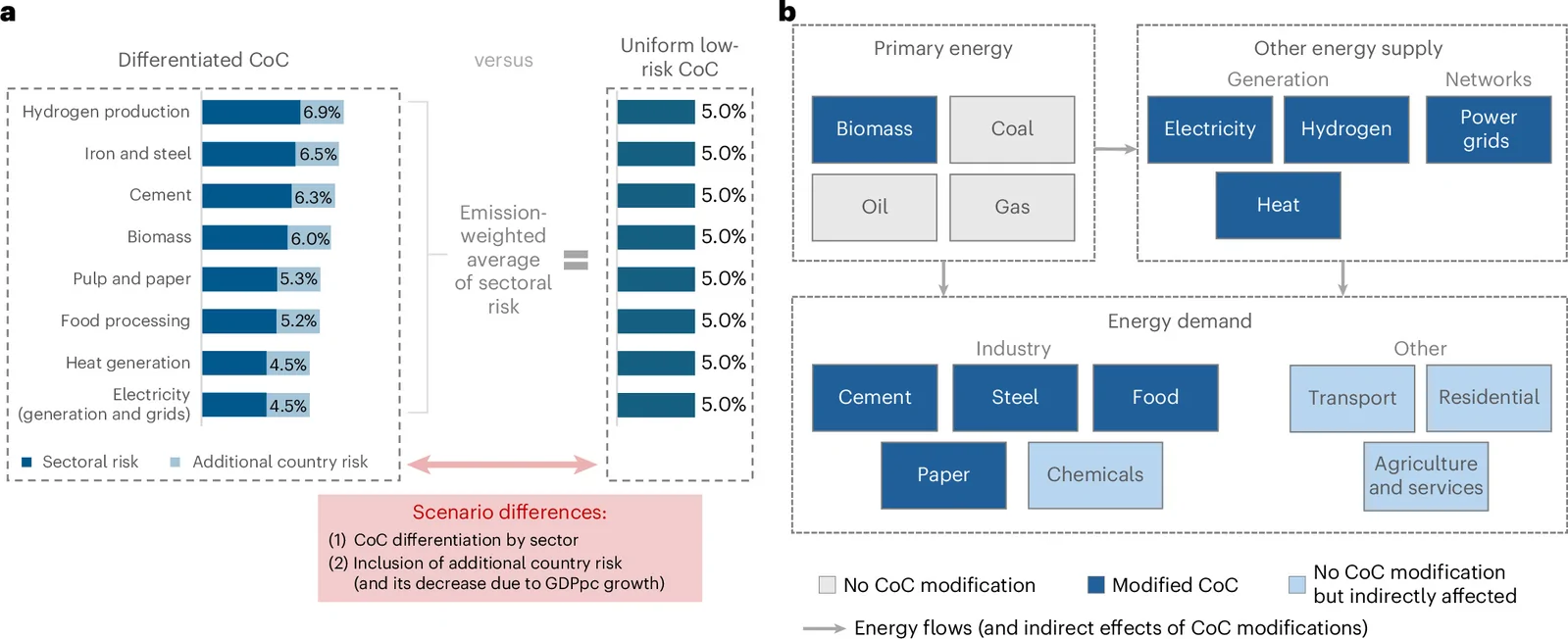
IMAGE scenario definitions and sectoral scope of the CoC modifications. **a**, Schematic representation of our differentiated CoC and uniform low-risk CoC scenarios, with value labels by sector for the USA (as a key risk-free region). For details on the emission-weighted average, see Supplementary Table 2. Differentiated CoC estimates by region and sector are provided in Supplementary Table 9. **b**, Overview of IMAGE sectors, for which we directly modify the CoC (blue), which are indirectly affected by upstream CoC changes through energy price shifts (light blue), or which are unaffected by our CoC changes (grey).

We run IMAGE in two settings to explore the impacts of differentiating the CoC by region, sector and over time, either using our differentiated CoC estimates or a uniform low-risk CoC of 5% (Fig. 2a). The latter corresponds to an emission-weighted average of our empirical estimates for the USA as a low-risk country across all sectors considered (Supplementary Table 2). Therefore, the uniform low-risk CoC averages risks across sectors while omitting country risk. This approach is numerically consistent with benchmarks from previous CoC studies^30,39^, and it emulates the common practice in energy system models and IAMs of using uniform CoC assumptions based on lower-risk markets (Supplementary Note 1). CoC assumptions in IMAGE are modified only for the sectors within our scope, but these changes can affect other sectors indirectly through energy price spillovers, such as transportation and residential heating (Fig. 2b). We explore CoC effects in a current policies pathway and a pathway with a carbon budget that ensures global warming remains well below +2 °C in line with the Paris Agreement, which result in +2.8 °C and +1.7 °C of global warming by 2100, respectively. Notably, in the well below +2 °C pathway, the model adjusts climate policy stringency (via global carbon pricing) endogenously to ensure that the climate target is met.

## CoC effects on overall emissions and mitigation costs

**Fig. 3:**
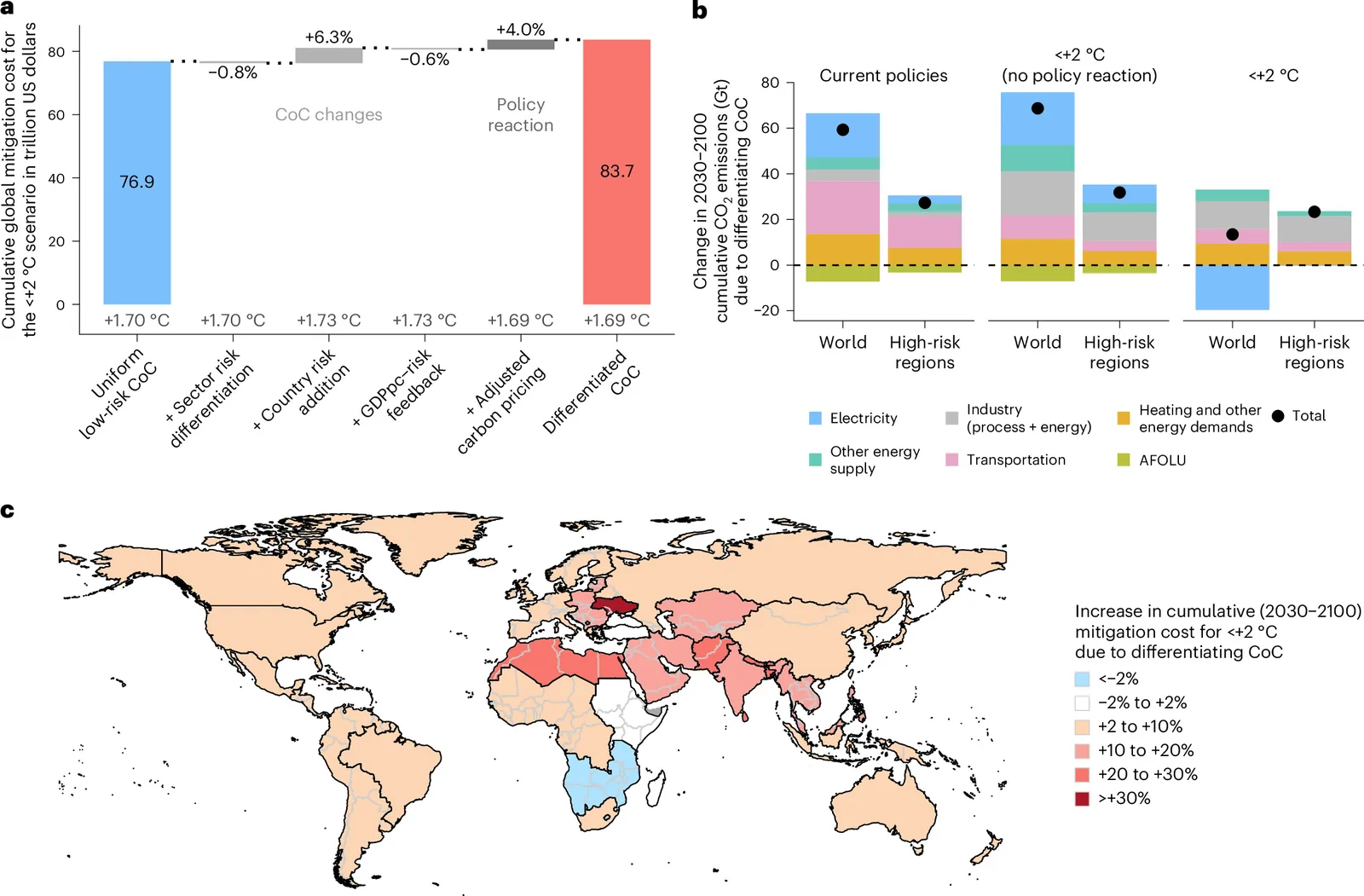
Effect of differentiated CoC on CO_2_ emissions and mitigation costs. **a**, Global cumulative mitigation cost (2030–2100) under the well below +2 °C scenario for the uniform low-risk CoC (blue) and the differentiated CoC (red) scenarios. ‘+*X* °C’ labels below the bars indicate the change in global mean surface temperature by 2100 vis-á-vis pre-industrial. Discounted mitigation costs are provided in Supplementary Fig. 8b. **b**, Change in global cumulative (2030–2100) CO_2_ emissions between the uniform low-risk CoC and the differentiated CoC scenario by sector (bars) and across all sectors (black dots) for the current policies scenario and the well below +2 °C with and without climate policy reacting to the CoC changes. AFOLU denotes agriculture, forestry and other land uses. **c**, Relative shift in the region-specific cumulative mitigation cost (2030–2100) between uniform low-risk CoC and the differentiated CoC scenario. Basemap in **c** from Natural Earth (https://www.naturalearthdata.com)^55^. Source data

Compared with the uniform low-risk CoC scenario, introducing our differentiated CoC estimates for the eight sectors in our scope increases cumulative mitigation costs by roughly 9% from 77 to 84 trillion US dollars (Fig. 3a). We decompose this increase into different components by first holding climate policy stringency constant at the levels of the uniform low-risk CoC approach and introducing our CoC differentiation elements step by step (‘CoC changes’) before finally allowing the model to adjust climate policy endogenously to comply with the climate target despite higher CoC in many countries (‘policy reaction’). Doing so reveals that sectoral risk differentiation lowers mitigation cost (primarily through lower electricity prices in some low-risk regions; see the next section), while the increase in mitigation cost is primarily driven by the addition of country risk (+6%) and only mildly mitigated by future economic growth. Notably, the higher CoC in developing countries due to their country risk makes mitigation options less financially viable and, when holding climate policy stringency constant, increases global warming through 2100. Allowing the model to increase policy stringency endogenously reduces this additional warming but increases cumulative mitigation costs considerably (+4%). Notably, the overall increase in mitigation costs is primarily driven by shifts in the electricity and heat CoC assumptions and their indirect effects via energy price spillovers (Supplementary Fig. 8).

Implementing our differentiated CoC estimates in IMAGE increases emissions (Fig. 3b). Under current policies and the well below +2 °C pathway without a climate policy reaction, global cumulative CO_2_ emissions through 2100 increase by roughly 60–70 Gt, which is equivalent to more than one additional year of current emission levels and strongly driven by high-risk regions. In part, these increases in the differentiated CoC vis-á-vis the uniform low-risk CoC results are driven by the electricity sector, in line with previous studies’ finding that the introduction of country risk shifts this sector towards less capital-intensive technologies using fossil fuels and raises power prices^15,16,18,25–28,30^. In addition, using the differentiated CoC increases current-policies emissions in other energy supply sectors, in industry, and, notably, in sectors for which we do not implement CoC estimates, such as transport and heating, due to energy price spillovers. If the model’s endogenous climate policy reaction is included, the shift in total cumulative emissions is much smaller. This is because the additional transport, heating and industry emissions in higher-risk regions when using differentiated CoC estimates, which become increasingly costly to abate, are now offset by additional abatement in the electricity sector, particularly in low- and mid-risk regions.

As a result, these additional mitigation efforts increase cumulative mitigation cost in higher-risk regions and also in most mid- and low-risk regions (Fig. 3c). The effect is most pronounced for regions where key economies have persistently poor credit ratings, such as Ukraine or South Asia excluding India. However, mitigation cost increases are also substantial for major low-risk economies, such as the USA and China (+5–6%), while in some high-risk regions in Eastern and Southern Africa, abatement efforts (in tonnes of CO_2_) decrease so much that overall abatement costs decrease.

## CoC effects on the energy sector

**Fig. 4:**
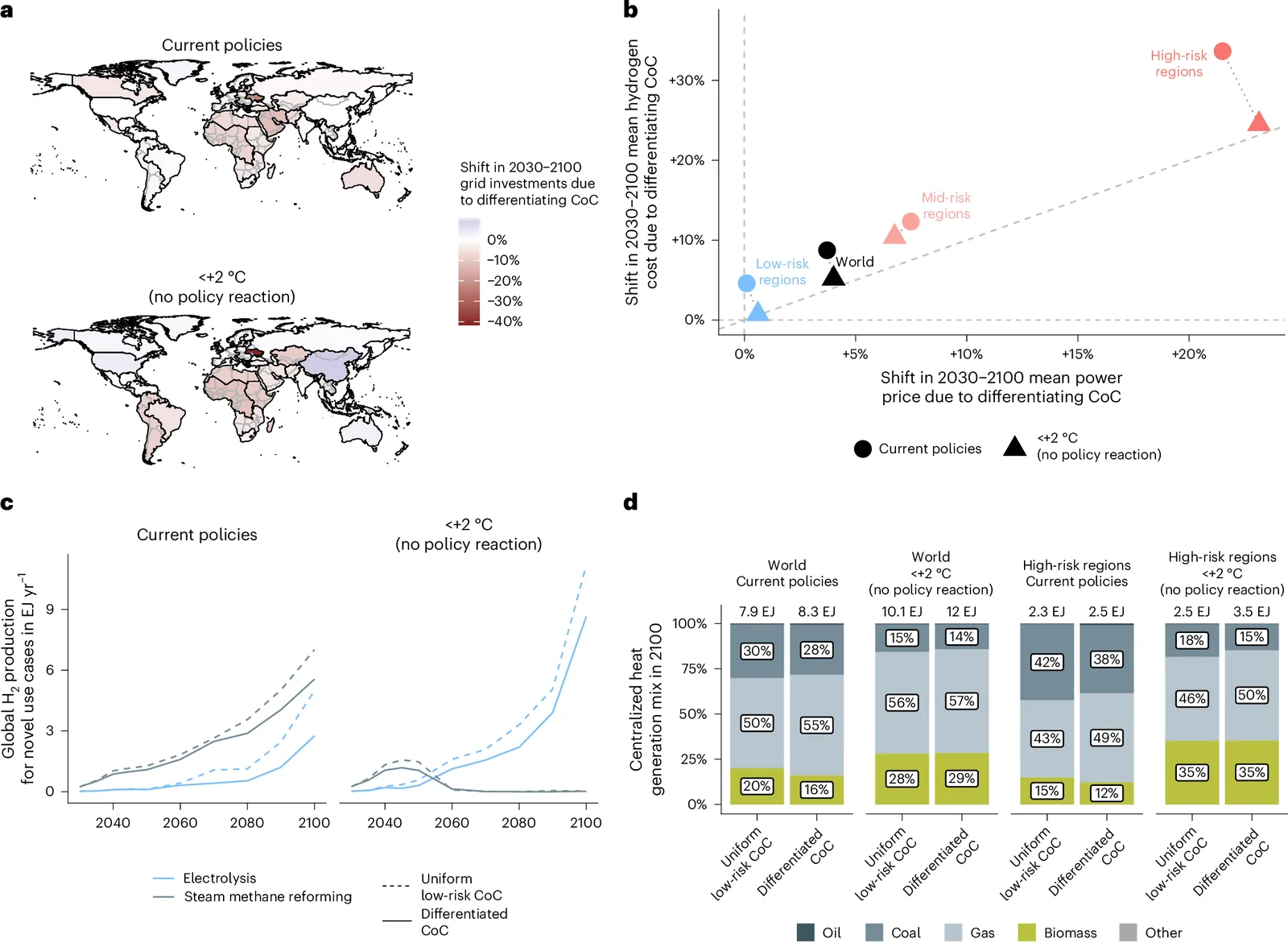
Effect of differentiating the CoC on grid investments, energy prices, and hydrogen and heat generation under the current policies and the well below +2 °C scenario without policy reaction. **a**, Shift in the cumulative investments in electricity grids (2030–2100) between uniform low-risk CoC and the differentiated CoC. For details on how grid investments are modelled in IMAGE, see Supplementary Note 5. **b**, Shift in the mean 2030–2100 hydrogen (*y* axis) and electricity (*x* axis) prices between uniform low-risk CoC and the differentiated CoC. For details on hydrogen cost in IMAGE, see Supplementary Note 5. **c**, Global hydrogen production for novel use cases by technology (colour) and CoC setting (linetype). ‘Steam methane reforming’ includes plants with and without CCS. ‘Electrolysis’ comprises proton-exchange membrane, solid oxide and alkaline electrolysers. Other IMAGE technologies (that is, partial oxidation of oil and gasification of biofuels and coal) are omitted because of negligible production volumes. Hydrogen production for legacy use cases (for example, ammonia and refineries) is not included here (Supplementary Note 5). **d**, Centralized heat generation mix in 2100 worldwide and for the high-risk macroregion. ‘Other’ comprises geothermal and hydrogen. Labels above each bar denote the total heat generation. Basemaps in **a** from Natural Earth (https://www.naturalearthdata.com)^55^. Source data

When breaking down these main findings by sector, we generally omit the climate policy reaction to isolate the pure CoC effect on sectoral outcomes. For electricity generation, our results largely corroborate the extant literature’s findings. Introducing country risk reduces the share of renewables and nuclear in high-risk regions’ long-term electricity mix to the benefit of natural gas (Supplementary Figs. 19 and 20)—which is less capital-intensive and, hence, less sensitive to CoC increases than coal^18^. Moving beyond previous studies, we find that differentiating the CoC reduces developing countries’ grid investments both in the current policies and the well below +2 °C pathway (Fig. 4a). This finding is highly relevant given that the grid sector has similarly high or even higher net-zero investment needs than electricity generation^40^.

Some of these grid investment reductions reflect a lower electricity demand, as the higher CoC for electricity generation and networks in mid- and high-risk regions increases the average electricity price between 2030 and 2100 (Fig. 4b). Hydrogen cost increases due to the differentiated CoC overall are even more pronounced than for electricity and affect nearly every region, with increases well above 20% for high-risk regions such as Western Africa that are often envisaged as potential hydrogen exporters^41^.

The strong hydrogen cost effect arises primarily because hydrogen production is currently dominated by companies associated with the chemical sector, which exhibits a relatively high sector risk (Fig. 1b). In addition, in the case of electrolysis, hydrogen production is fully exposed to CoC-induced shifts in electricity prices—although this spillover effect plays a more minor role in IMAGE (Supplementary Figs. 10 and 11). For these reasons, global hydrogen production using electrolysis drops substantially when differentiating the CoC (Fig. 4c). By contrast, the decrease in the current incumbent technology, natural gas-based steam methane reforming, is less pronounced—except in low-risk regions where differentiating the CoC increases power prices only modestly or decreases them (Supplementary Fig. 21). As hydrogen, on average, becomes more CO_2_-intensive and costly, it becomes less attractive for decarbonizing other sectors, such as transport, industry and electricity generation, whose global end-of-century hydrogen usage under the well below +2 °C scenario drops accordingly (Supplementary Fig. 22).

Differentiating the CoC also shifts centralized heat production in the model (that is, district heating and combined heat and power generation) away from biomass and coal toward natural gas, particularly in high-risk regions under the current policies pathway (Fig. 4d). While this fuel shift and the higher CoC translate into higher heat prices, the increase is less pronounced than for electricity (Supplementary Fig. 23). As a result, the (modest) global volume of centralized heat production by 2100 under the well below +2 °C scenario increases as purchasing heat becomes less expensive for companies and households relative to on-site generation using heat pumps.

## CoC effects on industry and fuel mixes

**Fig. 5:**
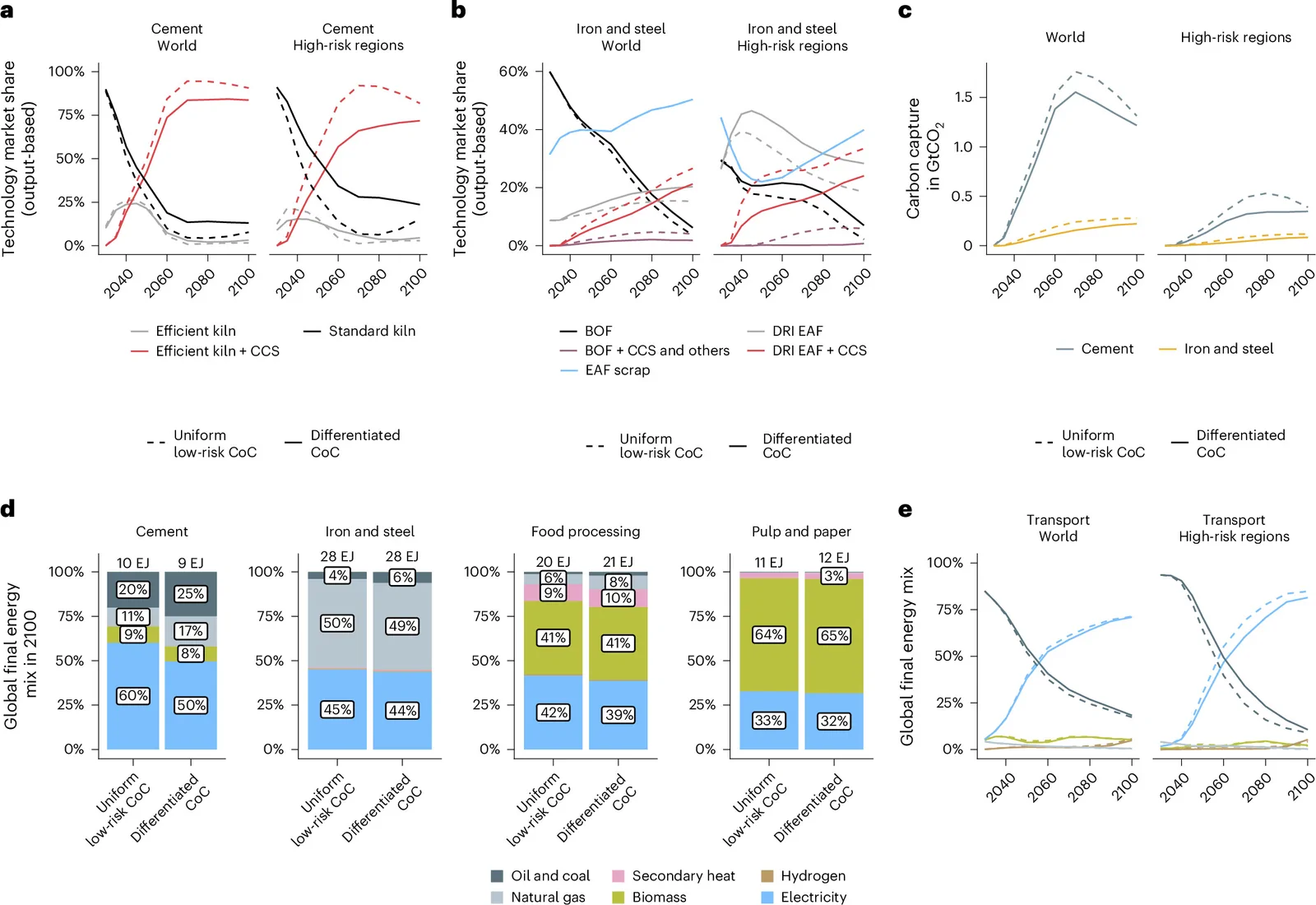
Effects of differentiating the CoC on industry technology and fuel mixes for the well below +2 °C scenario without climate policy reaction. **a**, Technology share in cement production (measured in tonnes of cement) worldwide and for the high-risk macroregion. ‘Efficient kiln + CCS’ comprises precombustion oxy CCS, postcombustion monoethanolamine CCS and on-site direct separation CCS. **b**, The same as **a** but for the iron and steel sector. ‘BOF + CCS & Others’ comprises BF/BOF with CCS, H-DRI/EAF and electrowinning/EAF. Note that EAF scrap production in IMAGE is primarily constrained by scrap availability and, therefore, does not vary with the CoC in a decarbonization scenario^56,57^. **c**, Carbon capture by the cement and steel sector (process and energy emissions) worldwide and for the high-risk macroregion. **d**, Fuel mix of different industry sectors. Labels above each bar denote the total final energy demand. **e**, The same as **d** but for the transport sector (comprising domestic and international passenger and freight transport via road, air and ship) and displayed over time. Source data

When exploring the sectoral effects of differentiated CoC estimates for the four climate-relevant industry sectors covered by our CoC estimates (that is, cement, iron and steel, pulp and paper, and food processing), we focus on the well below +2 °C scenario because, unlike for electricity generation, industry decarbonization efforts in the current policies scenario are limited (Supplementary Fig. 24) owing to the more challenging underlying economics^3,42^. For the cement sector, which accounts for about 7% of current global CO_2_ emissions^43^, using the differentiated CoC substantially alters the sector’s technology mix (Fig. 5a). Under the well below +2 °C scenario, the sector rapidly switches from the incumbent standard kiln technology to more efficient new-build kilns using the best available technology in IMAGE, typically augmented with carbon capture and storage (CCS). However, country and sector risks limit and slow down this transition. As a result, the global market share of inefficient standard kilns without CCS by 2100 increases compared with the uniform low-risk CoC setting.

Our findings for the iron and steel sector, which accounts for roughly 8% of current global CO_2_ emissions^44^, corroborate these patterns (Fig. 5b). Overall, the sector’s higher CoC translates into higher market shares of the main incumbent technology, which reduces iron ore in coke-fuelled blast furnaces before refining it to crude steel in basic oxygen furnaces (BOF). Importantly, BOF is considerably more carbon-intensive than the alternative electric arc furnaces (EAF), which produce crude steel either from recycled scrap or direct-reduced iron (DRI) using natural gas for the reduction process^43^. Differentiating the CoC reduces the uptake of CCS for both DRI EAF and BOF, leading to a higher market share of unabated technology versions, and further reduces the small market share for novel, less carbon-intensive technologies such as electrowinning/EAF or direct reduction using hydrogen. Overall, the amount of industrial CCS by the cement and steel sectors decreases substantially, which is overwhelmingly driven by high-risk regions (Fig. 5c) and sectoral CoC rather than CoC-induced shifts in energy prices (Supplementary Figs. 12 and 13). The key reason for these patterns is that newer cement production technologies and, in particular, CCS deployment increase capital intensity^45^, making costs more sensitive to CoC changes.

Beyond technology choices and CCS deployment, differentiating the CoC also affects the fuel mix in climate-relevant industries and, through energy price spillovers, in other non-energy sectors, with a substantial effect on transportation emissions in particular (Fig. 3b). In cement and steel, higher power prices due to country risk reduce electricity usage, which is primarily replaced by fossil fuels (Fig. 5d). Food processing and pulp and paper, the two other industrial sectors with explicit CoC representation in IMAGE, also exhibit a shift from electricity to biomass usage and, for food processing, fossil fuels. Beyond the industrial sector, CoC-induced power price increases also delay the transportation sector’s switch from oil-based fuels to electricity in high-risk regions (Fig. 5e), particularly in passenger vehicles and trucking.

## Discussion

We show that not capturing CoC differences across countries biases mitigation cost estimates, while the effect of sectoral risk differences is less pronounced. This result is partially driven by CoC-related cost shifts in electricity and clean hydrogen and their spillover effects on other sectors. In the energy sector, omitting country and sector risk differences may result in too optimistic assessments of grid expansion, electrification and the potential of green hydrogen and its derivatives (for example, synthetic aviation fuels) for decarbonization. In industry, above-average sector risks, potentially amplified by country risk, reduce the economic viability of technology upgrades and industrial CCS. These overall conclusions remain robust when using alternative sector risk estimates or a stronger income–country risk feedback (Supplementary Notes 1, 3 and 4), while relying on an alternative database yields similar CoC estimates and risk rankings (Supplementary Note 2). Importantly, for IAMs that use high uniform CoC assumptions of 8–10%, often stemming from previous decades with higher interest rates, the sign of the reported effects may be reversed (as empirical CoC estimates are below model assumptions for many regions and sectors), but the overall insights on how the CoC shapes model outcomes remain unchanged (Supplementary Note 4).

Therefore, modelling studies using uniform CoC assumptions should revise their CoC assumptions, particularly by differentiating country risk. To this end, modellers can leverage the values and methodologies presented here, which can be readily applied to other sectors and models with varying geographical resolution. Our results can also guide modellers on which sectors may exhibit sizable CoC effects (for example, hydrogen or industrial CCS) and for which sectors effects may be less pronounced. For lower-risk regions in particular, CoC differentiation may affect model results and performance less than other model inputs, such as technology availability^46^.

Our results also indicate that CoC-driven technology and fuel shifts in other climate-relevant sectors, including industry, have a similar or even higher magnitude than for electricity generation. This novel finding is driven by these sectors’ above-average risk and the higher capital intensity of novel industrial technologies, particularly those involving CCS^45^.

Regarding policy implications, our results suggest that the economic growth of developing countries, although important, is probably insufficient to mitigate the current CoC differences between countries fully—unless accompanied by improvements in other sovereign risk determinants that do not scale with economic growth^36^. At the same time, we show that country risk in particular hinders mitigation across sectors and increases costs, which underscores the strong need for improving sovereign risk determinants through institutional, regulatory and debt sustainability improvements. Well-designed international climate finance can help to derisk investments across all climate-relevant sectors in developing countries to avoid potential investment traps^30^, but international mitigation finance flows remain focused on low-carbon electricity generation^47^.

The findings presented in this study are accompanied by some caveats. While we expand the scope of differentiated CoC vis-á-vis previous, electricity generation-focused studies, we model CoC-induced energy price effects but not explicit CoC impacts on the transport, agriculture and some industrial sectors, most notably chemicals. This limitation reflects the less explicit CoC representation in IMAGE for these sectors^33^ and, for transportation, the sector-specific role of consumer finance, income segment-specific financing conditions^48^ and leasing. Furthermore, we estimate the CoC on the basis of recent history and, for future years, consider changes introduced by economic growth but hold sectoral risks constant. Doing so abstracts from sectors’ potential trends—for instance, if steel decarbonization reshuffles supply chain structures that alter the industry’s overall risk^49^. In addition, because IMAGE treats GDP growth as exogenous, it omits incentives for policymakers to lower future CoC by stimulating growth. This highlights how structural differences across IAMs could introduce further nuances to our findings. Given the volatility of region-specific estimates^50^, our estimates of current sectoral risks are based on global averages, which may underestimate sectoral risks in developing countries. Lastly, our CoC estimates capture market conditions and rates. In higher-risk developing countries, these market rates are somewhat mitigated by existing international climate finance^26^ and other policy interventions that are typically not represented in IAMs.

Future research can address these shortcomings by expanding the scope of CoC differentiation to sectors not included here, most notably chemicals and transportation. Model intercomparison studies leveraging multiple IAMs could corroborate and further contextualize the numerical findings presented here, for instance, by exploring how different structural assumptions on foresight, GDP growth endogeneity, availability of scalable carbon capture technologies and intertemporal optimization may alter CoC effects on low-emission pathways. Scholars could also translate expected shifts in sectoral risks (including stranding and physical climate change risks^51,52^), institutional quality and international climate finance into existing CoC estimates. In addition, future studies should incorporate detailed representations of policy interventions targeting the CoC^53^ to examine the effects on decarbonization outcomes and costs across sectors.

## Methods

### The IMAGE model

IMAGE is a comprehensive IAM that simulates the environmental impacts of human activities worldwide, covering topics such as climate change, biodiversity and resource use. The framework links socioeconomic developments with changes in the Earth system, enabling scenario analysis for sustainability and policy assessment. IMAGE covers 26 regions from 1970 through 2100 in yearly time steps; however, in line with the harmonized IAM reporting framework, we use output data in 5-year (10-year) intervals through (after) 2050 and, where necessary, interpolate linearly. The core model takes socioeconomic drivers from the Shared Socioeconomic Pathways (SSPs) and translates them into agriculture and land use projections using a land use model and energy supply, conversion and demand projections using the TIMER energy system model. TIMER simulates year-to-year investment decisions based on bottom-up engineering information and defined investment rules, rather than optimizing for perfect foresight. Its submodels cover energy demand, energy conversion, fossil and non-fossil fuel supply, energy trade and emissions. Key mechanisms include price-driven fuel and technology substitution, cost reductions through learning-by-doing, resource depletion modelled with long-term supply curves, and explicit simulation of trade barriers across regions. Overall, different technologies and energy carriers compete based on their costs and are chosen via a multinomial logit function. Costs include operating expenses and capital expenses, with the latter being transformed into an annuity based on asset lifetime and the CoC. Notably, IMAGE does not include a bottom-up representation of all energy and industrial sectors, such that CoC effects for a few climate-relevant (sub)sectors (for example, chemicals and glass) cannot be explored without overhauling the current model structure. As a result, CoC effects for these sectors fall outside the scope of this Article. Throughout the Article, we use SSP2 for all model runs and all monetary model results are given in 2010 US dollars adjusted for purchasing power parity (PPP) unless specified otherwise. For more details on the model, we refer to Supplementary Note 5 and the model documentation^33^.

Mitigation costs in IMAGE are calculated as the area under dynamic region- and greenhouse-gas-specific marginal abatement curves and represent additional system costs that arise from reducing greenhouse gases beyond the trends already included in the baseline scenario^33^ (for more details, see Supplementary Note 5). We present undiscounted cumulative costs to avoid potentially contested assumptions about the social discount rate^58^ but ensure that our findings are robust to using IMAGE’s default social discount rate (Supplementary Fig. 8b), which uses Ramsey discounting with a rate of pure time preference of 0.1% and an elasticity of marginal utility of consumption of one applied to regional GDPpc growth. Importantly, the CoC estimates used in this study represent private discount rates based on investors’ return expectations to ensure that technology costs reflect actual financing costs. They should not be used as normative social discount rates to discount societal costs and benefits as a whole.

For visual conciseness, we aggregate the 26 regions of IMAGE into three macroregions (Supplementary Fig. 17) based on the electricity CoC, with implied thresholds at 5.5% (which effectively separates non-Latin American Organisation for Economic Co-operation and Development (OECD) countries and China from the rest of the world) and 10% (which separates emerging markets from high-risk regions, including African countries except South Africa and model regions that include only few countries with persistently poor sovereign ratings, such as the Ukraine region).

### Scenarios for climate policy ambition and CoC

We use two different scenarios capturing a range of different climate policy ambitions, which in IMAGE is primarily represented via carbon pricing: (1) the current policies scenario in which there is no carbon price beyond current policies, which are extended until the end of the simulation^59^; (2) the well below +2 °C mitigation run, in which the model solves the optimization problem of reaching respective carbon budgets (1,000 GtCO_2_ between 2020 and 2100^60^) at the lowest achievable policy costs by setting a global carbon tax. Each of these scenarios is paired with two different scenarios for how the CoC in IMAGE is implemented. Under the uniform low-risk CoC scenario, all regions and technologies use a static, uniform 5% value, which approximately equals the average across the empirical estimates for our sectors if we weigh each sector with their 2025 global CO_2_ emissions in IMAGE under the current policies scenario (Supplementary Table 2). Under the differentiated CoC scenario, we implement dynamic region- and sector-specific CoC (for details, see below), which (1) adds country risk for all countries not rated at risk-free levels, (2) adds sector risk to most sectors (but reduces the CoC for the lowest-risk sectors, such as electricity and heat) and (3) accounts for the potential reducing effect of continued economic growth on countries’ risk premiums. As IMAGE is calibrated to historical data for past years, we modify the model’s CoC assumptions from 2030 onwards. Under the well below +2 °C scenario, the model endogenously revises the carbon pricing to ensure that the carbon budget is not violated (‘with climate policy reaction’). To isolate the CoC effect, we rerun the well below +2 °C pathway with our differentiated CoC estimates while holding the carbon price schedule of the uniform low-risk CoC run constant (‘no climate policy reaction’) and use these results when exploring sector-specific CoC effects (Figs. 4 and 5).

To distinguish direct CoC effects from indirect effects via CoC-induced energy price spillovers, we also run a differentiated CoC (power + heat only) scenario, in which we implement our region- and sector-specific CoC estimates only for electricity (generation and grids) and heat generation. Results are presented in Supplementary Note 4.

Notably, the oil and gas sector is not included within the scope of our analysis because fossil fuel prices in IMAGE are not driven by CoC assumptions and instead are based on supply curves informed by expert forecasts and real-world prices. Similarly, the chemicals sector is excluded from our industry analysis because IMAGE currently has no sector-specific investment representation that accounts for the CoC (for details, see ref. ^33^).

### Weighted average CoC

To derive country- and sector-specific CoC estimates, we use financial market data and assessments by credit rating agencies to quantify overall average risk profiles of the different climate-relevant sectors represented in IMAGE. We rely on the NYU Damodaran database^35^, which provides rating-based country risk premiums and industry-specific financial variables (for example, betas or equity shares) with annual updates and is widely used in the extant literature^27,28,30,61^. More information on approaches and concepts used in the database are provided in refs. ^50,62^. Robustness checks using the proprietary Kroll Cost of Capital Navigator database^63^ are provided in Supplementary Note 2. To estimate financing costs, we calculate the nominal weighted average CoC (WACC) after taxes for country *i* and sector *a* as follows:

1 $$\begin{array}{l}{{\rm{W}}{\rm{A}}{\rm{C}}{\rm{C}}}_{i,a}=\mathop{\underbrace{(1-{{\rm{D}}{\rm{S}}}_{a})}}\limits_{{\rm{E}}{\rm{q}}{\rm{u}}{\rm{i}}{\rm{t}}{\rm{y}}\,{\rm{s}}{\rm{h}}{\rm{a}}{\rm{r}}{\rm{e}}}\times \mathop{\underbrace{({\rm{R}}{\rm{F}}+{{\rm{C}}{\rm{R}}{\rm{P}}}_{i}^{E}+{\beta }_{a}\times {\rm{E}}{\rm{R}}{\rm{P}})}}\limits_{{\rm{C}}{\rm{o}}{\rm{s}}{\rm{t}}\,{\rm{o}}{\rm{f}}\,{\rm{e}}{\rm{q}}{\rm{u}}{\rm{i}}{\rm{t}}{\rm{y}}}\\\qquad\qquad\quad+\mathop{\underbrace{{{\rm{D}}{\rm{S}}}_{a}}}\limits_{{\rm{D}}{\rm{e}}{\rm{b}}{\rm{t}}\,{\rm{s}}{\rm{h}}{\rm{a}}{\rm{r}}{\rm{e}}}\times \mathop{\underbrace{({\rm{R}}{\rm{F}}+{{\rm{C}}{\rm{R}}{\rm{P}}}_{i}^{D}+{{\rm{D}}{\rm{P}}}_{a})}}\limits_{{\rm{C}}{\rm{o}}{\rm{s}}{\rm{t}}\,\mathrm{of}\,{\rm{d}}{\rm{e}}\mathrm{bt}\,(\mathrm{pre-tax})}\times \mathop{\underbrace{(1-{\tau }_{i})}}\limits_{{\rm{T}}{\rm{a}}{\rm{x}}\,{\rm{s}}{\rm{h}}{\rm{i}}{\rm{e}}{\rm{l}}{\rm{d}}}.\end{array}$$

Here, DS denotes the sector-specific debt share in the capital structure, while 1 − DS is the share of equity. Industry-specific debt shares are taken from the NYU Damodaran database as global average values and calculated as the ratio of firms’ total debt used (including lease debt) to the sum of total debt and market capitalization.

The cost of equity has the following components:

- Risk-free rate (RF): We use the 2015–2024 arithmetic average of US 10-year Treasury bond yields provided by the OECD^64^, RF = 2.48%. Notably, this value is considerably lower than Treasury bond yields observed in the first half of 2025, which exceed 4%. Therefore, an implicit assumption is that, in the medium to long run, interest rate levels revert to values that are more representative of the past decade.
- Country risk premiums (CRP): Year- and country-specific country risk premiums (relative to a country considered risk-free) are derived from sovereign credit ratings, which typically operate as a ‘sovereign ceiling’ on domestic firms’ credit rating^65^. Local currency ratings (as of 1 January) and the corresponding risk premiums, following the methodology outlined in ref. ^66^, are obtained from ref. ^54^. For non-rated countries, the data source imputes the country risk premium based on a country’s composite risk score by Political Risk Services. To reflect that equity is inherently riskier than debt, the country risk premium for equity (CRP^*E*^) features a multiplier of approximately 1.35, which ref. ^50^ estimates on the basis of the relative volatility of stock and bond returns in emerging markets (where country risk is most prevalent), such that CRP^*E*^ = 1.35 × CRP^*D*^. Specifically, it is obtained by calculating the standard deviation of the S&P BMI Emerging Broad Market Index and the iShares JP Morgan Emerging Markets Bond ETF for each year from 2020 to 2024 and averaging the resulting ratio across years. For the rationale behind this adjustment and further methodological details, we refer readers to ref. ^50^. To avoid individual years dominating the assumed value, we take country risk premium estimates from the NYU Damodaran database for 2016–2025 (January updates of each year) and average them.
- Equity risk premium (ERP): We use a global value of ERP = 5%, in line with the long-term global estimate by ref. ^67^ and consistent with survey estimates and premiums implied by stock market data listed in table 27 of ref. ^50^. Notably, this approach is not country-specific because, while a uniform ERP does not reflect equity risk differences between markets, confidence intervals around market-specific ERP estimates are usually so large that they severely limit their usability, particularly for less mature stock markets^50^.
- Levered industry betas (*β*): Unlevered industry-specific betas, which do not reflect risks due to indebtedness, are taken from ref. ^32^ as global averages estimated based on 5 years of stock return data. A 5-year ‘look-back period’ is standard practice for estimating betas^68^, and using longer-term averages of industry betas does not affect our conclusions (Supplementary Note 4). Betas are relevered at the country level using ${\beta }_{i,a}={\beta }_{a,\mathrm{unlevered}}\times \left[1+(1-{\tau }_{i})\times \frac{{\mathrm{DS}}_{a}}{1-{\mathrm{DS}}_{a}}\right]$ where *τ*_*i*_ denotes the country-specific corporate tax rate (more details below). Because betas are multiplied by the ERP, *β* > 1 denotes industries that are riskier than the overall market, while *β* < 1 denotes less risky industries. We use global beta estimates because, similar to the ERP, country- or region-specific betas are subject to large uncertainties and can lead to underestimated risk measures for emerging markets with low weights in global indices^50^. Doing so means that the estimated CoC differences between low- and high-risk regions arise solely from differences in country risk premiums and taxes, making this a conservative assumption.

In addition, the cost of debt has the following components:

- Debt premium (DP): Debt premiums reflect the average markups on debt that industries pay compared with the risk-free rate and are estimated based on the volatility of stocks in a given industry (as a simplified measure of industry risk) and taken from ref. ^69^. Values range between 1.54% and 1.99% for the sectors considered here. More details on this approach, as well as a robustness check using alternative approaches in line with previous literature^27,30,62^, are discussed in Supplementary Note 1.
- Corporate tax rates (*τ*): Country-specific corporate tax rates for 2025 are sourced from ref. ^70^ and, for countries with missing entries, from an international database compiled by PwC^71^ to account for the ‘tax shield’ because debt repayments are tax-deductible, which reduces the effective cost of debt financing accordingly.

Importantly, by using market rates for country and sector risk, our estimates reflect the financing costs that projects and companies actually face, without taking a stance on whether financial markets are pricing these risks correctly.

Lastly, in electricity generation, renewable energy projects are predominantly structured as stand-alone project companies, with lenders having no recourse to the project sponsors in case of default^61,72^. This financing structure creates a direct link between technology characteristics and overall risk profiles^14^, leading to WACCs varying within the same sector (electricity generation) by technology^73^. Therefore, we assess technology-specific WACC spreads using a recent database of renewable energy financing conditions^74^ and, based on the results in Supplementary Note 1, apply a conservative WACC premium of +0.8 percentage points to equation (1) for offshore and other not-yet-matured renewable energy technologies in IMAGE (that is, concentrated solar power and wave) vis-á-vis other electricity generation technologies (Supplementary Note 1).

### Country-to-region aggregation and sector mapping

We aggregate sector-specific WACC estimates from the country level to IMAGE model regions using World Bank WDI data on GDP adjusted for PPP^75^ for 2024 (or, if this is not available, the last available year) as weights. As a result, a handful of countries without available GDP information in the World Bank data (for example, Venezuela) are effectively excluded from regional averages. The same holds for countries that are missing in the Damodaran database altogether; however, these countries tend to be small and thus would have a very low weight in our GDP-weighted regional averages. For a few countries, corporate tax rates are missing. In this case, we take country-specific values from PwC’s Worldwide Tax Summaries^71^, and, if those are also not available, we impute missing country values with the GDP-weighted average tax rate in a country’s IMAGE region (Supplementary Table 10).

For sector-specific values with subscript *a* from the NYU Damodaran database (*β*, DP and DS), we map the sectors in IMAGE to the industry groups distinguished in the database (Supplementary Table 1). Where this is not straightforward, we identify the best corresponding industry by first identifying the major publicly listed firms worldwide in the respective sector of interest through desk research and then identifying the Damodaran database industry group of these firms, using the four-eyes principle. Notably, only publicly listed firms are included in the database and, hence, in Supplementary Table 1. For additional details, see Supplementary Note 1.

### Projecting changes in country risk premiums

Estimates for CRP in equation (1) reflect current financing conditions. Studies assessing the main determinants of sovereign credit ratings consistently rank (log-transformed) GDP per capita as the main predictor^36,37^, which will increase over time. Therefore, we take country risk premiums from ref. ^54^ for all available years starting in 2001 and combine them with World Bank data on real GDP per capita adjusted for PPP available through 2024^76^. Then, we regress the country risk premium for debt of country *i* in year *t* as follows:

2 $${\mathrm{CRP}}_{i,t}^{D}={\beta }_{1}{\mathrm{lnGDPpc}}_{i,t-1}+{\beta }_{2}{\mathrm{CRP}}_{i,t-1}^{D}+{\alpha }_{i}+{\delta }_{t}+{\epsilon }_{i,t},$$

where lnGDPpc denotes the log-transformed GDP per capita from the previous year (as CRP is based on start-of-year ratings), *α*_*i*_ and *δ*_*t*_ denote country and year fixed effects (*N* = 164 countries, *T* = 25 years, unbalanced panel) and *ϵ*_*i*,*t*_ is the error term. The model features a lagged value of the dependent variable (${\mathrm{CRP}}_{i,t-1}^{D}$) to account for the stickiness in country ratings, while further lags are not statistically significant but reduce the effective sample size (Supplementary Table 7). To ensure that the estimated effects are consistent with our GDP-weighted CoC estimates and can be applied to IMAGE regions that are primarily influenced by larger economies, our main specification weights countries by their current GDP^75^. This also avoids very small countries with unusual GDP per capita values driving our results. As robustness checks, we also weight countries by current population or equally, add a country-specific linear time trend, and exclude post-pandemic years by using only years before 2020 (Supplementary Table 6). Our conservative main specification suggests a 0.56 percentage point decrease in the country risk premium per one-unit increase in log-transformed GDP per capita (which ranges between 7 and 12). Additional information on the regressions, including the data used and the motivation for weighting observations and using fixed effects, is provided in Supplementary Note 3.

Notably, the relationship between country risk premium and log-transformed GDP per capita is broadly linear through lnGDPpc = 11 (that is, a GDP per capita of roughly US$60,000) before flattening out (Supplementary Fig. 4), in line with the extant literature^37^. This flattening-out effect cannot be captured by our fixed effects regression model since using country fixed effects effectively discards all countries for which the country risk premium is consistently at zero (for example, the USA). Therefore, we further assume that the relationship between log-transformed GDP per capita and the country risk premium no longer persists once this income level is reached.

Noting that differentiating equation (1) with respect to CRP^*D*^ yields

3 $${\psi }_{i,a}=1.35(1-{\mathrm{DS}}_{a})+{\mathrm{DS}}_{a}(1-{\tau }_{i}),$$

this means that we project future reductions in WACCs due to lower regional risk premiums for debt in IMAGE as follows:

4 $$\begin{array}{l}{\mathrm{WACC}}_{r,a,t}={\mathrm{WACC}}_{r,a,2025}-{\psi }_{r,a}\times 0.0056\times \left(\min \{11,\,{\mathrm{lnGDPpc}}_{r,t}\}\right.\\\qquad\qquad\quad\left.-\min \{11,\,{\mathrm{lnGDPpc}}_{r,2025}\}\right).\end{array}$$

Here, *r* denotes the IMAGE region in question, *ψ*_*r*,*t*,*a*_ is calculated at the country level following equation (3) and aggregated to the region level using GDP weighting (equivalent to the WACCs). In addition, we floor WACC_*r*,*a*,*t*_ at the value it would have in each region if the country risk premium were zero (CRP^*D*^ = 0). As a result, regions with relatively low current income levels can improve their financing conditions through continued economic growth. By contrast, regions relatively close to or above the cutoff value of 11 that are not AAA-rated (for example, Western Europe due to, among others, Italy) maintain their risk premium, which is in line with this premium being related to factors other than general economic development.

### Reporting summary

Further information on research design is available in the Nature Portfolio Reporting Summary linked to this article.

## Online content

Any methods, additional references, Nature Portfolio reporting summaries, source data, extended data, supplementary information, acknowledgements, peer review information; details of author contributions and competing interests; and statements of data and code availability are available at https://doi.org/10.1038/s41558-026-02756-0.

## Supplementary information

Supplementary Information Supplementary Notes 1–5, Figs. 1–24 and Tables 1–10.

Reporting Summary

## Source data

Source Data Figs. 1 and 3–5 Statistical source data for Figs. 1 and 3–5.

## Data Availability

Data from the NYU Damodaran database^35^ are available at https://pages.stern.nyu.edu/~adamodar/. Data from the Kroll Cost of Capital Navigator^63^ are proprietary and available only to subscribers, who can obtain access at https://www.kroll.com/en/tools-and-platforms/cost-of-capital. OECD data on US Treasury bond yields^64^ are available at https://fred.stlouisfed.org/series/IRLTLT01USM156N. PwC data on corporate tax rates^71^ are available at https://taxsummaries.pwc.com/interactive-map. GDP and GDP per capita data from the World Bank WDI database^75,76^ are available at https://databank.worldbank.org/source/world-development-indicators. All non-proprietary input data and derivatives used in this study (that is, excluding Kroll-based data used only for robustness checks), including the main CoC estimates and the IMAGE output data, are available via Zenodo at https://doi.org/10.5281/zenodo.21922274 (ref. ^77^). Source data are provided with this paper.

## Code availability

Information on the IMAGE model and the principles of its source code can be found in ref. ^33^. The code for the empirical analysis, for processing IMAGE outputs and for creating the figures and tables in this study is available via Zenodo at https://doi.org/10.5281/zenodo.21922274 (ref. ^77^).

## References

[1] Friedlingstein, P. et al. Global carbon budget 2024. *Earth Syst. Sci. Data* **17**, 965–1039 10.5194/essd-17-965-2025 (2025).

[2] Emissions gap report. *UNEP Knowledge Repository* 10.59117/20.500.11822/46404 (2024).

[3] IPCC *Climate Change 2022: Mitigation of Climate Change. Working Group III Contribution to the Sixth Assessment Report of the Intergovernmental Panel on Climate Change* (Cambridge Univ. Press, 2023).

[4] IPCC *Climate Change 2014: Mitigation of Climate Change. Contribution of Working Group III to the Fifth Assessment Report of the Intergovernmental Panel on Climate Change* (Cambridge Univ. Press, 2014).

[5] *Impact Assessment Report: Securing Our Future Europe’s 2040 Climate Target and Path to Climate Neutrality by 2050 Building a Sustainable, Just and Prosperous Society* (European Commission, 2024).

[6] Battiston, S., Monasterolo, I., Riahi, K. & van Ruijven, B. J. Accounting for finance is key for climate mitigation pathways. *Science* **372**, 918–920 10.1126/science.abf3877 (2021). 34016742

[7] McCollum, D. L. et al. Energy investment needs for fulfilling the Paris Agreement and achieving the Sustainable Development Goals. *Nat. Energy* **3**, 589–599 10.1038/s41560-018-0179-z (2018).

[8] *World Energy Outlook* (IEA, 2025).

[9] Keppo, I. et al. Exploring the possibility space: taking stock of the diverse capabilities and gaps in integrated assessment models. *Environ. Res. Lett.* **16**, 053006 10.1088/1748-9326/abe5d8 (2021).

[10] Ellenbeck, S. & Lilliestam, J. How modelers construct energy costs: discursive elements in energy system and integrated assessment models. *Energy Res. Soc. Sci.* **47**, 69–77 10.1016/j.erss.2018.08.021 (2019).

[11] Shiraki, H. & Sugiyama, M. Back to the basic: toward improvement of technoeconomic representation in integrated assessment models. *Clim. Change* **162**, 13–24 10.1007/s10584-020-02731-4 (2020).

[12] Krey, V. et al. Looking under the hood: a comparison of techno-economic assumptions across national and global integrated assessment models. *Energy* **172**, 1254–1267 10.1016/J.ENERGY.2018.12.131 (2019).

[13] Bertram, C. et al. Feasibility of peak temperature targets in light of institutional constraints. *Nat. Clim. Change* **14**, 954–960 10.1038/s41558-024-02073-4 (2024).

[14] Steffen, B. & Waidelich, P. Determinants of cost of capital in the electricity sector. *Prog. Energy* **4**, 033001 10.1088/2516-1083/ac7936 (2022).

[15] Schmidt, T. S. et al. Adverse effects of rising interest rates on sustainable energy transitions. *Nat. Sustain.* **2**, 879–885 10.1038/s41893-019-0375-2 (2019).

[16] Hirth, L. & Steckel, J. C. The role of capital costs in decarbonizing the electricity sector. *Environ. Res. Lett.* **11**, 114010 10.1088/1748-9326/11/11/114010 (2016).

[17] Ondraczek, J., Komendantova, N. & Patt, A. WACC the dog: the effect of financing costs on the levelized cost of solar PV power. *Renew. Energy* **75**, 888–898 10.1016/j.renene.2014.10.053 (2015).

[18] Schmidt, T. S. Low-carbon investment risks and de-risking. *Nat. Clim. Change* **4**, 237–239 10.1038/nclimate2112 (2014).

[19] Barnes, J. & Bhagavathy, S. M. The economics of heat pumps and the (un)intended consequences of government policy. *Energy Policy* **138**, 111198 10.1016/j.enpol.2019.111198 (2020).

[20] Pahle, M. et al. Safeguarding the energy transition against political backlash to carbon markets. *Nat. Energy* **7**, 290–296 10.1038/s41560-022-00984-0 (2022).

[21] Lonergan, K. E. et al. Improving the representation of cost of capital in energy system models. *Joule*10.1016/j.joule.2023.02.004 (2023).

[22] Neuwirth, M., Fleiter, T. & Hofmann, R. Modelling the transformation of energy-intensive industries based on site-specific investment decisions. *Sci. Rep.* **14**, 30552 10.1038/s41598-024-78881-7 (2024). PMC11655855 39695303

[23] Sanders, M. et al. Representation of financial markets in macro-economic transition models—a review and suggestions for extensions. *Environ. Res. Lett.* **17**, 083001 10.1088/1748-9326/ac7f48 (2022).

[24] Hafner, S., Anger-Kraavi, A., Monasterolo, I. & Jones, A. Emergence of new economics energy transition models: a review. *Ecol. Econ.* **177**, 106779 10.1016/j.ecolecon.2020.106779 (2020).

[25] Egli, F., Steffen, B. & Schmidt, T. S. Bias in energy system models with uniform cost of capital assumption. *Nat. Commun.* **10**, 4588 10.1038/s41467-019-12468-z (2019). PMC6785535 31597921

[26] Briera, T. & Lefèvre, J. Reducing the cost of capital through international climate finance to accelerate the renewable energy transition in developing countries. *Energy Policy* **188**, 114104 10.1016/j.enpol.2024.114104 (2024).

[27] Calcaterra, M. et al. Reducing the cost of capital to finance the energy transition in developing countries. *Nat. Energy* **9**, 1241–1251 10.1038/s41560-024-01606-7 (2024). PMC11496104 39450347

[28] Polzin, F. et al. The effect of differentiating costs of capital by country and technology on the European energy transition. *Clim. Change* **167**, 26 10.1007/s10584-021-03163-4 (2021).

[29] Schyska, B. U. & Kies, A. How regional differences in cost of capital influence the optimal design of power systems. *Appl. Energy* **262**, 114523 10.1016/j.apenergy.2020.114523 (2020).

[30] Ameli, N. et al. Higher cost of finance exacerbates a climate investment trap in developing economies. *Nat. Commun.* **12**, 4046 10.1038/s41467-021-24305-3 (2021). PMC8245630 34193868

[31] Sweerts, B., Longa, F. D. & van der Zwaan, B. Financial de-risking to unlock africa’s renewable energy potential. *Renew. Sustain. Energy Rev.* **102**, 75–82 10.1016/j.rser.2018.11.039 (2019).

[32] Damodaran, A. *Damodaran Dataset: Levered and Unlevered Betas by Industry* (version: January 2025 update; file path: Data > archived data > risk/discount rate > levered and unlevered betas by industry > global > 1/25) (New York Univ., 2025).

[33] IMAGE framework. *PBL* https://models.pbl.nl/image/IMAGE_framework (2022).

[34] Falchetta, G., Dagnachew, A. G., Hof, A. F. & Milne, D. J. The role of regulatory, market and governance risk for electricity access investment in sub-saharan africa. *Energy Sustain. Dev.* **62**, 136–150 10.1016/j.esd.2021.04.002 (2021).

[35] Damodaran, A. *Damodaran Dataset* (version: January 2025 update) (New York Univ., 2025).

[36] De Moor, L., Luitel, P., Sercu, P. & Vanpee, R. Subjectivity in sovereign credit ratings. *J. Bank. Finance* **88**, 366–392 10.1016/j.jbankfin.2017.12.014 (2018).

[37] Klusak, P., Agarwala, M., Burke, M., Kraemer, M. & Mohaddes, K. Rising temperatures, falling ratings: the effect of climate change on sovereign creditworthiness. *Manag. Sci.* **69**, 7151–7882 10.1287/mnsc.2023.4869 (2023).

[38] Cantor, R. M. & Packer, F. Determinants and impact of sovereign credit ratings. *Econ. Policy Rev.* **2**, 37–53 10.2139/ssrn.1028774 (1996).

[39] Ekholm, T., Ghoddusi, H., Krey, V. & Riahi, K. The effect of financial constraints on energy-climate scenarios. *Energy Policy* **59**, 562–572 10.1016/j.enpol.2013.04.001 (2013).

[40] Klaaßen, L. & Steffen, B. Meta-analysis on necessary investment shifts to reach net zero pathways in Europe. *Nat. Clim. Change* **13**, 58–66 10.1038/s41558-022-01549-5 (2023).

[41] Egli, F. et al. Mapping cost competitiveness of African green hydrogen imports to europe. *Nat. Energy* **10**, 750–761 10.1038/s41560-025-01768-y (2025).

[42] Bachorz, C., Verpoort, P. C., Luderer, G. & Ueckerdt, F. Exploring techno-economic landscapes of abatement options for hard-to-electrify sectors. *Nat. Commun.* **16**, 3984 10.1038/s41467-025-59277-1 (2025). PMC12037817 40295519

[43] Fennell, P., Driver, J., Bataille, C. & Davis, S. J. Cement and steel—nine steps to net zero. *Nature* **603**, 574–577 10.1038/d41586-022-00758-4 (2022). 35322242

[44] Sun, Y. et al. Decarbonising the iron and steel sector for a 2 °C target using inherent waste streams. *Nat. Commun.* **13**, 297 10.1038/s41467-021-27770-y (2022). PMC8758725 35027534

[45] van Sluisveld, M. A., de Boer, H. S., Daioglou, V., Hof, A. F. & van Vuuren, D. P. A race to zero—assessing the position of heavy industry in a global net-zero CO_2_ emissions context. *Energy Clim. Change* **2**, 100051 10.1016/j.egycc.2021.100051 (2021).

[46] Fisch-Romito, V., Jaxa-Rozen, M., Wen, X. & Trutnevyte, E. Multi-country evidence on societal factors to include in energy transition modelling. *Nat. Energy* **10**, 460–469 10.1038/s41560-025-01719-7 (2025). PMC12021648 40291482

[47] Toetzke, M., Stünzi, A. & Egli, F. Consistent and replicable estimation of bilateral climate finance. *Nat. Clim. Change* **12**, 897–900 10.1038/s41558-022-01482-7 (2022).

[48] McCollum, D. L. et al. Improving the behavioral realism of global integrated assessment models: an application to consumers’ vehicle choices. *Transp. Res. Part D* **55**, 322–342 10.1016/j.trd.2016.04.003 (2017).

[49] Verpoort, P. C., Gast, L., Hofmann, A. & Ueckerdt, F. Impact of global heterogeneity of renewable energy supply on heavy industrial production and green value chains. *Nat. Energy* **9**, 491–503 10.1038/s41560-024-01492-z (2024).

[50] Damodaran, A. Equity risk premiums (ERP): determinants, estimation, and implications: the 2025 edition. *SSRN*10.2139/ssrn.5168609 (2025).

[51] Semieniuk, G. et al. Stranded fossil-fuel assets translate to major losses for investors in advanced economies. *Nat. Clim. Change* **12**, 532–538 10.1038/s41558-022-01356-y (2022).

[52] Mandel, A., Battiston, S. & Monasterolo, I. Mapping global financial risks under climate change. *Nat. Clim. Change* **15**, 329–334 10.1038/s41558-025-02244-x (2025).

[53] Polzin, F., Egli, F., Steffen, B. & Schmidt, T. S. How do policies mobilize private finance for renewable energy? A systematic review with an investor perspective. *Appl. Energy* **236**, 1249–1268 10.1016/j.apenergy.2018.11.098 (2019).

[54] Damodaran, A. *Damodaran Dataset: Risk Premiums for Other Markets* (version: January updates for 2016–2025; file path: Data > archived data > risk/discount rate > risk premiums for other markets > 1/25, 1/24, etc.) (New York Univ., 2025).

[55] Massicotte, P., South, A. & Hufkens, K. rnaturalearth: world map data from Natural Earth. R version 1.1.0 (2025).

[56] Xylia, M., Silveira, S., Duerinck, J. & Meinke-Hubeny, F. Weighing regional scrap availability in global pathways for steel production processes. *Energy Effic.* **11**, 1135–1159 10.1007/s12053-017-9583-7 (2018).

[57] Kermeli, K. et al. Improving material projections in integrated assessment models: the use of a stock-based versus a flow-based approach for the iron and steel industry. *Energy* **239**, 122434 10.1016/j.energy.2021.122434 (2022).

[58] Nesje, F., Drupp, M. A., Freeman, M. C. & Groom, B. Philosophers and economists agree on climate policy paths but for different reasons. *Nat. Clim. Change* **13**, 515–522 10.1038/s41558-023-01681-w (2023).

[59] Dafnomilis, I., den Elzen, M. & van Vuuren, D. P. Achieving net-zero emissions targets: an analysis of long-term scenarios using an integrated assessment model. *Ann. N. Y. Acad. Sci.* **1522**, 98–108 10.1111/nyas.14970 (2023). 36841927

[60] Forster, P. M. et al. Indicators of global climate change 2022: annual update of large-scale indicators of the state of the climate system and human influence. *Earth Syst. Sci. Data* **15**, 2295–2327 10.5194/essd-15-2295-2023 (2023).

[61] *The Cost of Financing for Renewable Power* (IRENA, 2023).

[62] Damodaran, A. *Investment Valuation: Tools and Techniques for Determining the Value of Any Asset* 4th edn (Wiley, 2025).

[63] Cost of capital navigator. *Kroll* https://www.kroll.com/en/tools-and-platforms/cost-of-capital (2026).

[64] *Interest Rates: Long-Term Government Bond Yields: 10-Year: Main (Including Benchmark) for United States (IRLTLT01USM156N)*, retrieved from FRED, Federal Reserve Bank of St. Louis (OECD, 2026).

[65] Almeida, H., Cunha, I., Ferreira, M. A. & Restrepo, F. The real effects of credit ratings: the sovereign ceiling channel. *J. Finance* **72**, 249–290 10.1111/jofi.12434 (2017).

[66] Damodaran, A. Country risk: determinants, measures, and implications—the 2024 edition. *SSRN*10.2139/ssrn.4896539 (2024).

[67] Dimson, E., Marsh, P. & Staunton, M. *UBS Global Investment Returns Yearbook 2024* (UBS, 2024).

[68] Pratt, S. P. & Grabowski, R. J. *Cost of Capital: Applications and Examples* 5th edn (Wiley, 2014).

[69] Damodaran, A. *Damodaran Dataset: Costs of Capital by Industry (in US $)* (version: January 2025 update; file path: Data > archived data > risk/discount rate > costs of capital by industry (in US $) > global > 1/25) (New York Univ., 2025).

[70] Damodaran, A. *Damodaran Dataset: Risk Premiums for Other Markets* (version: January 2025; file path: Data > archived data > risk/discount rate > risk premiums for other markets > 1/25) (New York Univ., 2025).

[71] *Worldwide Tax Summaries [Headline CIT Rate]* (PwC, 2026).

[72] Steffen, B. The importance of project finance for renewable energy projects. *Energy Econ.* **69**, 280–294 10.1016/j.eneco.2017.11.006 (2018).

[73] Steffen, B. Estimating the cost of capital for renewable energy projects. *Energy Econ.* **88**, 104783 10.1016/j.eneco.2020.104783 (2020).

[74] Steffen, B., Egli, F., Gumber, A., Dukan, M. & Waidelich, P. A global dataset of the cost of capital for renewable energy projects. *Sci. Data* **12**, 1624 10.1038/s41597-025-05912-x (2025). PMC12508428 41062538

[75] World development indicators [GDP PPP in constant 2021 international $, series code NY.GDP.MKTP.PP.KD]. Washington, DC. *World Bank* https://databank.worldbank.org/source/world-development-indicators (2025).

[76] World development indicators [GDP per capita, PPP in constant 2021 international $, series code NY.GDP.PCAP.PP.KD]. Washington, DC. *World Bank* https://databank.worldbank.org/source/world-development-indicators (2025).

[77] Waidelich, P. et al. Scripts and data for “the impact of financing cost differences on global energy and industry decarbonization”. *Zenodo*10.5281/zenodo.21922274 (2026).

