# Supplementary material for: The impact of financing cost differences on global energy and industry decarbonization
Source: Nat Clim Chang. 2026 Sep 24;16(10):1178–86. doi: 10.1038/s41558-026-02756-0 (PMC13638454; doi:10.1038/s41558-026-02756-0)
Supplement: Supplementary file 1 [file 41558_2026_2756_MOESM1_ESM.pdf]

nature climate change

Supplementary information https://doi.org/10.1038/s41558-026-02756-0

The impact of financing cost differences on global energy and industry decarbonization

In the format provided by the authors and unedited

Supplementary Note 1: Additional information on the CoC estimates

	IMAGE sector	Industry in the Damodaran database	Unlevered beta	Equity share	Publicly listed example companies
1	Electricity	Power	0.41	51%	NextEra, The Southern Company, Enel, Uniper, Iberdrola, RWE, EnBW, Fortum, Korea Electric Power Corporation, Tokyo Electric Power<br>*Note: Some companies (e.g., Engie) are also listed as "Utility (General)", which has the same beta and only a slightly higher equity share (56%).*
2	Electricity networks	Power	0.41	51%	Redeia, Terna, Elia, Admie, Fortis, Xcel Energy, Power Grid Corporation of India Power Grid Bangladesh, Hydro One<br>*Note: Some companies (e.g., National Grid, E.ON) are also listed as "Utility (General)".*
3	Centralized heat generation	Power	0.41	51%	Fortum, Uniper, EnBW, Orsted<br>*Note: Some companies (e.g., Engie, Veolia, E.ON) are also listed as "Utility (General)"*
4	Hydrogen production	Chemical (Specialty)	0.89	81%	Linde, Air Products, Air Liquide, Jiangsu Huachang<br>*Note: Some key companies are also listed as "Chemical (Diversified)" (BASF; beta: 0.77, equity share: 61%) or "Oil/Gas Integrated" (Sinopec; beta: 0.81, equity share: 83%)*
5	Biomass production	Oilfield Svcs/Equip.	0.70	68%	*For bioethanol:* Valero, Green Plains, Cosan, CropEnergies, Aemetis, Cardinal Ethanol, Highwater Ethanol, Ubon Bio Ethanol<br>*For biodiesel:* Renewable Energy Group, FutureFuel, Longyan Zhuoyue New Energy, Aemetis, Neste<br>*Note: Some companies (ADM, Wilmar, 3Tentos) are also listed as "Farming/Agriculture" (beta: 0.52, equity share: 61%). Leading pellet producers are listed either as "Coal & Related Energy" (Enviva; beta: 1.05, equity share: 84%) or "Green & Renewable Energy" (Drax, which primarily deals in power generation; beta: 0.57, equity share: 58%)*
6	Cement	Construction Supplies	0.76	73%	Holcim, CEMEX, CRH, Buzzi, UltraTech, Grasim, Anhui Conch, Heidelberg Cement, Ambuja
7	Food processing	Food Processing	0.54	73%	Nestle, Mondelez, Hershey, Kraft Heinz, Danone, General Mills, Lindt & Sprüngli<br>*Note: Some key companies (Unilever) are also listed as "Transportation" (beta: 0.65, equity share: 64%)*
8	Iron and steel	Steel	0.82	65%	ArcelorMittal, Nippon Steel, HBIS, Shagang, POSCO, Shougang, Tata Steel, JSW Steel Limited, JFE, Nucor
9	Pulp and paper	Paper/Forest Products	0.54	55%	UPM-Kymmene, Suzano, Svenska Cellulosa, Stora Enso, Celulosa Argentina, Mondi, Holmen<br>*Note: Some companies (International Paper) are also listed as "Food Processing"*

Table S1. Matching of climate-relevant technologies/subsectors to the industry groups in the NYU Damodaran database

Corporate and project finance and their relationship with the CoC The equity shares in Supplementary Table S1 are based on average indebtedness levels of companies. Using them for CoC estimates assumes that investments are financed on companies' balance sheets ("corporate finance"), as opposed to "project finance", the creation of special purpose vehicle project companies that raise debt capital themselves, with no recourse to project sponsors in case of default.[1] While corporate finance typically dominates private sector investments,[2] project finance is common in chemical plants, mines, transport and telecommunications infrastructure[1] and is the predominant financing mode for renewable energy projects[3]. As project finance is far less common in other areas like industry decarbonization, we apply corporate finance-based debt shares across all sectors in our scope to obtain comparable and consistent estimates.

In general, project finance typically features a higher debt share of around 70–80%, facilitated by better corporate governance, easier assessments by lenders, and the signing of long-term power purchase agreements that reduce market risk[1]. The cost of debt is typically lower than the cost of equity.[4] However, this does not necessarily imply that project finance-based CoC would be lower than the estimates in our study because a higher debt share increases the risks to equity investors due to a higher default risk[4]. This is reflected in the relevering formula provided in our Methods section:

$$\beta_{i,a} = \beta_{a,\text{unlevered}} \times \left[1 + (1-\tau_i) \times \frac{\text{DS}_a}{1-\text{DS}_a}\right] \qquad \text{(S1)}$$

where τ denotes the corporate tax rate, DS is the debt share of a sector a and $\beta_{a,\text{unlevered}}$ is the unlevered beta that does not include indebtedness risk. A higher debt share will increase the $\frac{\text{DS}_a}{1-\text{DS}_a}$ term and thus increase the levered beta which determines our cost of equity estimates (see Methods). Therefore, imputing project finance-like debt

Table S2. US CoC estimates by sector and IMAGE CO2 emissions (Uniform CoC, Current Policies) by year in rounded MtCO2, as well as emission-weighted average across sectors. Note that hydrogen generation emissions do not include production for legacy use cases.

	Sector	CoC (nominal, after-tax)	2010	2025	2050
1	Electricity	4.52%	10349	11848	9856
2	Heat	4.52%	1620	1827	1142
3	Food processing	5.22%	469	446	625
4	Pulp and paper	5.26%	221	151	111
5	Biomass	5.99%	1203	1267	895
6	Cement	6.28%	1065	1089	283
7	Iron and steel	6.53%	2299	2547	1715
8	Hydrogen	6.88%	0	8	94
9	Weighted average		5.03%	5.01%	4.93%

shares without explicit de-risking measures would not yield substantially lower CoC estimates than those presented here.

Technology risk premium for higher-risk, project-financed renewable energy technologies. Overall, our methodology to estimate the CoC assumes on average similar financing conditions for projects carried out by the same sponsors and, therefore, estimates sector-level CoC based on sector-specific average risks and indebtedness (see Methods). Unlike fossil fuel-based technologies and most industries, however, power generation using renewable energy (RE) technologies is predominantly financed via non-recourse project finance (for details on this financing structure, we refer readers to the previous paragraph). Therefore, for the same project sponsors, financing conditions can and do vary by technology due to differences in risk-return profiles related to time-constant technology-inherent characteristics and time-varying market maturity[5, 6]. Previous studies have addressed this by estimating RE CoC by technology, for instance, by using utility-specific financial and portfolio data, using country-level deployment as proxies for market maturity with thresholds informed by expert interviews, or estimating technology differences based on average CoC spreads between technology-specific CoC estimates in the extant literature[7, 8]. However, the data underlying these estimates is somewhat outdated as of early 2026. Given the substantial pace of global solar and wind energy deployment, this risks an upward bias in immaturity-related CoC premiums (e.g., if immaturity-related premiums based on 2020 data are applied to technologies' CoC in 2025 or 2030). In addition, piecing together CoC estimates from studies that use different assumptions for key parameters, such as the risk-free rate, leverage, and equity risk premium, would undermine the internal consistency of our CoC estimates across sectors.

While the power generation sector is not the primary focus of this paper, we investigate how recent CoC estimates suggest the need for technology-specific risk premiums. Specifically, we leverage a recently published database of CoC estimates for solar PV, onshore and offshore wind for 68 countries between 2010–2022[9] and regress the available nominal after-tax WACC estimates on technology dummies, adding country, year, and study fixed effects to reduce omitted variable bias. To ensure that findings are not driven by outdated data points (reflecting a market immaturity that may no longer apply) or methodological differences between data points for different technologies, we also explore results for subsetting the sample to estimates for the most recent years and to studies that cover all three technologies (i.e., the fixed effects model effectively averages within-study between-technology spreads).

Results are shown in Supplementary Table S3 and indicate that, on average, offshore wind commands the highest CoC of the technologies, while onshore wind commands the lowest. This finding aligns with previous studies which document that onshore wind matured the earliest among the three RE technologies, while solar PV's technology risks have decreased substantially throughout the 2000s and 2010s amid a sharp ramp-up in deployment[10, 11] and, in some markets like Germany, converged against those of onshore wind[12]. By contrast, offshore wind is considered a high-CoC technology due to construction complexities and ownership structures, with previous studies documenting a CoC premium of +1.3 percentage points[13] and widespread public financial support

for the technology[11, 14]. Similarly, our estimates for offshore wind vary between 0.8–1.4 percentage points across specifications but remain statistically significant. By contrast, the p-value for the solar PV premium exceeds 5% if we subset the sample to the most recent years and becomes statistically insignificant and lower if we only consider studies covering all three RE technologies.

Table S3. Assessing technology spreads in published CoC estimates

Dependent Variable:	WACC (nominal, after-tax) in decimals			
	Full Sample	2019–	All 3 Tech	All 3 Tech (2019–)
Model:	(1)	(2)	(3)	(4)
Variables				
technology = SolarPV	0.0051*	0.0071.	0.0032	0.0032
	(0.0024)	(0.0038)	(0.0023)	(0.0020)
technology = Windoffshore	0.0144***	0.0105**	0.0081**	0.0080**
	(0.0036)	(0.0034)	(0.0027)	(0.0027)
Fixed-effects				
country	Yes	Yes	Yes	Yes
financing_year	Yes	Yes	Yes	Yes
study	Yes	Yes	Yes	Yes
Fit statistics				
Observations	366	150	39	30
R^2	0.76441	0.82969	0.86869	0.91288
Within R^2	0.04106	0.05778	0.29717	0.37524
BIC	-1,347.3	-541.27	-238.08	-198.38

Heteroskedasticity-robust standard-errors in parentheses
*Signif. Codes: ***: 0.001, **: 0.01, *: 0.05, .: 0.1*

Doing so effectively reduces the sample to selected OECD countries, China, and Vietnam, in which offshore wind and solar PV deployment is relatively advanced compared to most markets. Therefore, using this subset implies a lower risk of conflating technology-specific, time-constant risk premiums with premiums due to (past) market immaturity that may have changed by now or may change in the near-term future. Therefore, we apply a conservative +0.8 percentage point CoC premium to offshore wind projects but no similar premium to solar PV. This is consistent with the approach in the IEA World Energy Outlook[15], which applies a 1 percentage point CoC premium to offshore wind vis-à-vis onshore wind and solar PV. Offshore wind featured a technology readiness level (TRL) of nine through 2022 in the International Energy Agency's technology classification (on a scale between 1–11)[16]. Therefore, we also apply the +0.8 percentage point premium to IMAGE's other less mature higher-risk renewable energy technologies with a current IEA TRL of nine or lower (i.e., concentrated solar power, and wave power) because for these technologies, a lack of technology-specific CoC estimates hinders the estimation of robust technology premiums.

Importantly, such a 0.8 percentage point premium represents a stylized approach that abstracts from potential heterogeneity in legal structures, tax codes, or equity shares required by lenders. In addition, such a 0.8 percentage point premium may be optimistic in early stages of market ramp-up in particular (e.g., for novel offshore wind markets) and some studies[8, 17, 3] have opted for dynamic, deployment-dependent immaturity premiums. However, a more granular representation of higher early-stage premiums (with estimates being a few percentage points higher) is unlikely to alter our conclusions for three reasons. First, offshore wind is the only technology that IMAGE deploys at scale, whereas the shares of wave power or CSP in global electricity production never exceed 1% across our different

CoC approaches (uniform or differentiated) and mitigation scenarios (current policies or well below 2°C). Therefore, cost estimates for these two technologies are not material for model outcomes. Second, previous studies indicate that renewable energy deployment in IAMs is relatively robust to moderate CoC changes[8], as rapid grid decarbonization is a necessary prerequisite for reaching net-zero; therefore, offshore wind deployment will likely occur in well below 2°C scenarios even if higher CoC increase deployment costs. Third and somewhat relatedly, offshore wind is a key technology in many regions under the well below 2°C run and will be deployed at scale, meaning that the capacity shares indicating commercial maturity are reached within a few model timesteps. For instance, the 6% share of installed capacity to qualify as fully mature offshore wind markets used by IRENA (2023) is reached globally between 2040 and 2045 in our well below 2°C runs. For these reasons, deploying more dynamic immaturity-related premiums may increase power system costs in the short term but are unlikely to affect our long-term results and conclusions. For numerical estimates of how early-stage market immaturity may increase technologies' CoC and how this may alter IAMs' power sector outcomes, we refer readers to IRENA (2023)[3], Hatton et al. (2025)[17] and Calcaterra et al. (2024)[8].

Importantly, the results in Supplementary Table S3 do not tell us how lower-risk renewable energy technologies compare with technologies that do not primarily use project finance, such as fossil fuels and nuclear. Project-financed projects generally use higher debt shares of around 70–80% because lenders can assess them more easily, corporate governance tends to be stronger, and long-term power purchase agreements reduce market risk[1]. However, as shown above, mechanical debt share increases have only a limited effect on our CoC estimates. Current CoC estimates for RE technologies also reflect de-risking policies (e.g., feed-in tariffs) that may be scaled back during IMAGE's modeling horizon. For these reasons, we apply our general power generation CoC estimate to lower-risk project-financed renewable energy technologies in IMAGE (i.e., solar PV, onshore wind, and biomass), noting that past IAM studies suggest that RE deployment in decarbonization scenarios is relatively insensitive to moderate CoC changes[8].

Sectoral mapping for biomass production For the biomass sector, publicly listed companies fall into various database categories with no clear-cut match. However, we note that the derived unlevered beta 0.70 and equity share of 68% (resulting in a relevered beta of 0.96 for a corporate tax rate of 20%) based on most companies' categorization aligns with company-specific levered beta estimates sourced from Yahoo Finance on 21 May 2025 for the largest companies, such as ADM (0.67), Green Plains (1.19), Valero (0.95), FutureFuel (0.84), Drax (0.75), and Enviva (0.84)—particularly when keeping in mind that Drax and ADM are primarily active in sectors like electricity generation and food processing, which have lower sectoral risk[18].

Alternative approaches to estimating the sectoral betas Our main CoC estimates use recent, global beta estimates by sector. The decision to use global estimates across countries and IMAGE regions is primarily driven by data availability and reliability. First, the databases we use here[19, 20] do not provide sector- and country-specific betas for a large sample of countries. Second, even if region-specific estimates are available, they are based on much smaller samples and hence more volatile. For instance, the Damodaran database[19] provides region-specific betas only for the US, China, (Western) Europe, Japan, India, Australia/New Zealand/Canada (grouped together) and a broad "Emerging Markets" category that combines extremely different markets and circumstances. To illustrate this, the global power sector beta is based on nearly 500 stocks, but this drops to 25-35 for some regions (e.g., India or Japan). In addition, estimated betas of emerging markets with limited stock market depth and liquidity tend to be low for purely mathematical reasons[21, 4, 22].

To investigate if using global betas drives our findings, we take the region-specific betas from the Damodaran database. We apply country-specific betas to respective countries (China, Japan, United States, Australia, New Zealand, Canada), impute the Japanese estimate for Korea as a geographically close high-income market, use the European beta for IMAGE's Western Europe region, and apply the emerging market estimate to all other IMAGE regions. The resulting WACCs are shown in Supplementary Fig. S1. Overall, using global and region-specific betas yields near-identical results for the rest-of-world category, while for the other regions, differences can be more substantial. Therefore, Supplementary Note 4 features a robustness check for which we use these region-specific instead of global betas for our *Differentiated CoC* scenario, which would not alter any of our main conclusions.

However, compiling and harmonizing region- or country-specific beta estimates by climate-relevant sector (or even technology) is a promising avenue for future research to address current data availability and reliability issues in capturing sectoral risks in IAMs and energy-system models.

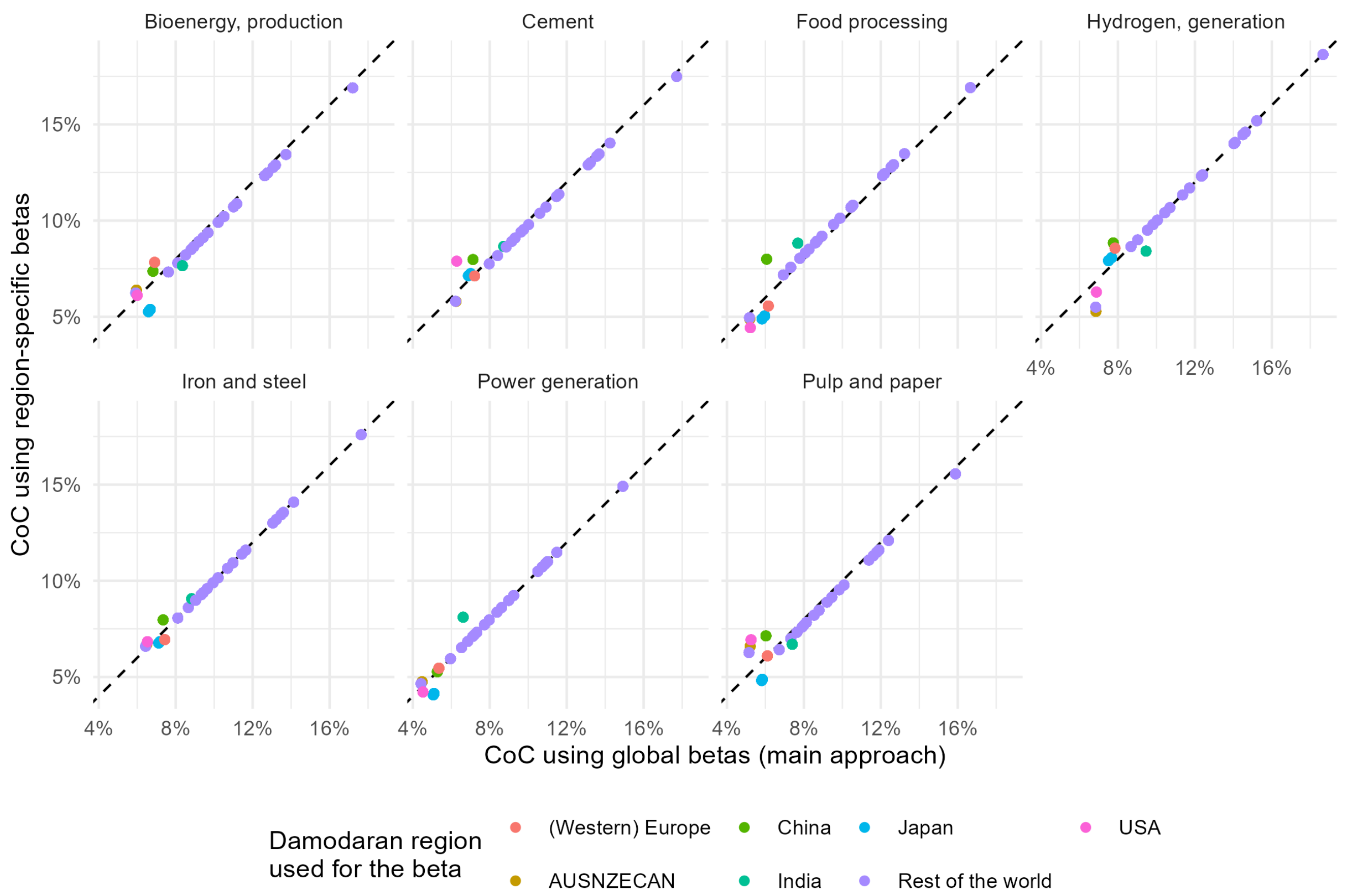

Figure S1. Comparison of WACC estimates (after-tax, nominal) using global average betas (i.e., our main approach) and using region-specific betas. Dashed line denotes the 45-degree line.

In addition, our main approach uses the recent 2025 beta estimates in the Damodaran database[19], which rely on up to five years of data, as is standard practice[23, 4]. To ensure that distortions in stock price volatility over the past years (including the COVID-19 pandemic and the energy price shock) do not drive our findings, we compare these 2025 beta estimates to Damodaran estimates over the past ten years. As Supplementary Fig. S2 shows, results are very similar, with the only notable exception being the biomass generation sector. As with the region-specific betas, Supplementary Note 4 also includes a robustness check when we use ten-year averages of (global) betas in our *Differentiated CoC* scenario, which would not alter any of our main conclusions.

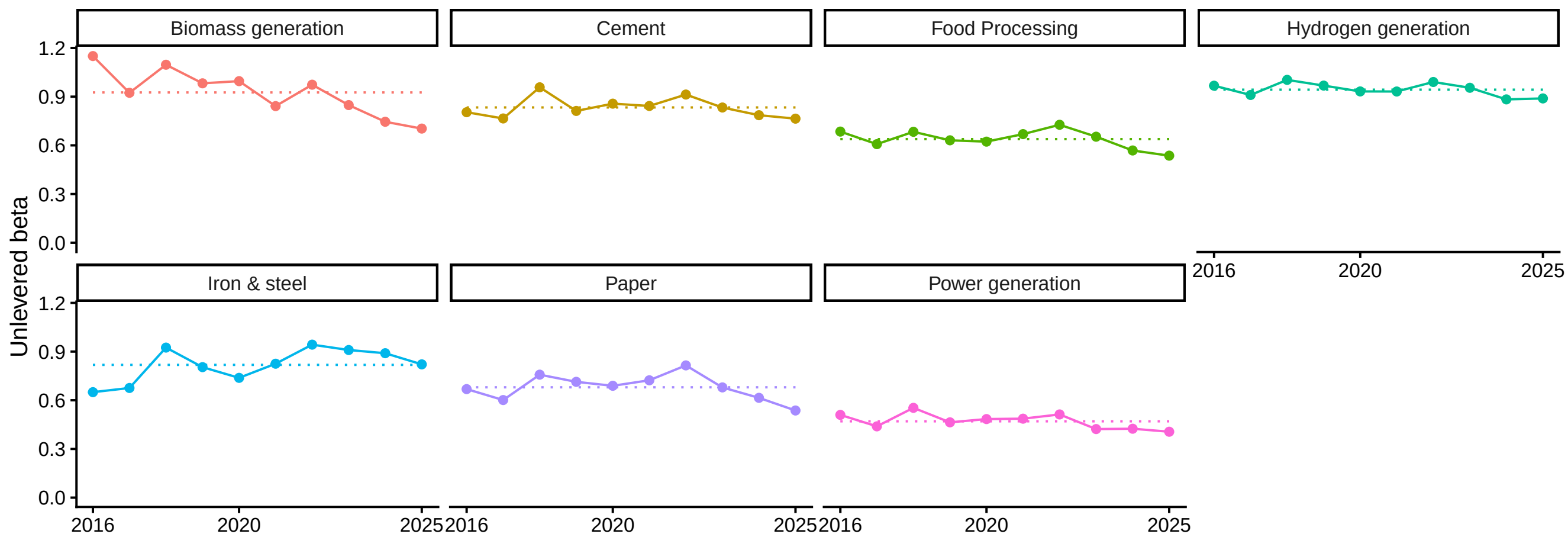

Figure S2. Unlevered annual beta estimates from the Damodaran database over 2016–2025 and their corresponding 2016–2025 average (dotted line).

Alternative approach to estimating debt premiums Damodaran[4] outlines three ways of estimating a company's cost of debt: i) its rating and/or the rates on its traded bonds, ii) the rates in its (recent) borrowing history, or iii) a synthetic rating based on some financial ratios. As many companies are not rated particularly in emerging markets, and pricing of past borrowings is typically confidential, approaches i) and ii) do not scale to a global coverage of entire industries. Therefore, the Damodaran debt premiums we use for our main estimates rely on the synthetic ratings approach, using the industry-specific standard deviations of stock prices over the prior two years as a proxy for risk. The underlying notion is that a higher stock price volatility indicates higher risk, which, when averaged across all firms in a sector, should correspond to higher credit risk.

To investigate if alternative approaches would alter our findings, we compare our main CoC estimates to alternative estimates when using sector-agnostic premiums (2.1% following ref.[8] or the 1.47% that ref.[4] provides as an average premium for BBB-rated companies; see their Table 8.6). Lastly, we use an alternative proxy for synthetic ratings, namely the interest coverage ratio (ICR), which is also included in the Damodaran database and which we convert to debt premiums using average premiums by ICR buckets in ref.[4], Table 8.6. Results are shown in Supplementary Fig. S3 and show that the resulting CoC estimates differ by 0.3 percentage points or less for each sector and leave the risk order across sectors unchanged. Therefore, alternative estimates of debt premiums are unlikely to affect our conclusions.

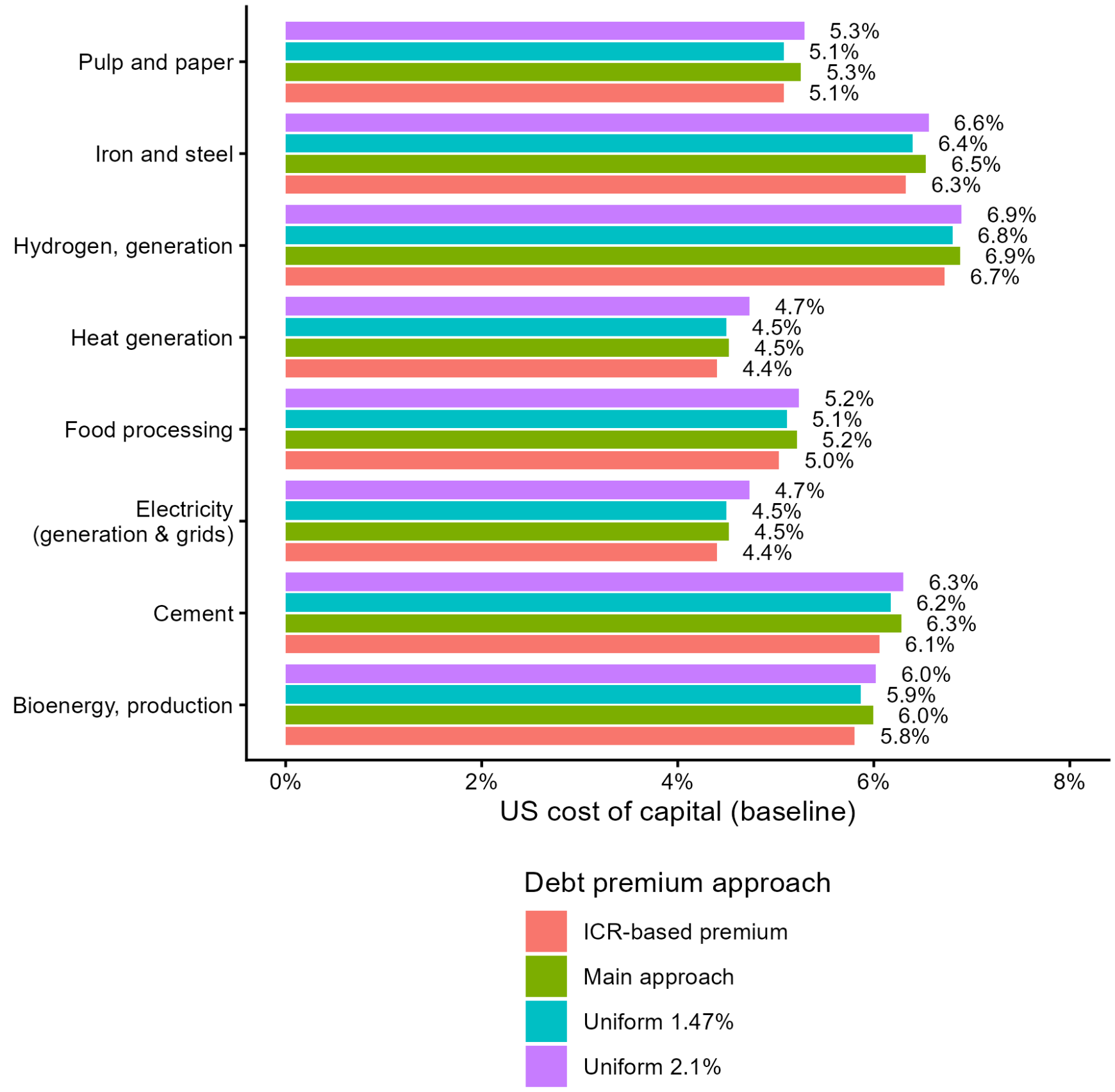

Figure S3. CoC estimates for the US in 2025 using our main approach and alternative approaches to estimate the debt premium by sector.

Motivating the uniform low-risk CoC benchmark The level of uniform CoC assumptions used in IAMs varies across models. Bachner et al. (2019)[24] review WACC assumptions across models (see their Table A1) and report a range of 5–10%. IMAGE has a legacy CoC assumption of 10%. The model was created in 1990 and the 10% CoC assumption (used as a discount rate to convert capital expenditures into annuities) is consistent with the interest rate environment at the time, which was considerably higher than today's levels.[25] By contrast, modelers in the 2010s, including the IEA and IRENA, often used CoC assumptions of 7–8%[26], which is close to the official discount rate guidance by the US government motivated by average private-sector return levels.[27] Due to lower interest rate levels over the past decade, our empirical estimates for electricity generation are considerably lower than these 7–8% assumptions.

We choose a 5% benchmark in our paper for three reasons. First, using a uniform CoC benchmark based on low-risk markets follows the existing literature on CoC effects[28, 29, 30]. Second, it mirrors the common practice in IAMs (and energy system models) of applying a uniform CoC or discount rate that is consistent with low-risk countries, such as the US. By contrast, comparing results to IMAGE's 10% legacy CoC would conflate CoC differentiation by sector/country with substantial interest rate level shifts. Third, a flat 5% CoC/discount rate for low-risk markets is in line with recent multi-model papers and assessments.[31, 32, 28] As a robustness check, Supplementary Note 4 includes an alternative *Uniform high-risk CoC* benchmark of 10% based on IMAGE's legacy CoC assumption and the higher end of the range used by IAMs.[24]

Supplementary Note 2: Robustness check using CoC estimates based on the Kroll Cost of Capital Navigator

Our main estimates primarily lean on the Damodaran database[19], which has been used extensively in the extant literature on CoC in energy system models and integrated assessment models[3, 8, 28, 17, 33], as well as other fields[34] and proprietary providers of CoC data[35]. To ensure that this does not drive our conclusions, we carry out a robustness check using the commercial Kroll CoC Navigator™[20], which is described in more detail by ref.[22].

Like the Damodaran database, Kroll provides easy-to-use industry-specific estimates for cost of capital components, is regularly updated, has detailed methodology descriptions and provides country-specific CoC differentiation for over 170 countries via country risk premiums (CRPs).[1] Unlike the Damodaran data, however, the Kroll database is subscription-based and its license restricts redistribution, which limits accessibility for researchers and the scope for data sharing and reproducibility. Therefore, we use the Kroll CoC Navigator for a comprehensive robustness check but report only correlations and signed differences between Kroll- and Damodaran-based estimates instead of reproducing underlying CoC components and aggregates. However, we encourage readers to contact the corresponding author with any questions on this robustness check and note that readers holding a Kroll license can reproduce this robustness check from the methodology below.

Importantly, CoC estimates can differ due to differences in i) key assumptions like the risk-free rate (RF), ii) time of the data, and iii) methodology and resulting values of CoC components. For instance, a CoC estimate that is two years older than our Damodaran-based estimate may easily differ due to shifts in interest rate levels and sectoral risk, but this would not indicate biases or issues with the Damodaran data. The same applies for CoC estimates that use a different RF assumption (e.g., one based on post-energy-crisis interest levels) or a more aggressive global equity risk premium (ERP). Therefore, we harmonize Kroll and Damodaran data regarding key assumptions and time of the data. Regarding the latter, our main estimates use the 5 January 2025 update of the Damodaran database. For maximum consistency, we use the 31 December 2024 update of the Kroll CoC Navigator for all financial indicators. To harmonize assumptions, we use the same ERP and RF estimates (see Methods) and our main Damodaran-based country-level tax rates (τ) but replace Damodaran values with Kroll inputs for the sector-specific debt share (DS), debt premium (DP) and betas, as well as the CRP.

Matching climate-relevant sectors to Kroll industries The Kroll CoC Navigator reports sector-specific CoC components with a global geographic coverage (i.e., based on a global sample of companies by industry group). Industry groups are identified via Global Industry Classification Standard (GICS) codes. We identify the best match for each of our climate-relevant sectors through desk research, as done for the Damodaran database's industry groups (see Methods) and show results in Supplementary Table S4.

Overall, the matched industries are very similar to the matched Damodaran industry groups (see Supplementary Table S1), as the 94 Damodaran industry groups are based on Value Line industry groupings (https://pages.stern.nyu.edu/~adamodar/pdfiles/blog/DataUpdate1for2025.pdf), which are not identical with GICS codes but seem to overlap to a large extent. In some cases, the additional granularity of GICS codes allows for a slightly higher precision. For instance, while the Damodaran industry groups matches most hydrogen producers to "Chemical (Specialty)", the GICS codes allow us to further differentiate between "15101050 - Specialty Chemicals" and "15101040 - Industrial Gases" (which includes hydrogen producers).

Equity and debt shares (DS) Equity and debt shares for each industry (i.e., $1 - DS_a$ and DS_a in Equation 1 of the main manuscript) based on a global sample are directly provided by Kroll.

Betas (β) Kroll provides industry-specific levered and unlevered beta estimates for different approaches, including the raw OLS beta (the beta used by Damodaran) and a Vasicek-adjusted beta that shrinks the raw OLS beta toward a pre-specified industry prior following ref.[36]. Here, we use Vasicek-adjusted betas, noting that the difference to the respective raw OLS betas for our industries is negligible (+/- 0.02 or less).

[1] Like the Damodaran database, Kroll does not provide country-differentiated debt shares, betas or debt premiums, and they provide market-specific equity risk premiums only for a subset of high-income markets.

	IMAGE sector	CoC Navigator industry (GICS)	Comment
1	Electricity	551010 Electric Utilities	*Note: Some example companies are also listed as "551050 – Independent Power and Renewable Electricity Producers".*
2	Electricity networks	551010 Electric Utilities	*Note: Some example companies are also listed as "551030 Multi-Utilities"*
3	Centralized heat generation	551030 Multi-Utilities	*Note: Some companies are also listed as "551010 Electric Utilities" or as "551050 Independent Power and Renewable Electricity Producers"*
4	Hydrogen production	15101040 – Industrial Gases	*Note: Some example companies are also listed as "15101020 – Diversified Chemicals", "15101030 – Fertilizers & Agricultural Chemicals", or "15101010 – Commodity Chemicals"*
5	Biomass production	10102030 – Oil & Gas Refining & Marketing	*Note: Some example companies are also listed as "30202010 – Agricultural Products & Services", "15101050 – Specialty Chemicals" or "551050 Independent Power and Renewable Electricity Producers"*
6	Cement	151020 Construction Materials	
7	Food processing	302020 Food Products	*Note: One example company is listed as "303020 - Personal Care Products"*
8	Iron and steel	15104050 - Steel	
9	Pulp and paper	15105020 – Paper Products	*Note: One example company is listed as "15103020 - Paper & Plastic Packaging Products & Materials"*

Table S4. Matching of climate-relevant technologies/subsectors to the GICS code used by the Kroll CoC Navigator.

Estimated betas are levered, that is, they also include the risk added by indebtedness and tax shield considerations. The Damodaran database provides levered betas, as well as unlevered betas using the commonly used Hamada formula:[4, 37]:

$$\beta_{a,unlevered} = \frac{\beta_a}{1+(1-\tau_i)\frac{DS_a}{1-DS_a}} \tag{S2}$$

Using unlevered betas then allows database users to re-lever these estimates using different assumptions regarding DS_a and τ_i. As stated in our Methods section, we use the Hamada formula for relevering, which ensures that the tax rates implicit in our betas match the tax rate of the country/region in question used for the cost of debt's tax shield. Note that we use globally uniform equity and debt shares by industry, so in our case, relevering betas does not adjust for between-region differences in industry equity shares, only for tax rate differences.

By contrast, the Kroll CoC Navigator uses the alternative Harris-Pringle formula[38], which accounts for differences in debt share and a so-called debt beta β_{debt}, for which the returns of publicly traded debt, rather than equity, is regressed on the overall market[23]:

$$\beta_{a,unlevered} = \frac{\beta_a + \beta_{a,debt}\frac{DS_a}{1-DS_a}}{1+\frac{DS_a}{1-DS_a}} \tag{S3}$$

Conceptually, a key difference between the two approaches is that the Hamada formula assumes that the tax shield has the same risk as debt, while the Harris-Pringle approach assumes that the realization of tax deductions is riskier.[2]

Equation S3 does not feature the corporate tax rate and instead only uses terms we assume to be uniform for a given industry (betas and the debt share). Therefore, there is no conceptual reason here to re-lever unlevered betas, as this would not account for any between-country heterogeneity — unlike for the Damodaran betas, where we re-lever to account for tax rate heterogeneity. To avoid unnecessary computations and any imprecision they might entail, we, therefore, take levered betas directly from the Kroll database.[3]

[2]For more details on the underlying theories of each approach and the appropriateness in different use cases, we refer readers to Chapter 12 of ref.[23].

[3]Note that re-levering unlevered Kroll betas based on the Hamada formula would mean that Equation S3 is used for unlevering and Equation S2 for re-levering, which would introduce a systematic bias in the relevered betas.

Country risk premiums (CRP^D) The CoC Navigator provides CRPs for investments denominated in USD based on the Country Yield Spread Model[22], which closely corresponds to the Damodaran approach of observing rating/yield spreads compared to risk-free markets and adding them to the cost of equity/debt. In the Country Yield Spread Model, countries are grouped into four tiers: Tier 1 (with $CRP_i = 0$) includes all AAA-rated countries and other countries deemed risk-free. Actual yields are used for countries, for which data is sufficiently available and otherwise based on sovereign credit ratings. Similar to the Damodaran database, countries without agency credit ratings are, where possible, assigned the expected spread for countries with a comparable country risk score by Euromoney (until 2023) and BMI (thereafter).[4]

Notably, the CoC Navigator also presents two alternative CRP approaches[22]: i) the Relative Volatility Model where the product of levered beta and ERP is further multiplied by a relative volatility factor that measures the foreign country's stock market volatility relative to the benchmark market (typically global or the US); ii) the Country Credit Rating Model based on ref.[39], which regresses equity returns on the natural logarithm of country credit ratings and uses the resulting model to predict country-specific cost of equity. Both approaches have higher data requirements and, therefore, come with a higher share of countries with missing values. In addition, the Country Yield Spread Model is listed with fewer conceptual shortcomings according to the database providers (see Exhibit 2.4 in ref.[22]) and matches the underlying assumptions of the Damodaran approach the closest. Therefore, we use Kroll values based on the Country Yield Spread Model, noting that this produces considerably higher CRP values for high-risk non-rated countries compared to the Country Credit Rating Model.

We calculate the same ten-year CRP average as for the Damodaran database.[5] Since the Damodaran database reports start-of-year values as of early January, we average end-of-year Kroll CRPs across 2015–2024, which corresponds to the start-of-year 2016–2025 values from Damodaran in our main approach.

Debt premiums (DP) The Kroll CoC Navigator does not directly report debt premiums but, instead, reports the pre-tax cost of debt (CoD) for a risk-free market. We convert pre-tax CoD to debt premiums by subtracting the Kroll risk-free rate as of 31 December 2024.

Comparing Damodaran- and Kroll-based CoC estimates ($WACC$) We compare the sector-specific estimates for debt premiums, equity shares and betas by the Damodaran and Kroll database, as well as the resulting CoC by sector for the US as a risk-free market, and summarize results here due to the data's proprietary nature. Due to near-identical betas and equity shares, differences in CoC are negligible (+/- 0.2% or less) for power generation and electricity grids, heat generation, pulp and paper, bioenergy production, and cement. By contrast, differences are somewhat higher for steel, hydrogen production and food processing but still amount to less than +/- 1% for each sector. As these sectors have fairly high equity shares, CoC differences are primarily driven by beta estimates and, for the steel sector, database differences in the equity share itself. Overall, however, the resulting CoC estimates are strongly correlated across sectors for the same region (rank correlation of r=0.92 and Pearson correlation of r=0.87 for the US).

Regarding the long-term averages of the country risk premium, correlations between the Damodaran- and Kroll-based values at the country level are even higher (Pearson correlation of r=0.90). However, we note that for high-risk markets, the Kroll database often indicates higher premiums, particularly for non-rated countries.

Due to CRP differences for higher-risk markets, the Kroll database leads to higher region-level CoC for most of our high-risk regions. However, due to the relatively low share of high-risk markets in GDP-weighted regional aggregates, the correlation at the IMAGE region level is even stronger compared to the country level. For the electricity sector, region-level CoC estimates based on the Damodaran and Kroll databases correlate with a Pearson correlation coefficient of r=0.94 and regions' rankings correlate near-perfectly (Spearman rank correlation of r=0.97).

Implications for IMAGE runs Given the strong correlations in CoC estimates across sectors and IMAGE regions, one would expect that using the Kroll-based estimates instead would modify some numerical results but not alter our main conclusions. Indeed, we confirm that all of our main conclusions hold when using the Kroll-based estimates

[4]The Damodaran database uses PRS' International Country Risk Guide data.

[5]Consistent with the Damodaran database, we use CRP estimates for investments that are USD-denominated.

instead but cannot disclose the numerical results due to licensing restrictions. The key reason why results hold is that the general risk ranking of sectors and, particularly, countries remains robust across the different robustness checks. As country risks for the Kroll-based CoC estimates tend to be higher for high-risk regions particularly, the increase in global mitigation cost due to differentiating the CoC is somewhat larger than the +9% reported in our main manuscript.

Supplementary Note 3: Additional information on estimating the GDPpc-risk premium feedback

Data The country-level panel data set was collected from ref.[40] and the World Bank's WDI database[41]. For country risk premiums (CRPs), data cleaning was performed to discard duplicate values and, for some countries and years, additional (lower) estimates for the country as a center for offshore banking instead of domestic economic activities (e.g., the Cayman Islands). For the United Arab Emirates, values for individual emirates were discarded to ensure that we have only one country risk premium per ISO3 code and year. Summary statistics for the resulting sample are displayed in Supplementary Table S5. The observed country risk premiums range between 0% for AAA-rated countries and up to 18% for high-risk countries, such as Sudan, Venezuela, and Yemen. Log-transformed GDP per capita ranges between 6.9 and 11.9 (roughly 1,000 and 150,000 USD, respectively). Missing values for the GDP (per capita) and the population variables arise from the 2025 year (for which only start-of-year country risk premiums are observed) and from a few countries not included in the underlying World Bank WDI database, such as Venezuela.

Table S5. Summary statistics for the regression sample

	Mean	N	St.Dv.	Min	Max
year	2014.48	3412.00	7.15	2001.00	2025.00
crp_debt_defaultspread	0.03	3412.00	0.03	0.00	0.18
ln_gdp_pc	9.68	3031.00	1.03	6.90	11.86
gdp	930.06	3031.00	2728.54	0.63	33 597.90
pop	48.34	3159.00	165.90	0.03	1450.94

Motivation for weighting observations IMAGE regions can be viewed as GDP-weighted aggregates of individual countries, meaning that larger economies disproportionately drive region-level results. To ensure that our country-level regression produces estimates consistent with IMAGE regions and to avoid distortions by small economies with atypical GDPpc patterns, we weight countries by GDP across our different specifications (Supplementary Table S6). Importantly, this approach produces results that are almost identical to using population-based weights and that are consistent if we replicate our regression at the IMAGE region level by calculating GDP-weighted averages of GDPpc and CRP across countries (Supplementary Table S8). As weighting all countries equally produces a higher estimate for the impact of GDPpc on the CRP, we also run IMAGE using this higher estimate as a robustness check (see Supplementary Note 4).

Motivation for using fixed effects The extant literature has identified a wide range of country rating and CRP determinants, many of which are time-constant, such as the industrialized/non-industrialized status[42], geographical/economic/cultural proximity to the US[43], and whether or not the rating agency is based in the country in question[44]. Similarly, CRPs can spike in crisis years (Supplementary Fig. S6), posing a risk of omitted variable bias if the regression does not account for such common shocks across countries. To account for these potential sources of distortion, we include country and year fixed effects, as is increasingly common in the literature[45, 46, 47, 48].

Results and robustness checks Results for the regression model outlined in the Methods section are displayed in Supplementary Table S6, with standard errors two-way clustered by country and year. Consistent with the literature, we find that increases in log-transformed GDP per capita are significantly associated with decreases in the country risk premium, both before (col. 1) and after introducing fixed effects (col. 2). Controlling for last year's country risk premium reduces the initial effect considerably (col. 3), as countries' ratings in adjacent years are strongly correlated. When combined with fixed effects, this results in a point estimate of about 0.56 percentage points per one-unit increase in log-transformed GDP per capita (col. 4). We find no clear evidence for a non-linear effect (col. 5). Introducing country-specific time trends (col. 6–7), or altering the weighting of observations (col. 8–9), increases the point estimate or leaves it roughly unaltered. The same holds for discarding the (post-)pandemic years

from 2020 on (col. 10) or limiting the sample to BRICS countries only (col. 11), although these strong sample size reductions unsurprisingly increase the p-value. To be conservative, we opt for the main specification in col. 4 and use the estimate from the unweighted regression in col. 8 as a robustness check (see Supplementary Note 4).

Notably, studies predicting sovereign credit ratings typically identify a range of determinants, including government debt, inflation, unemployment, fiscal balance, current account balances, default history, and institutional quality (see Table 1 in ref.[43] for an overview). As these other determinants are not easily captured in IMAGE and the underlying SSP scenarios, our model abstracts from them and, by doing so, loads all effects of correlated determinants (net of fixed effects and, potentially, time trends) onto the GDP per capita variable as a catch-all predictor. However, we note that a 0.56 percentage point reduction in country risk per one-unit increase in log-transformed GDP per capita is consistent with effect sizes of rating upgrades of 1–2 notches reported by previous studies[49, 50], as a one-notch upgrade approximately yields a 0.3 percentage point reduction for investment grade-rated countries, with substantially higher spread effects for sub-investment-grade countries (see the default spreads by rating category reported by ref.[40]).

Motivation for not using specifications involving time trends We do not use the much starker estimate based on the model involving time trends for several reasons. First, extant literature on country rating determinants does not deploy time trends (cf. the overview in ref.[43]). Second and more importantly, the resulting coefficients of -0.03 (or even -0.05) would predict CRP decreases of 3–5% (or even up to 9%) for important high-growth economies, such as China, India or Vietnam, over the past two decades that are hard to reconcile with actual decreases around 1% or less (Supplementary Fig. S5). Lastly, the fact that effect sizes vary strongly across different trend specifications (col. 6 and 7) and increases in the BIC potentially suggest overfitting issues.

Table S6. Regressing country risk premiums on log-transformed GDP per capita

Dependent Variable:	crp_debt_defaultspread										
	noFEs	+ FEs	+ lag Dep.Var.	main	+ quadratic	+ lin. trend	+ lin. & quadr. trend	unweighted	POP-weighted	noCovid	onlyBRICS
Model:	(1)	(2)	(3)	(4)	(5)	(6)	(7)	(8)	(9)	(10)	(11)
Variables											
Constant	0.1464***		0.0152$^{.}$								
	(0.0355)		(0.0073)								
l(ln_gdp_pc,1)	-0.0132***	-0.0137*	-0.0014$^{.}$	-0.0056*	0.0187	-0.0308*	-0.0506***	-0.0131***	-0.0057*	-0.0065$^{.}$	-0.0071
	(0.0033)	(0.0050)	(0.0007)	(0.0024)	(0.0221)	(0.0132)	(0.0134)	(0.0033)	(0.0022)	(0.0035)	(0.0090)
l(crp_debt_defaultspread,1)			0.9028***	0.6184***	0.6162***	0.4169***	0.2338$^{.}$	0.6399***	0.6489***	0.6112***	0.4443*
			(0.0509)	(0.0689)	(0.0694)	(0.0954)	(0.1297)	(0.0624)	(0.0605)	(0.1114)	(0.1357)
l(ln_gdp_pc,1) square					-0.0014						
					(0.0012)						
Fixed-effects											
iso3		Yes		Yes	Yes	Yes	Yes	Yes	Yes	Yes	Yes
year		Yes		Yes	Yes	Yes	Yes	Yes	Yes	Yes	Yes
Varying Slopes											
year (iso3)						Yes	Yes				
year$\hat{2}$ (iso3)							Yes				
Fit statistics											
Observations	3,023	3,023	3,023	3,023	3,023	3,023	3,023	3,023	3,023	2,054	120
R^2	0.29294	0.83676	0.85404	0.90346	0.90371	0.92069	0.93384	0.90528	0.92144	0.91626	0.76073
Within R^2		0.06081		0.44458	0.44601	0.27064	0.12708	0.46674	0.46901	0.48251	0.26572
BIC	-13,113.1	-16,060.6	-17,928.1	-17,657.6	-17,657.2	-16,860.6	-16,020.3	-17,935.2	-17,809.4	-12,233.9	-705.51

Clustered (iso3 & year) standard-errors in parentheses
*Signif. Codes: ***: 0.001, **: 0.01, *: 0.05, .: 0.1*

Table S7. Adding further lags of the dependent variable

Dependent Variable:	crp_debt_defaultspread				
Model:	(1)	(2)	(3)	(4)	(5)
Variables					
l(ln_gdp_pc,1)	-0.0056*	-0.0046*	-0.0045*	-0.0057*	-0.0056*
	(0.0024)	(0.0020)	(0.0021)	(0.0024)	(0.0024)
l(crp_debt_defaultspread,1)	0.6184***	0.6443***	0.6322***	0.6370***	0.6375***
	(0.0689)	(0.0592)	(0.0578)	(0.0643)	(0.0735)
l(crp_debt_defaultspread,2)		0.0173	0.0110	0.0064	0.0011
		(0.0357)	(0.0313)	(0.0337)	(0.0467)
l(crp_debt_defaultspread,3)			-0.0080	-0.0105	-0.0255
			(0.0469)	(0.0952)	(0.1092)
l(crp_debt_defaultspread,4)				0.0171	0.0559
				(0.0915)	(0.1358)
l(crp_debt_defaultspread,5)					-0.0424
					(0.0415)
Fixed-effects					
iso3	Yes	Yes	Yes	Yes	Yes
year	Yes	Yes	Yes	Yes	Yes
Fit statistics					
Observations	3,023	2,856	2,691	2,527	2,363
R^2	0.90346	0.90854	0.91292	0.91401	0.91420
Within R^2	0.44458	0.46687	0.46593	0.46063	0.44870
BIC	-17,657.6	-16,569.4	-15,771.9	-14,832.2	-13,877.2

Clustered (iso3 & year) standard-errors in parentheses
*Signif. Codes: ***: 0.001, **: 0.01, *: 0.05, .: 0.1*

Table S8. Regressing country risk premiums on log-transformed GDP per capita for GDP-weighted IMAGE region aggregates

Dependent Variable:	crp_debt_defaultspread			
	naive	+ FEs	main	+ lin. trend
Model:	(1)	(2)	(3)	(4)
Variables				
Constant	0.2042***			
	(0.0289)			
l(ln_gdp_pc,1)	-0.0185***	-0.0297***	-0.0070**	-0.0049
	(0.0028)	(0.0035)	(0.0022)	(0.0038)
l(crp_debt_defaultspread,1)			0.7407***	0.5368***
			(0.0644)	(0.0902)
Fixed-effects				
iso3		Yes	Yes	Yes
year		Yes	Yes	Yes
Varying Slopes				
year (iso3)				Yes
Fit statistics				
Observations	466	466	466	466
R^2	0.39019	0.81780	0.90918	0.92351
Within R^2		0.34779	0.67489	0.31168
BIC	-2,392.4	-2,691.2	-3,009.5	-2,954.3

Clustered (iso3 & year) standard-errors in parentheses
*Signif. Codes: ***: 0.001, **: 0.01, *: 0.05, .: 0.1*

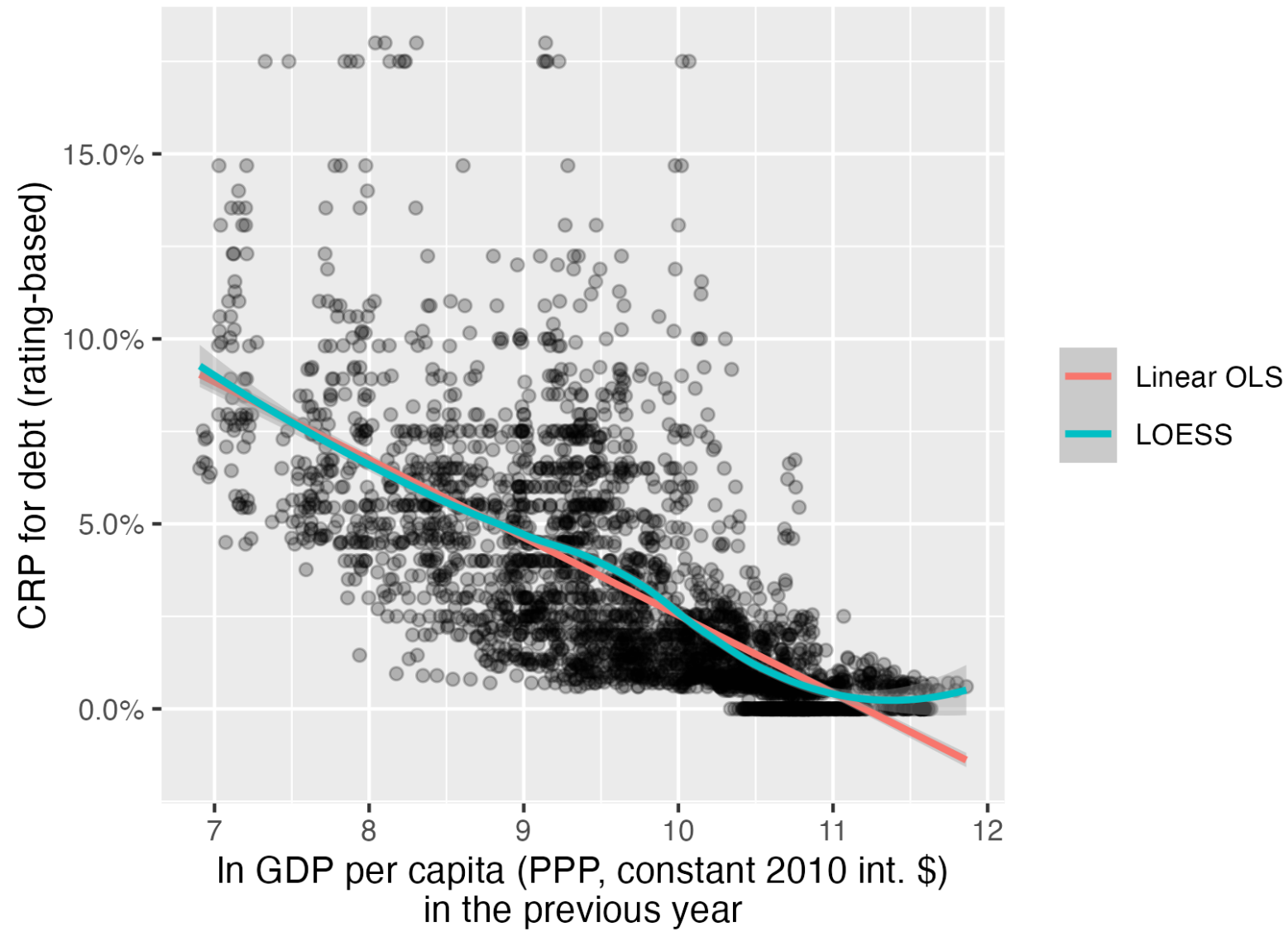

Figure S4. Scatter plot and simple OLS and LOESS regression lines (excl. fixed effects and time trends) for the link between country risk premiums (start-of-year) and GDP per capita. Note that the regression line shown here serves merely illustrative purposes and does not correspond exactly to the specification in Supplementary Table S6, col. 1, because the latter uses GDP weighting while the line shown here is unweighted. For the motivation to use GDP weighting, see Supplementary Note 3.

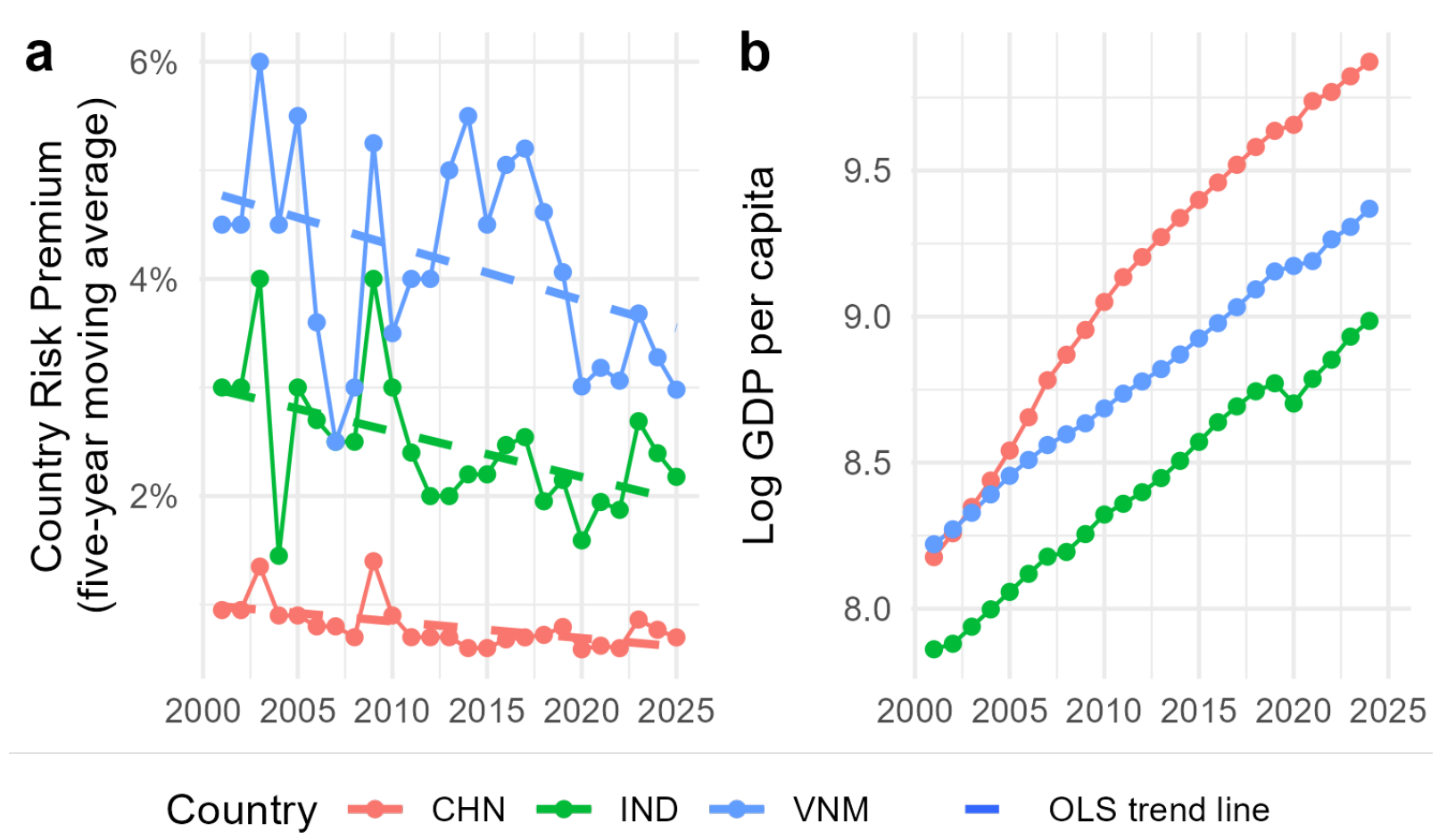

Figure S5. ln GDPpc and CRP evolution in high-growth markets over our sample period

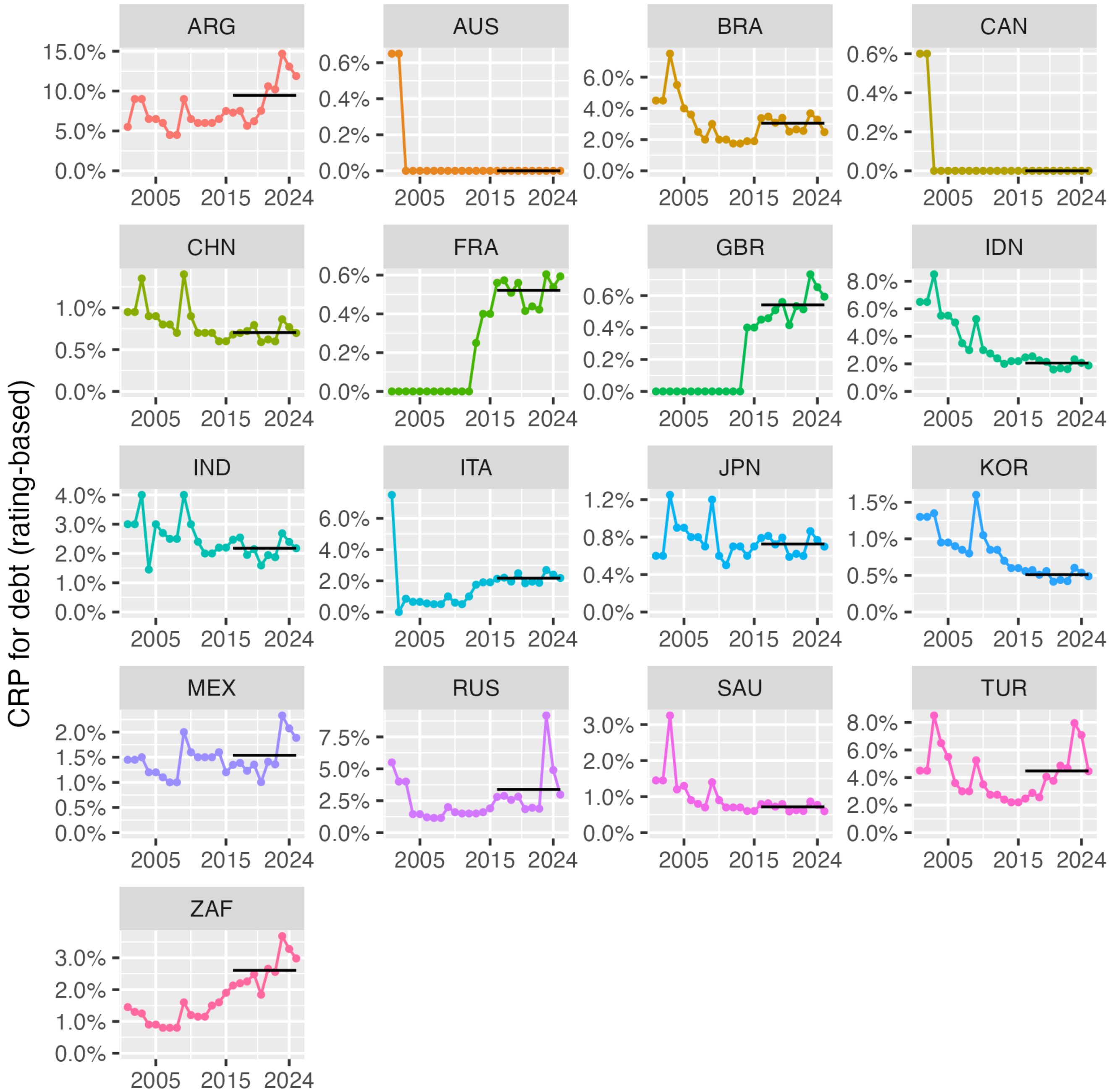

Figure S6. Rating-based country risk premiums for debt over time and the 2016–2025 mean used for our CoC estimates (black line), displayed for G20 countries (excluding Germany and the US, which have a consistent country risk premium of 0%)

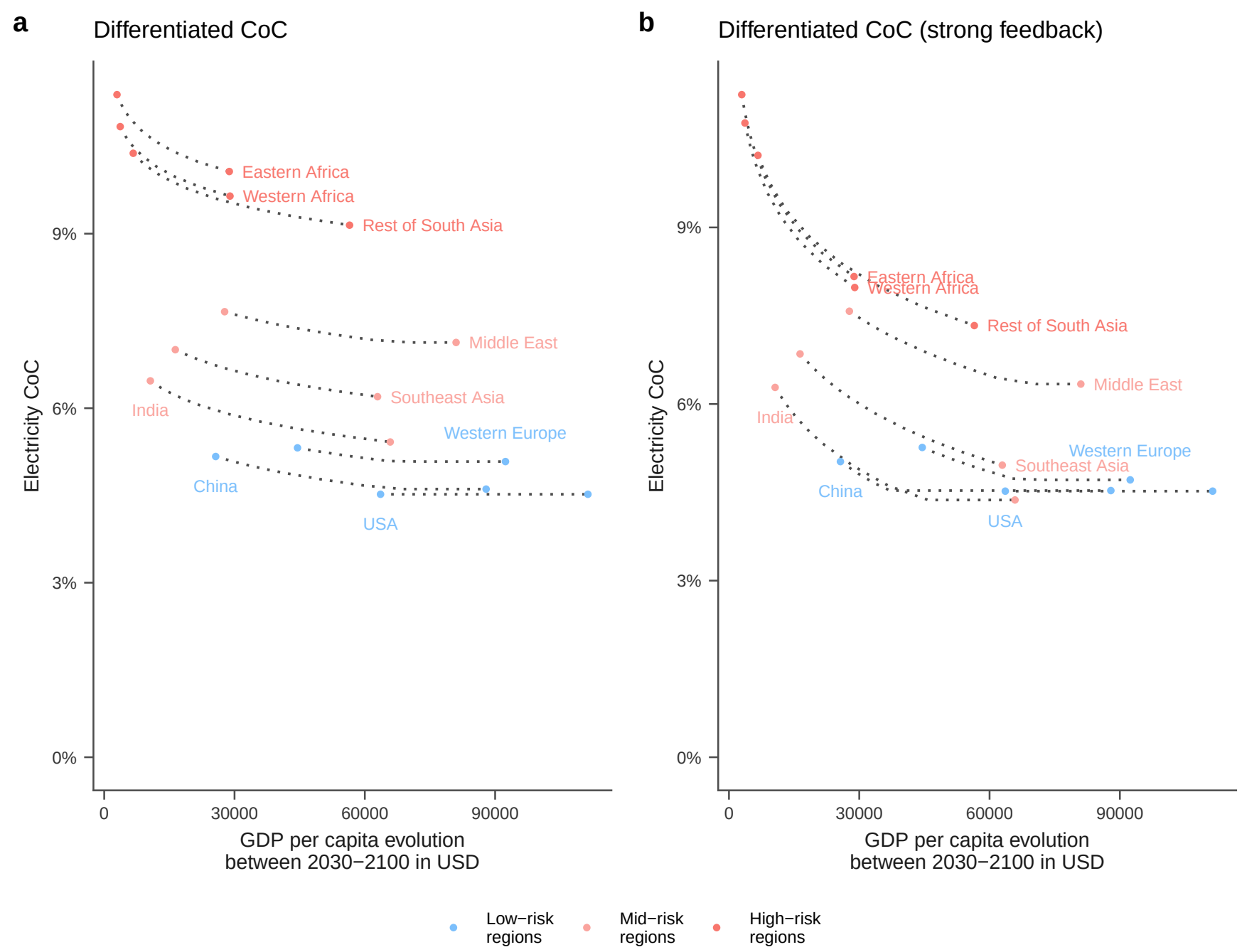

Figure S7. Robustness check: Panel d of Fig. 1 using the alternative estimate from the unweighted regression model in Supplementary Table S6

Supplementary Note 4: Key IMAGE results under different robustness check scenarios

Scenarios used for robustness check In this section, we display our key results across the following range of (robustness check) scenarios:

- *Uniform low-risk CoC*: Applying a time-constant 5% uniform CoC to all sectors in our scope (Supplementary Table S2)
- *Differentiated CoC (power+heat only)*: Implementing our region- and sector-differentiated CoC estimates only for electricity (generation and grids) and heat generation and using the uniform 5% for all other sectors in our scope
- *Differentiated CoC*: Our main scenario
- *Differentiated CoC (strong feedback)*: Same as our main scenario but using the stronger -0.0131 coefficient for the GDPpc-CRP feedback (see Supplementary Note 3)
- *Differentiated CoC (regional betas)*: Replacing the Damodaran-based global beta estimates with region-differentiated ones (see Supplementary Note 1)
- *Differentiated CoC (10-year betas)*: Replacing the latest Damodaran-based beta estimates from our main approach with averages over the past ten years (see Supplementary Note 1)
- *Uniform high-risk CoC*: Applying a time-constant 10% uniform CoC to all sectors in our scope (see Supplementary Note 1)

Mitigation cost results (Supplementary Fig. S8): Results for the *Differentiated CoC (power+heat only)* illustrate that the increase in mitigation costs between *Uniform low-risk CoC* and the *Differentiated CoC* is primarily driven by energy price shifts and robust across different scenarios with differentiated CoC. By contrast, assuming a high uniform CoC of 10% would yield considerably higher cumulative mitigation costs compared to *Differentiated CoC*.

In addition to the undiscounted cumulative value, we also present results using a time-varying Ramsey-style discount rate $\rho + \eta g_{r,t}$ where $g_{r,t}$ is the GDPpc growth rate of IMAGE region r in year t and discount all costs to 2025 (i.e., the base year of our analysis). For the rate of pure time preference ρ and the elasticity of marginal utility of consumption η, we use IMAGE's default settings of $\rho = 0.1\%$ and $\eta = 1$. Discounting mitigation costs reduces the magnitude of values but leaves the conclusions from the undiscounted values intact.

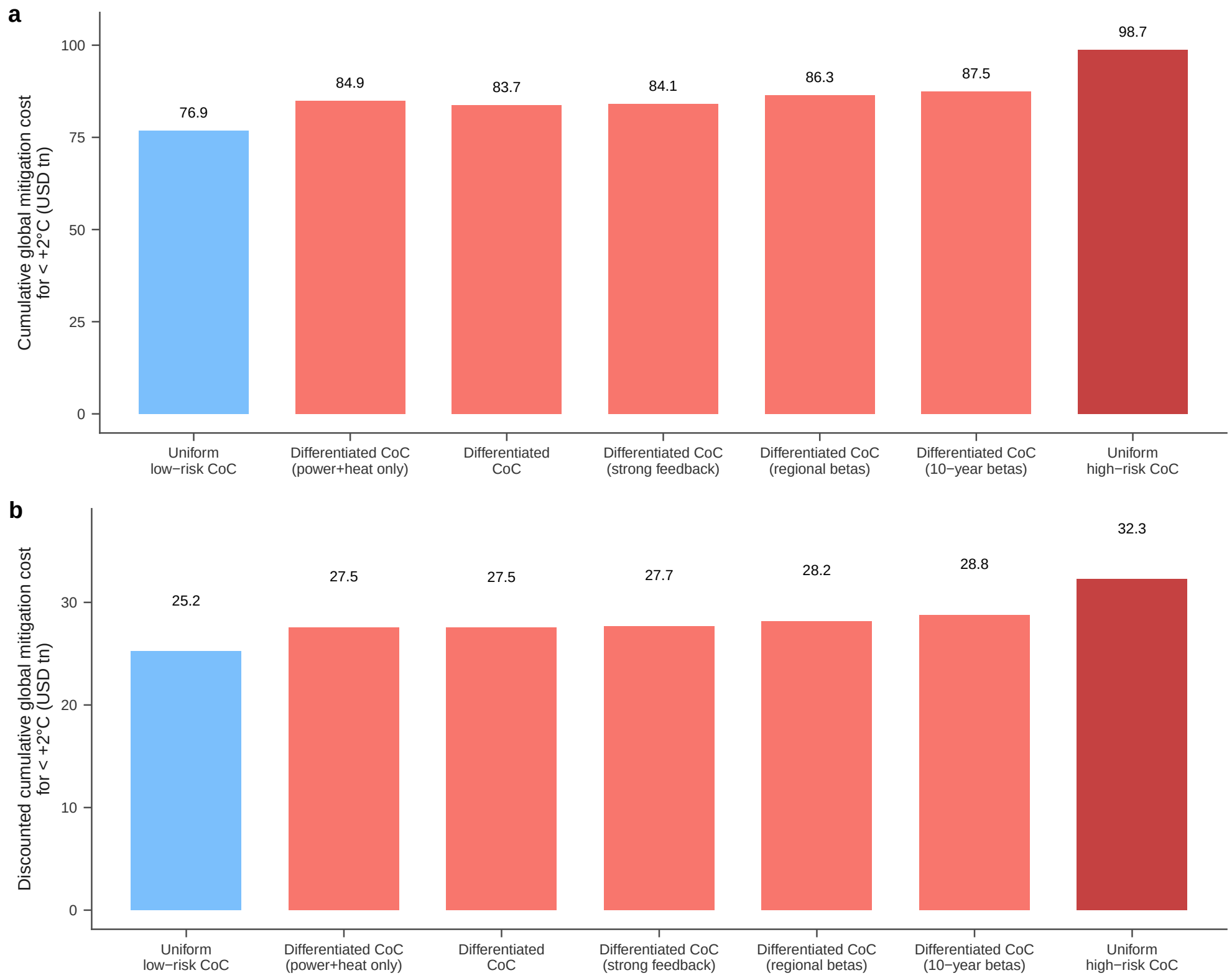

Figure S8. Robustness check: Increase in cumulative mitigation cost (Fig. 3a)

Cumulative CO_2 emission results (Supplementary Fig. S9): Emission increases and their sectoral composition are broadly similar across scenarios but much more pronounced in the *Uniform high-risk CoC* scenario and somewhat less pronounced for the stronger GDPpc feedback. In response to the climate policy reaction through more stringent carbon pricing, all well below +2°C scenarios show emission reductions in the electricity sector with contrasting increases in industry, transportation, and other sectors.

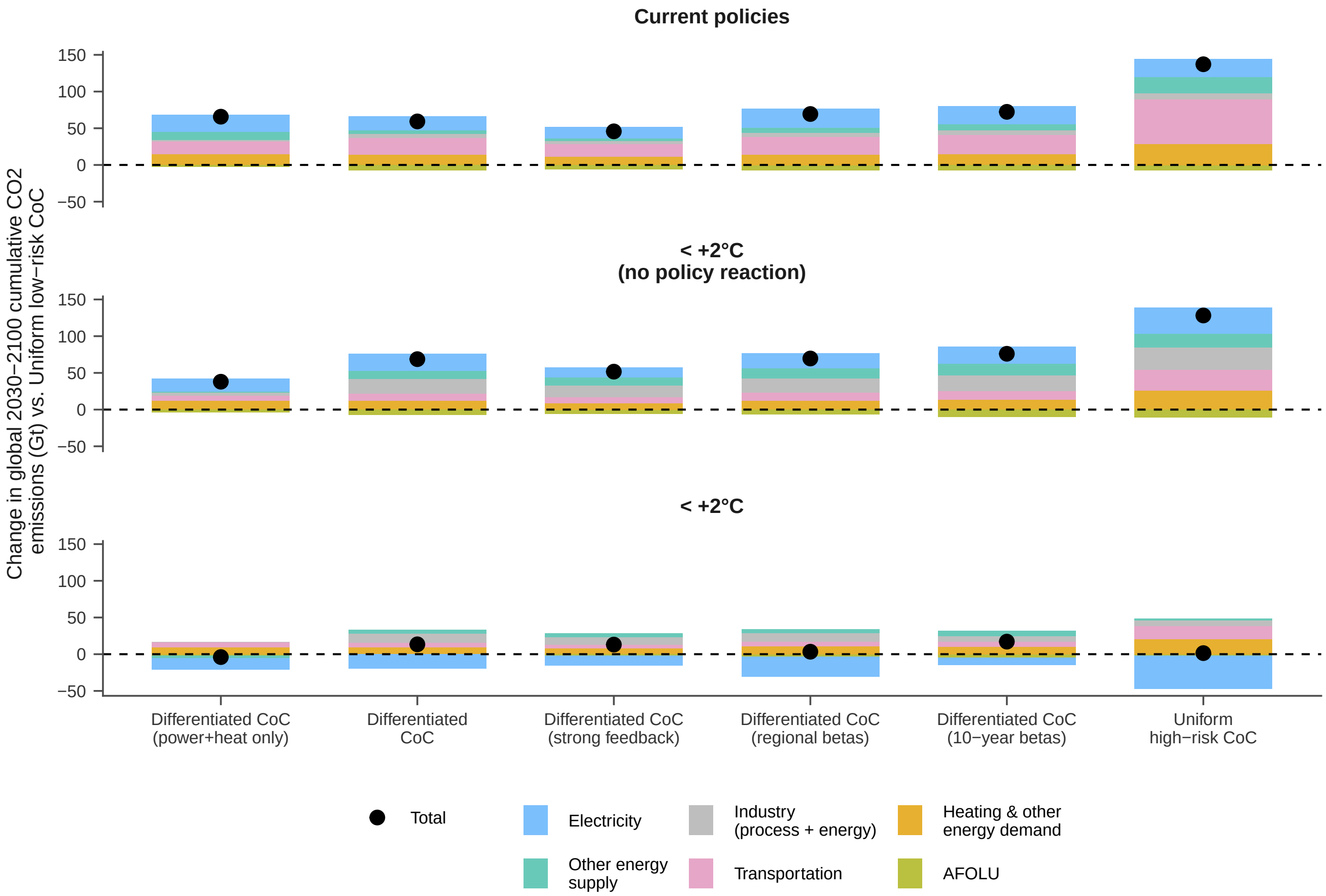

Figure S9. Robustness check: Increase in cumulative CO_2 emissions (Fig. 3b)

Price shifts for electricity and hydrogen (Supplementary Fig. S10): Compared to the *Uniform low-risk CoC*, prices increase across all scenarios, except in the *Differentiated CoC (power+heat only)*, which features unchanged or even slightly lower hydrogen costs at the global level but not for high-risk regions. Across all scenarios with CoC differentiation, price increases due to CoC differentiation are more pronounced in the High-risk regions than the global average.

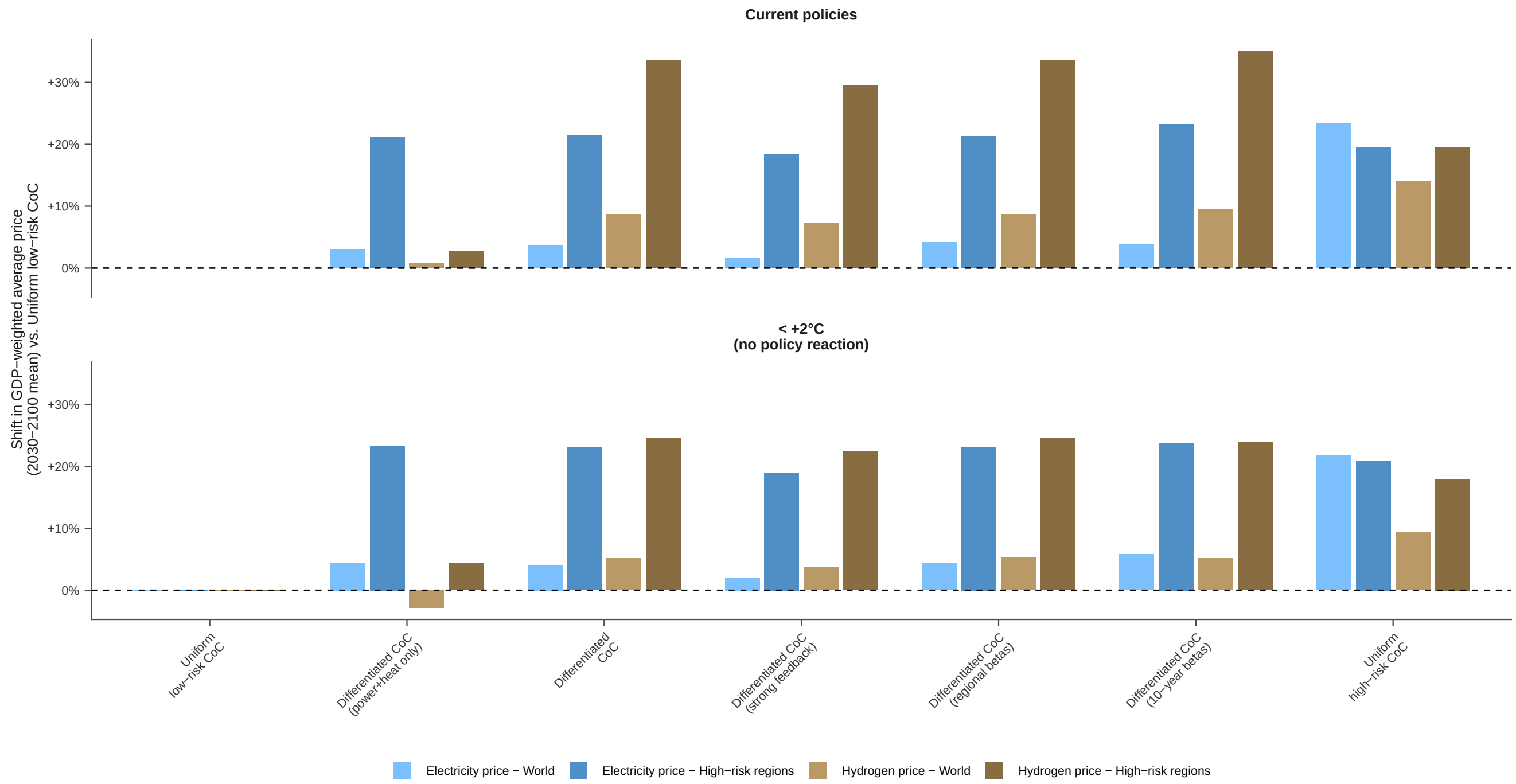

Figure S10. Robustness check: Electricity and hydrogen cost shifts compared to the *Uniform low-risk CoC* scenario (Fig. 4b)

Hydrogen production shifts (Supplementary Fig. S11): Across all scenarios with differentiated CoC for the hydrogen sector, hydrogen production is considerably lower compared to the *Uniform low-risk CoC*. The intermediate *Differentiated CoC (power + heat only)* scenario reveals that this effect is driven by accounting for sectoral risks of the hydrogen sector itself. The *Uniform high-risk CoC* includes somewhat higher hydrogen production volumes because for many mid- and high-risk regions, the uniform 10% assumption is lower than their empirical hydrogen CoC estimate (Supplementary Table S9).

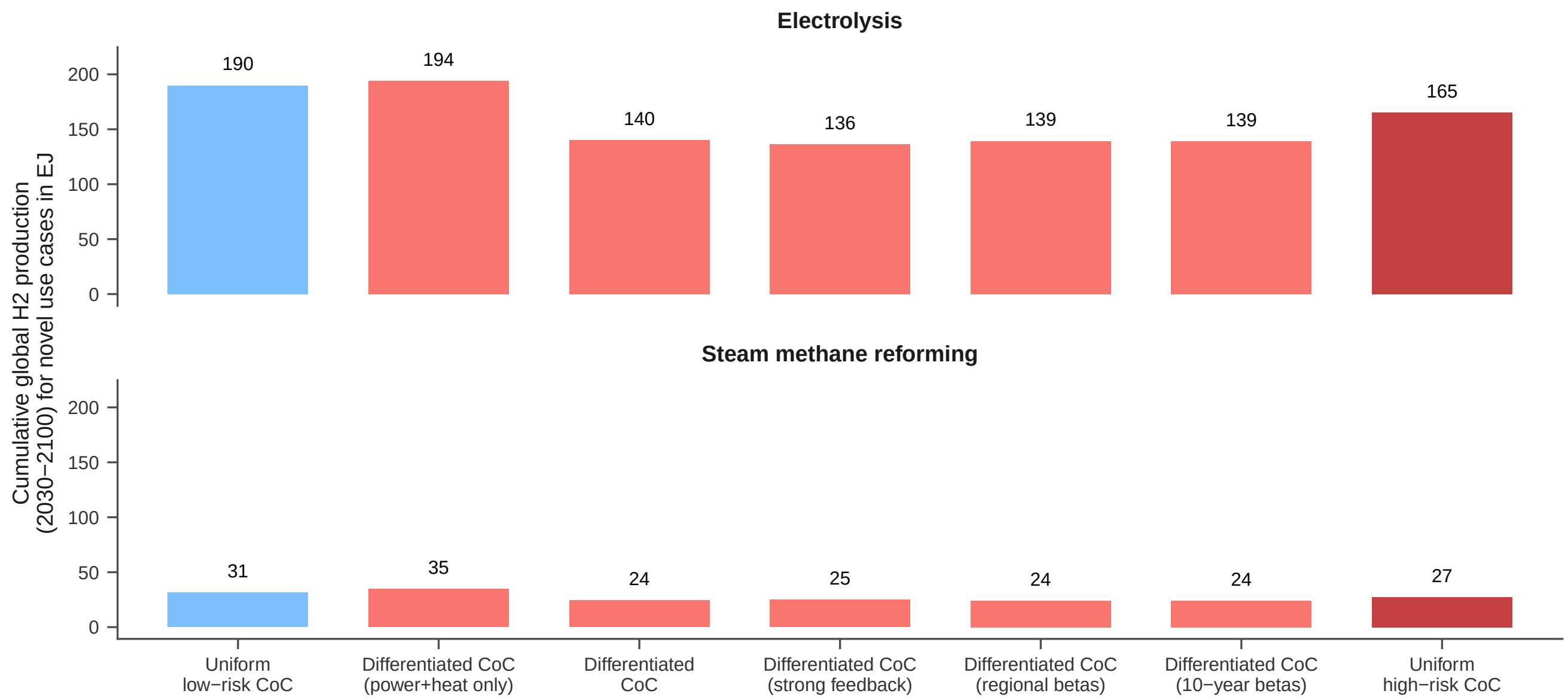

Figure S11. Robustness check: Hydrogen production shifts (Fig. 4c)

Industry tech shares (Supplementary Fig. S12) and carbon capture (Supplementary Fig. S13): Global market shares of more efficient technologies and technologies involving CCS decrease in similar fashion for the different robustness check scenarios using *Differentiated CoC*, albeit somewhat less for the stronger GDPpc feedback. The intermediate results for the *Differentiated CoC (power+heat only)* scenario illustrate that this effect is entirely driven by industry sector CoC assumptions, not by shifts in electricity or heat prices. The same holds for the decrease in cumulative industrial carbon capture.

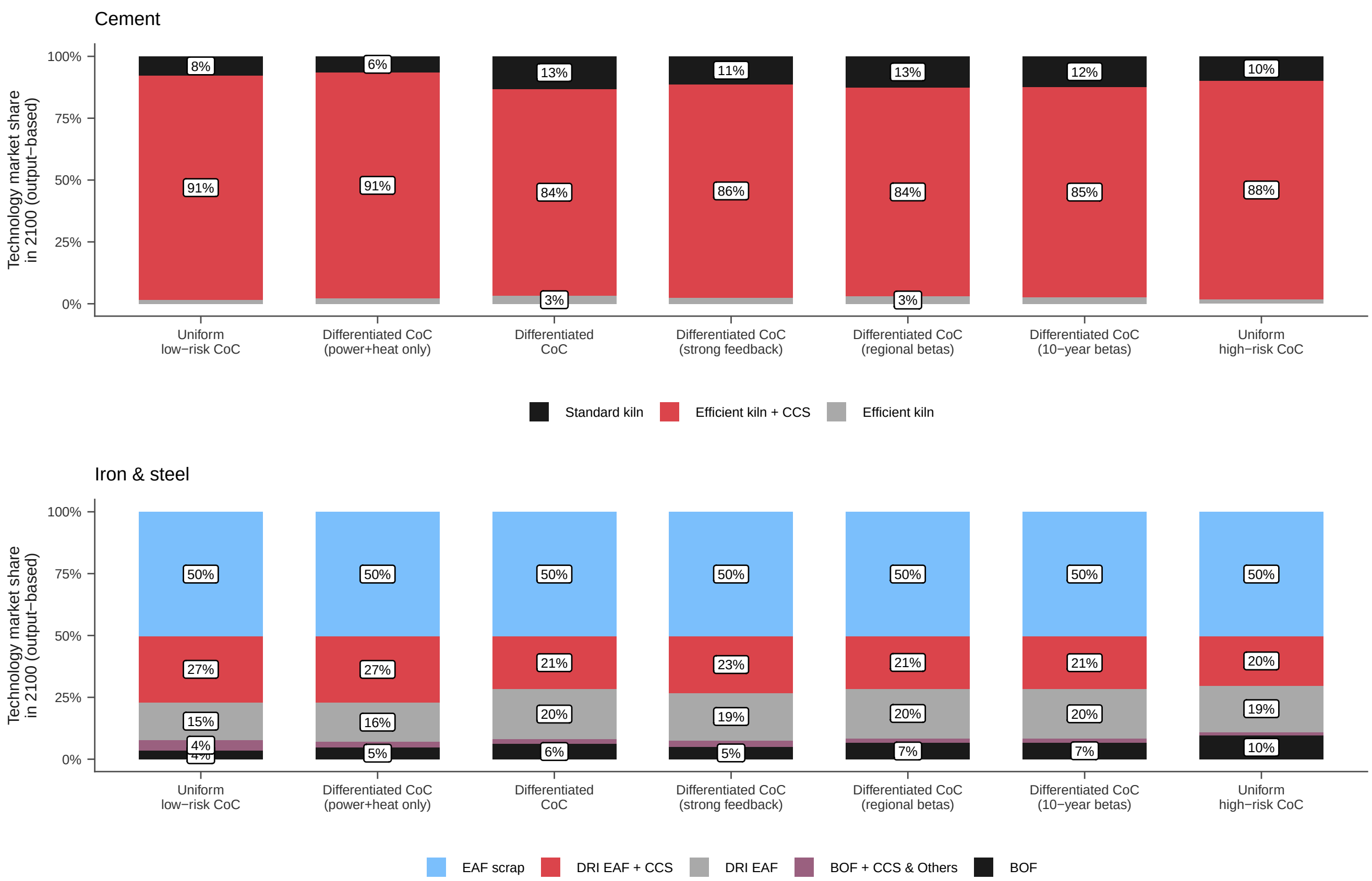

Figure S12. Robustness check: 2100 industrial tech share shifts (Fig. 5a-b)

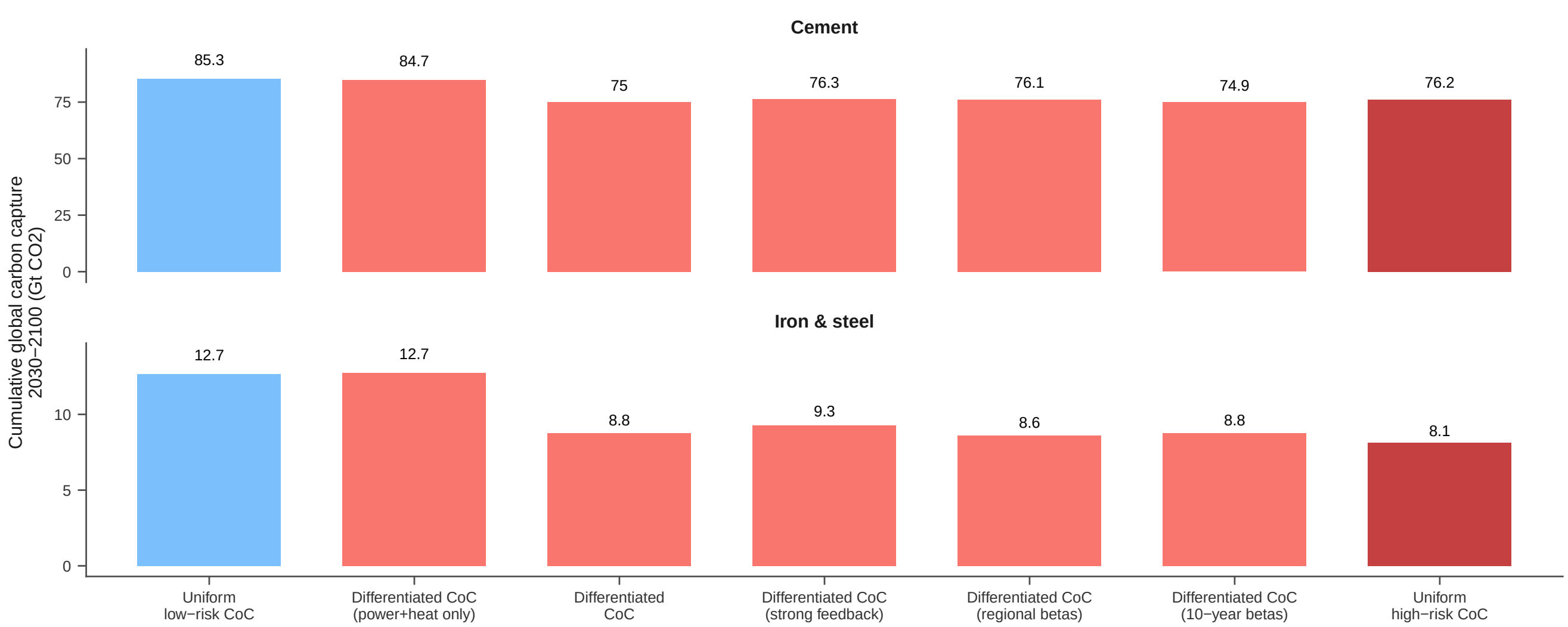

Figure S13. Robustness check: Cumulative industrial carbon capture (Fig. 5c)

Results for energy and industry sectors including the climate policy reaction (Supplementary Fig. S14-S15): Fig. 4 and 5 in the main manuscript visualize the pure effect of CoC differentiation by excluding IMAGE's climate policy reaction through higher carbon pricing. The main motivation is to avoid falsely attributing model outcomes to CoC changes that are, in fact, caused by carbon pricing changes (and any spillover and interaction effects these

pricing changes may trigger in return). For the sake of completeness, here we present the same figures using the well below 2°C scenario with the climate policy reaction instead. As the figures illustrate, all our main conclusions would remain unchanged if we were to use the scenario including the climate policy reaction instead.

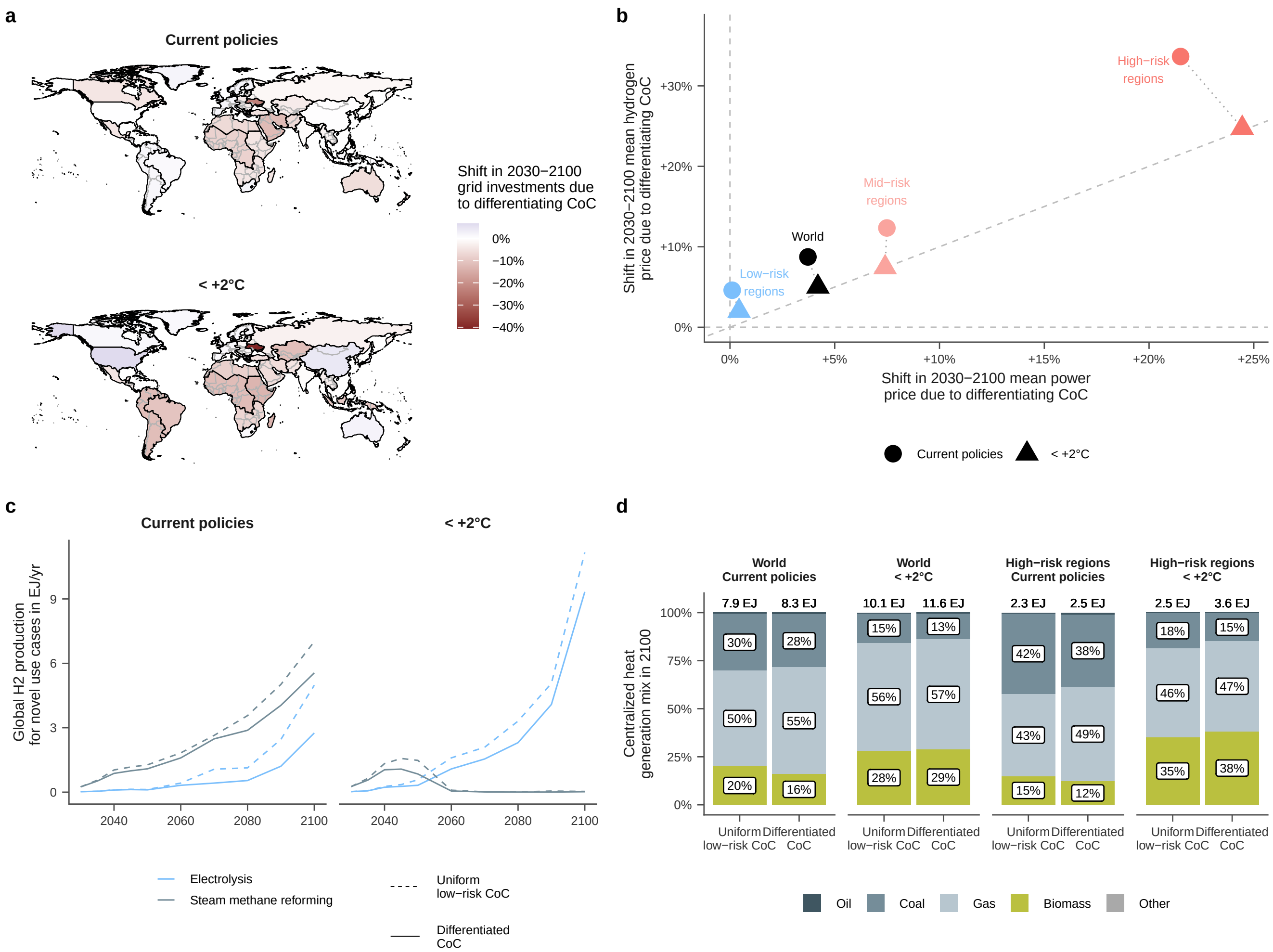

Figure S14. Fig. 4 when using the well below +2°C scenario with climate policy reaction instead of the one without. Map data from Natural Earth via the rnaturalearth R package[51].

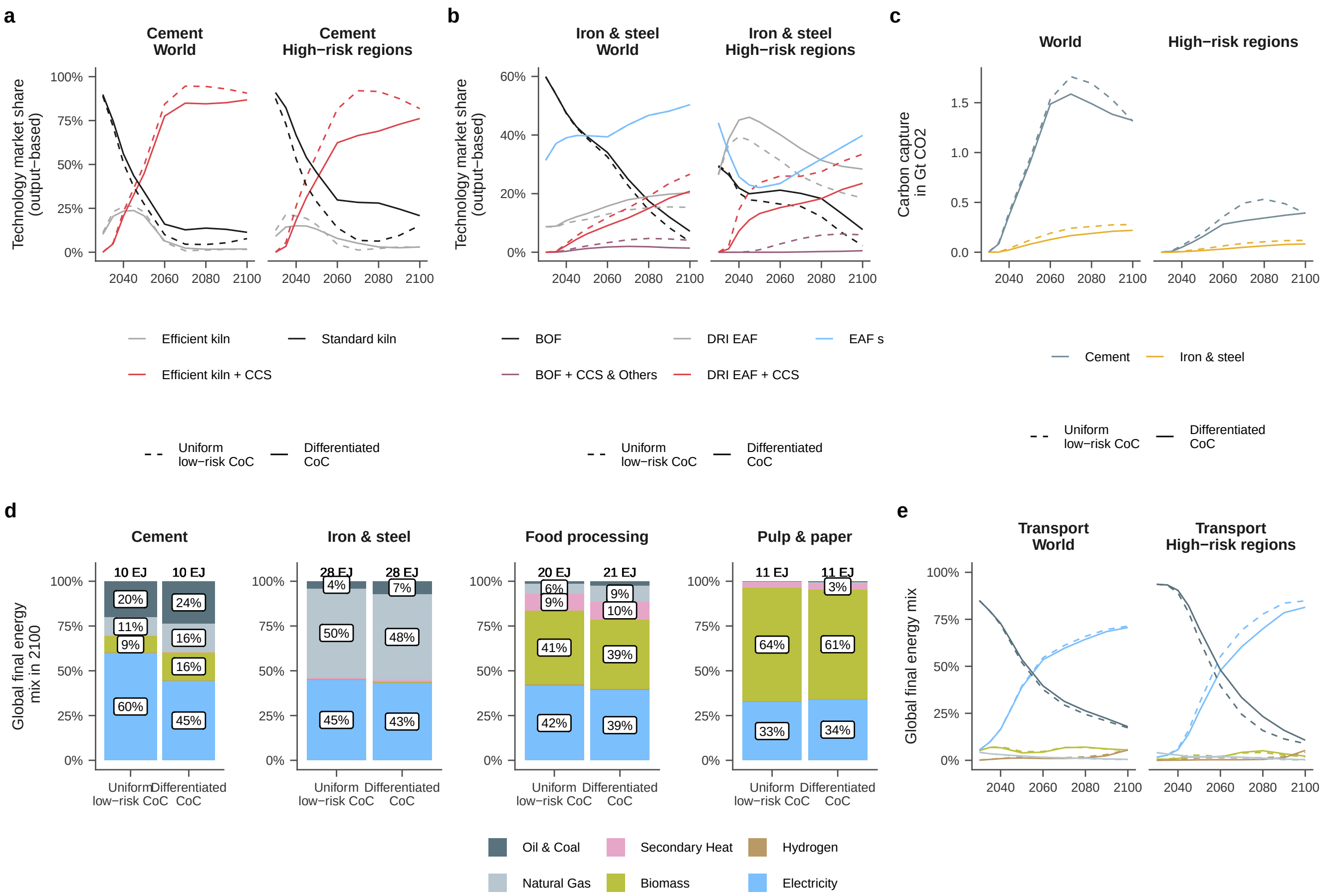

Figure S15. Fig. 5 when using the well below +2°C scenario with climate policy reaction instead of the one without.

Supplementary Note 5: Additional information on the IMAGE model

Investment decisions, foresight and expectations in IMAGE In IMAGE, several sectors, including the industrial sectors, electricity, hydrogen and heat generation, take future fuel prices and carbon prices up to ten years ahead into account for investment decisions. For these sectors, future fossil fuel price expectations are determined by trends in past simulation years, while carbon price expectations assume perfect foresight. By contrast, other sectors, such as transportation or buildings, assume more myopic investment decisions and do not take future fuel or carbon prices into account.

In TIMER, the energy system model used by IMAGE, the supply of energy carriers is set to follow the demand of energy. Investment decisions are often made based on relative costs. As a result, revenues do not have to cover the expenses of a sector by definition. Some sectors, like the electricity sector, do include the option for early retirement of existing capacity when the variable costs of existing capacity get more expensive compared to the full cost of installing new capacity.

In the steel, cement, paper and food processing sectors, market shares of new investments are simulated using multinomial logit functions, with the lowest-cost option receiving the largest share in investments. A logit factor determines the sensitivity of market allocation to price differences. The share of energy carriers for each production technology is first determined to simulate production costs of each technology, before allocating technology shares based on these costs. Concerning the time horizon of investments, all plants are assumed to be used for their entire technical lifetime of 40 years[52].

Final demands in IMAGE Demand for steel, cement, and paper in IMAGE is estimated based on historical relationships between the respective demand and GDP, which is then applied to future GDP levels as per the underlying Shared Socio-economic Pathway. Therefore, these demand volumes are exogenous and do not respond to prices or CoC assumptions. For most appliances in the residential and service sectors, there is a (relatively weak) relationship between electricity prices and service demand. For space heating, increasing prices can result in increased use of insulation and therefore impact heating demand.[53] In the transport sector, energy prices affect transport mode and vehicle shares. For passenger transport, demand for transport (passenger kilometers) is based on a travel money budget that is modeled as a fixed share of GDP.[54] Energy prices, therefore, determine how this budget can be spent. However, even for sectors where demand volumes are exogenous, the technology choices, trade routes, and energy carrier usage to meet demand are endogenous and thus respond to changes in energy and carbon prices, as well as the CoC.

Carbon pricing in IMAGE When determining the carbon prices for the mitigation scenarios in IMAGE, the model allows full emission trading between regions, resulting in a globally uniform carbon price trajectory over time, which is illustrated in Supplementary Fig. S16 for the *Uniform low-risk CoC* and the *Differentiated CoC* scenarios. The carbon prices in the current policy scenarios are region-specific and based on actually implemented carbon prices or carbon prices that would be required to meet the current policy goals (for details, see ref.[55]).

Mitigation costs in IMAGE Climate change mitigation in our IMAGE scenarios is enforced using a carbon price. Mitigation costs in IMAGE are defined as the area under the marginal abatement curve (MAC). This area represents the total expenditure (or net savings) of reducing greenhouse gases relative to the relevant baseline scenario, in which no carbon price is applied. Existing policies other than the carbon price (e.g., renewable energy targets) are included in the related baseline and are therefore not included in the mitigation costs. Therefore, the mitigation costs represent additional system costs that arise from reducing greenhouse gases beyond the trends already included in the baseline scenario. Notably, other IAMs may use different mitigation cost definitions, such as reductions in GDP.

For IMAGE applications, MAC curves are implemented mainly in the climate policy module but are derived from other parts of the framework, in particular the energy module and the land use module. The underlying models simulate the deployment of mitigation options, such as CCS, bioenergy, wind and solar, energy efficiency and other technological improvements, under varying carbon prices or policy assumptions; from these simulations, IMAGE extracts abatement-cost pairs and calibrates MAC functions per greenhouse gas, region and sector. Then, IMAGE uses these (dynamic) MAC curves for different regions, gases (all Kyoto GHGs) to translate a given carbon price (or

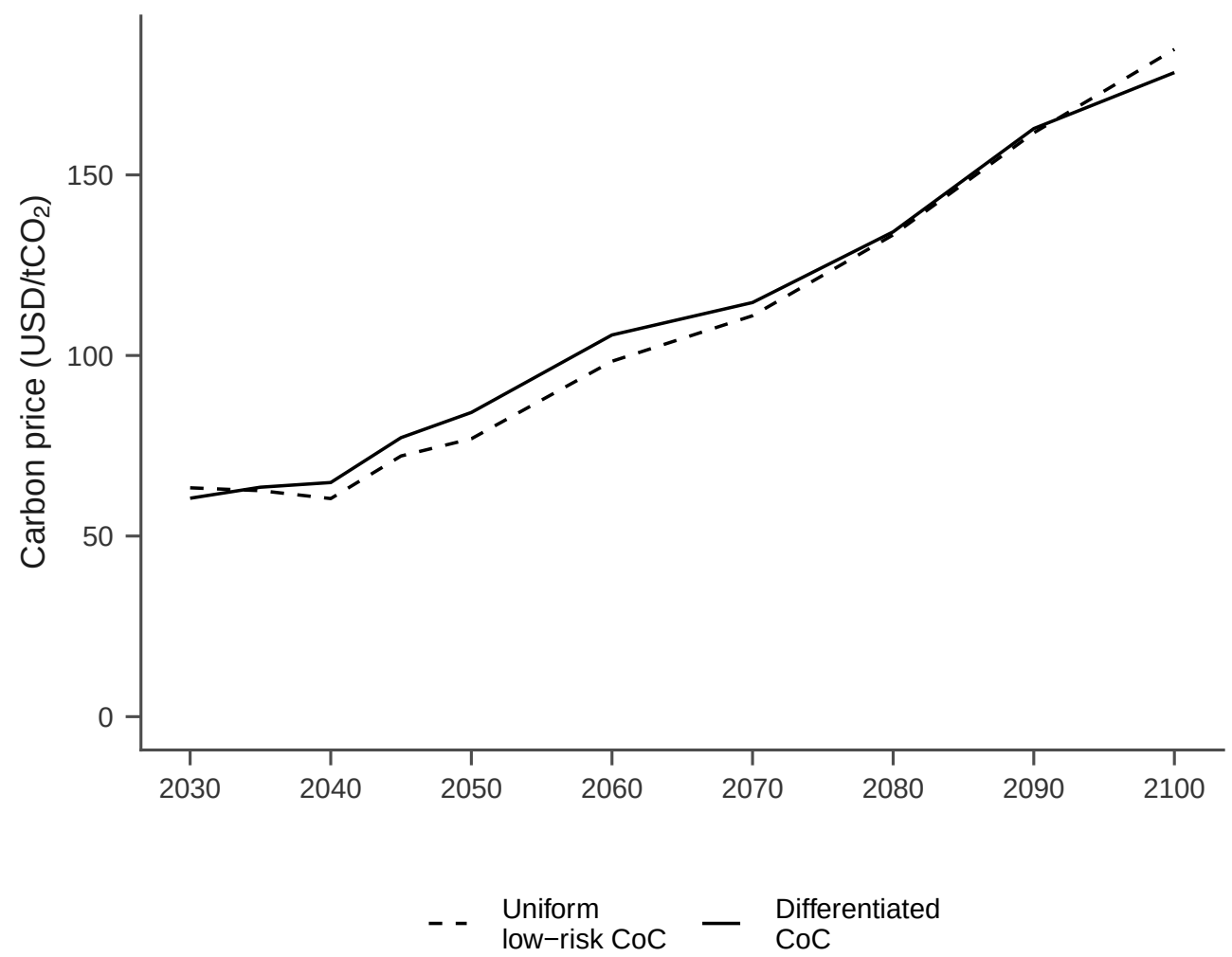

Figure S16. Carbon prices for the well below +2°C scenario for the *Uniform low-risk CoC* and the *Differentiated CoC*.

concentration target) into regional abatement levels and costs. Additional information can be found in IMAGE's model documentation[55].

Electricity grid investments in IMAGE Grid costs in IMAGE are modelled in an aggregated way. Investments in electricity generation capacity require physical grid investments/expansion. This physical grid need is translated to the monetary grid capital required. The grid sector's CoC is used to translate this capital into annual grid costs. The resulting grid costs are then included in the electricity prices for the different electricity demand sectors (mimicking grid tariffs). Overall, *physical* grid needs are determined by power generation, while the grid sector's CoC affects the *monetary* cost per physical grid "unit" (e.g., km of cables).

Against this background, the shifts in Fig. 4a combine two separate effects. First, changes in the power generation CoC alter the physical grid needs (i.e., a higher CoC leads to less grid investment). Second, changes in the electricity network CoC alter the costs of meeting these physical grid needs (i.e., a higher grid CoC leads to higher monetary investment for the same physical grid unit). Therefore, the sign of the net effect can vary.

Hydrogen cost in IMAGE IMAGE features a centralized hydrogen supply sector, for which we present results. Hydrogen costs are based on levelized costs that feature capital expenditures (subject to technology learning and converted to an annuity based on the CoC), fuel costs, operating expenditures and, for blue hydrogen, carbon capture costs. The key channel for the CoC to increase hydrogen costs is to increase the financing cost for capital expenditures, but changes to fuel costs (e.g., electricity for electrolysis) can also affect costs. If hydrogen demand exceeds a certain threshold, the sector switches from small-scale technologies to cheaper large-scale technologies.

Notably, this sector does not include legacy hydrogen use cases as a feedstock for chemicals and refineries. These legacy volumes are accounted for outside the hydrogen production sector, but their learning effects for technology costs affect the sector (i.e., legacy hydrogen production capacity is included in the cumulative capacity driving the centralized hydrogen supply learning curves). As all our runs exceed the demand volume threshold for larger-scale technologies by 2030 already, adding these legacy volumes explicitly into the hydrogen supply sector would not alter technology availability and, given the limited feedback between total demand and hydrogen costs in IMAGE, is unlikely to alter our findings, particularly as we do not report absolute hydrogen costs but just relative changes due to CoC differentiation.

Other Supplementary Figures and Tables

Table S9. Regional CoC estimates by sector for 2025. Values show the GDP-weighted regional average; parentheses indicate the range across countries within the region. Single-country regions show no range. Differences with values in Figure 1 are due to GDPpc growth and the resulting country risk reductions between 2025 and 2030.

IMAGE region	Electricity	Heat	Biomass	Pulp & paper	Iron & steel	Hydrogen	Cement	Food processing
Canada	4.5%	4.5%	6.0%	5.2%	6.5%	6.9%	6.2%	5.2%
China	5.3% (5.1–11.6%)	5.3% (5.1–11.6%)	6.8% (6.7–13.8%)	6.0% (5.9–12.5%)	7.3% (7.2–14.2%)	7.8% (7.6–15.2%)	7.1% (7.0–14.2%)	6.1% (5.9–13.2%)
Japan	5.1%	5.1%	6.7%	5.8%	7.2%	7.7%	7.0%	6.0%
Korea	5.1% (5.1–16.6%)	5.1% (5.1–16.6%)	6.6% (6.6–19.2%)	5.8% (5.8–17.6%)	7.1% (7.1–19.6%)	7.5% (7.5–21.0%)	6.9% (6.9–19.9%)	5.8% (5.8–18.8%)
Oceania	4.4% (4.4–11.7%)	4.4% (4.4–11.7%)	5.9% (5.9–14.0%)	5.1% (5.1–12.6%)	6.4% (6.4–14.4%)	6.8% (6.8–15.5%)	6.2% (6.2–14.5%)	5.2% (5.1–13.5%)
USA	4.5%	4.5%	6.0%	5.3%	6.5%	6.9%	6.3%	5.2%
Western Europe	5.4% (4.4–10.9%)	5.4% (4.4–10.9%)	6.9% (5.9–12.9%)	6.1% (5.1–11.8%)	7.4% (6.4–13.4%)	7.8% (6.8–14.2%)	7.2% (6.2–13.4%)	6.2% (5.1–12.3%)
Brazil	7.3%	7.3%	9.2%	8.1%	9.6%	10.4%	9.6%	8.6%
Central Europe	6.5% (5.3–12.5%)	6.5% (5.3–12.5%)	8.1% (6.9–14.5%)	7.3% (6.0–13.4%)	8.7% (7.4–15.0%)	9.0% (7.8–15.6%)	8.4% (7.2–14.8%)	7.3% (6.1–13.7%)
India	6.6%	6.6%	8.3%	7.4%	8.8%	9.5%	8.7%	7.7%
Indonesia	6.9% (6.8–10.2%)	6.9% (6.8–10.2%)	8.5% (8.5–12.3%)	7.6% (7.6–11.1%)	9.0% (9.0–12.7%)	9.5% (9.5–13.7%)	8.9% (8.8–12.8%)	7.8% (7.7–11.8%)
Kazakhstan region	8.0% (7.0–11.8%)	8.0% (7.0–11.8%)	9.7% (8.6–13.9%)	8.8% (7.7–12.7%)	10.2% (9.1–14.3%)	10.7% (9.6–15.1%)	10.0% (8.9–14.3%)	8.9% (7.9–13.2%)
Mexico	6.0%	6.0%	7.6%	6.7%	8.1%	8.7%	8.0%	6.9%
Middle East	7.7% (5.1–20.8%)	7.7% (5.1–20.8%)	9.4% (6.6–23.6%)	8.5% (5.8–21.9%)	9.9% (7.1–24.0%)	10.4% (7.5–25.5%)	9.8% (6.9–24.3%)	8.7% (5.8–23.3%)
Rest of Central America	8.6% (6.0–15.3%)	8.6% (6.0–15.3%)	10.5% (7.4–18.0%)	9.5% (6.8–16.3%)	11.0% (8.0–18.3%)	11.7% (8.1–19.7%)	10.9% (7.6–18.6%)	9.9% (6.5–17.6%)
Rest of South America	9.0% (5.2–19.7%)	9.0% (5.2–19.7%)	11.0% (6.8–23.1%)	9.8% (6.0–20.9%)	11.4% (7.3–23.3%)	12.3% (7.8–25.4%)	11.5% (7.1–23.9%)	10.4% (6.1–23.0%)
Russia	8.4% (7.7–15.4%)	8.4% (7.7–15.4%)	10.2% (9.4–17.8%)	9.2% (8.5–16.4%)	10.7% (9.9–18.2%)	11.4% (10.5–19.3%)	10.6% (9.8–18.3%)	9.5% (8.7–17.3%)
South Africa	7.2%	7.2%	8.9%	8.0%	9.4%	10.0%	9.3%	8.3%
Southeast Asia	7.1% (4.8–14.6%)	7.1% (4.8–14.6%)	8.8% (6.2–17.1%)	7.9% (5.5–15.6%)	9.3% (6.8–17.5%)	9.8% (7.0–18.8%)	9.1% (6.5–17.7%)	8.1% (5.4–16.7%)
Turkey	9.2%	9.2%	11.2%	10.1%	11.6%	12.4%	11.6%	10.5%
Eastern Africa	11.5% (6.9–20.7%)	11.5% (6.9–20.7%)	13.7% (8.5–23.9%)	12.4% (7.7–21.8%)	14.1% (9.0–24.1%)	15.2% (9.4–26.1%)	14.2% (8.8–24.7%)	13.2% (7.7–23.7%)
Northern Africa	10.7% (6.9–11.7%)	10.7% (6.9–11.7%)	12.8% (8.7–13.7%)	11.6% (7.7–12.6%)	13.2% (9.2–14.2%)	14.1% (9.9–15.0%)	13.2% (9.1–14.1%)	12.2% (8.1–13.1%)
Rest of South Asia	10.5% (8.5–12.9%)	10.5% (8.5–12.9%)	12.6% (10.4–15.1%)	11.4% (9.3–13.8%)	13.0% (10.9–15.5%)	14.0% (11.7–16.6%)	13.1% (10.9–15.6%)	12.1% (9.8–14.6%)
Rest of Southern Africa	11.0% (5.7–13.9%)	11.0% (5.7–13.9%)	13.2% (7.3–16.5%)	11.9% (6.5–14.9%)	13.6% (7.8–16.9%)	14.6% (8.2–18.3%)	13.7% (7.6–17.1%)	12.7% (6.5–16.1%)
Ukraine region	14.9% (12.4–15.1%)	14.9% (12.4–15.1%)	17.2% (14.4–17.4%)	15.9% (13.3–16.1%)	17.6% (14.9–17.8%)	18.7% (15.5–18.9%)	17.7% (14.8–17.9%)	16.7% (13.7–16.9%)
Western Africa	10.9% (8.4–15.4%)	10.9% (8.4–15.4%)	13.1% (10.2–18.1%)	11.8% (9.2–16.5%)	13.5% (10.7–18.4%)	14.5% (11.4–19.9%)	13.6% (10.6–18.7%)	12.5% (9.6–17.7%)

	Imputed countries	IMAGE region	Country peers in the same region used for imputation
1	Sudan, Somalia	E. Africa	Ethiopia, Kenya, Madagascar, Mauritius, Rwanda, Uganda
2	North Korea	Korea	South Korea
3	Iran, Syria, Yemen	Middle East	United Arab Emirates, Bahrain, Iraq, Israel, Jordan, Kuwait, Lebanon, Oman, Qatar, Saudi Arabia
4	Haiti	Rest C. America	Aruba, Bahamas, Belize, Bermuda, Barbados, Costa Rica, Cuba, Curaçao, Cayman Islands, Dominican Republic, Guatemala, Honduras, Jamaica, Montserrat, Nicaragua, Panama, El Salvador, Turks & Caicos Islands, Trinidad & Tobago, St. Vincent & Grenadines
5	Malawi, Zimbabwe	Rest S. Africa	Angola, Botswana, Mozambique, Namibia, Eswatini, Tanzania, Zambia
6	Guinea, Gambia, Guinea-Bissau, Liberia, Sierra Leone	W. Africa	Benin, Burkina Faso, Côte d'Ivoire, Cameroon, Congo - Kinshasa, Congo - Brazzaville, Cape Verde, Gabon, Ghana, Mali, Niger, Nigeria, Senegal, Togo

Table S10. Imputation of corporate tax rates

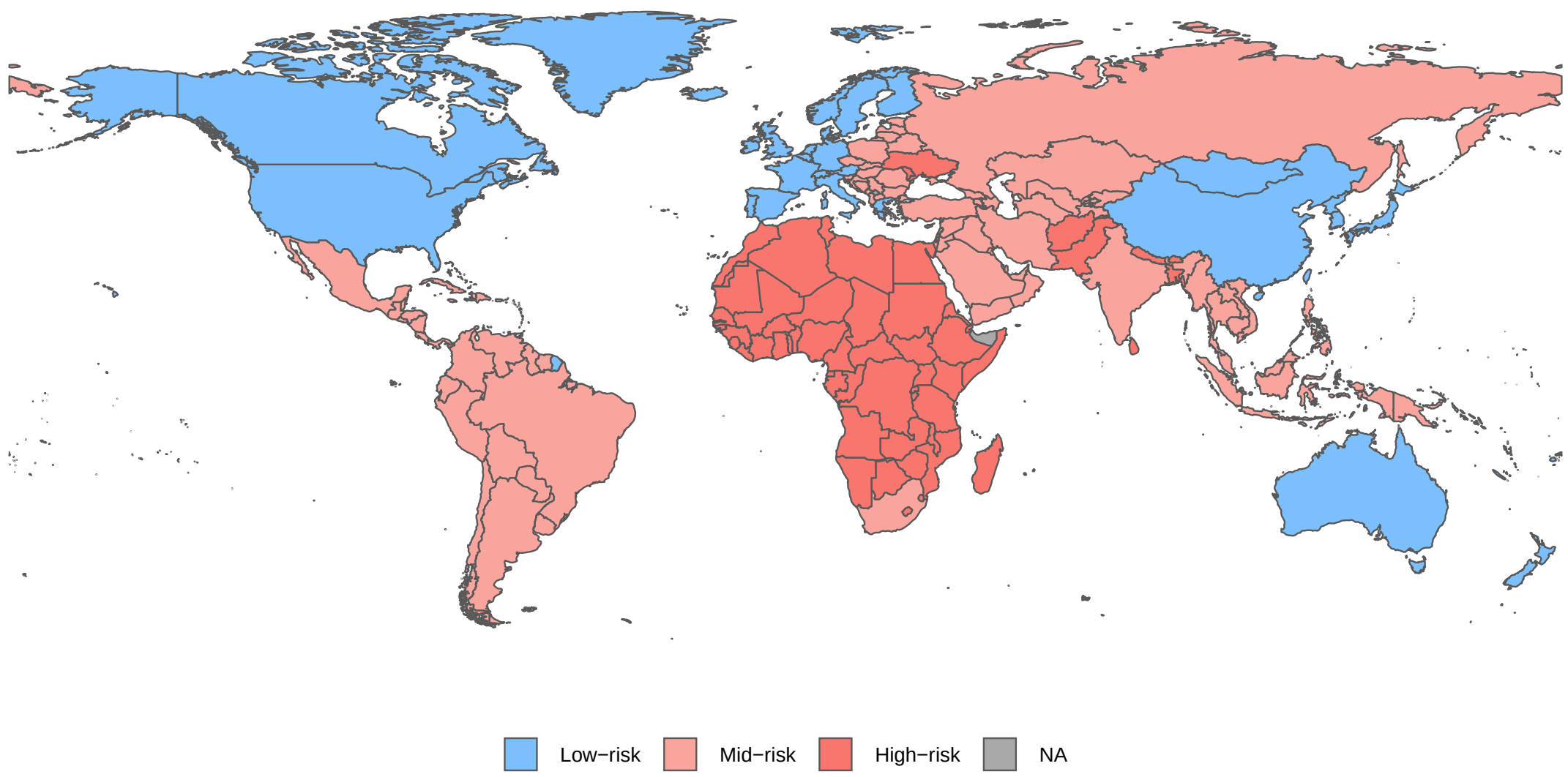

Figure S17. CoC-based macro regions to which the 26 IMAGE regions are aggregated. Country-level territories in grey are not (explicitly) represented in IMAGE. Map data from Natural Earth via the rnaturalearth R package[51].

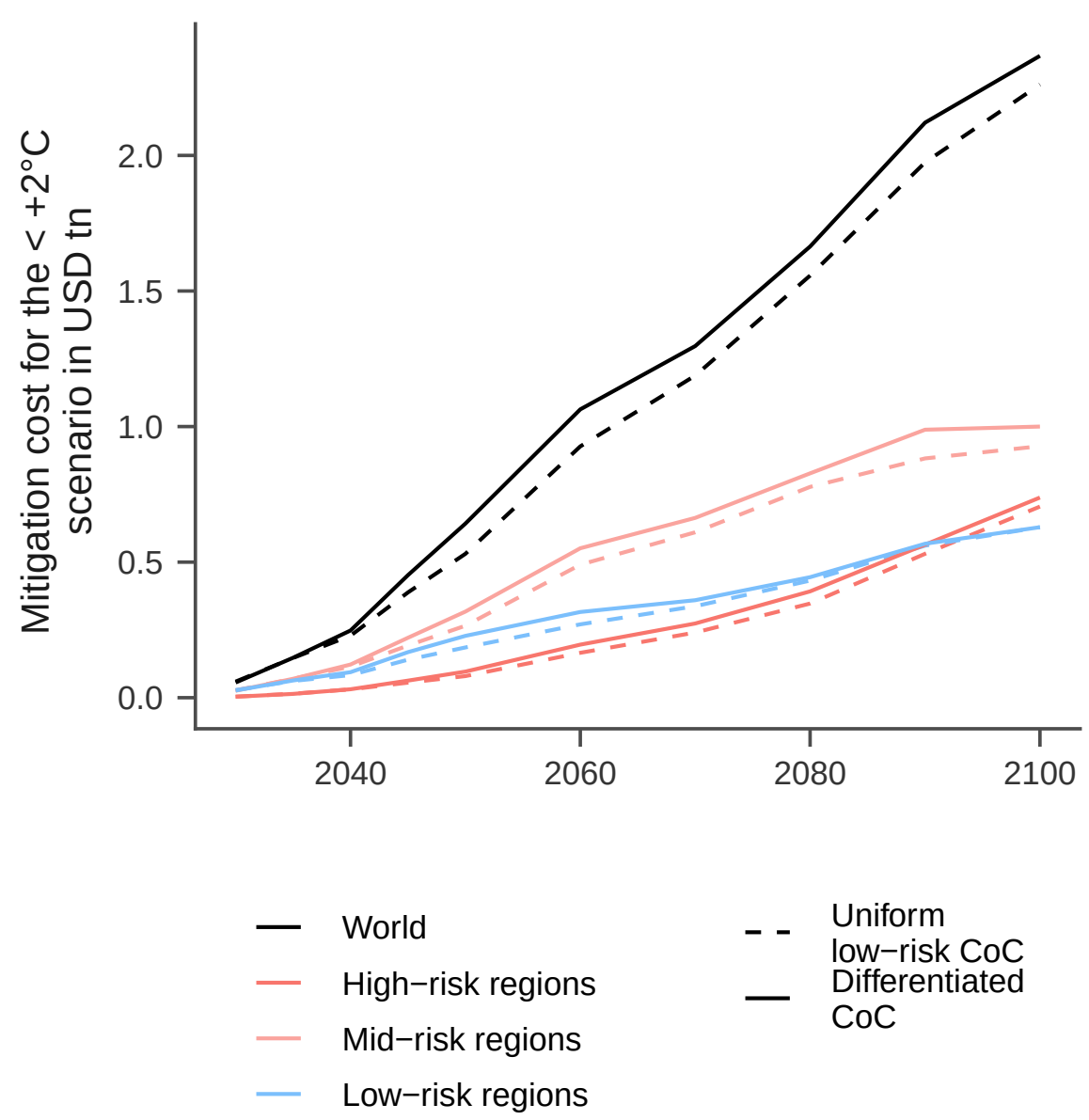

Figure S18. Mitigation cost for the well below +2°C scenario worldwide and by macro region

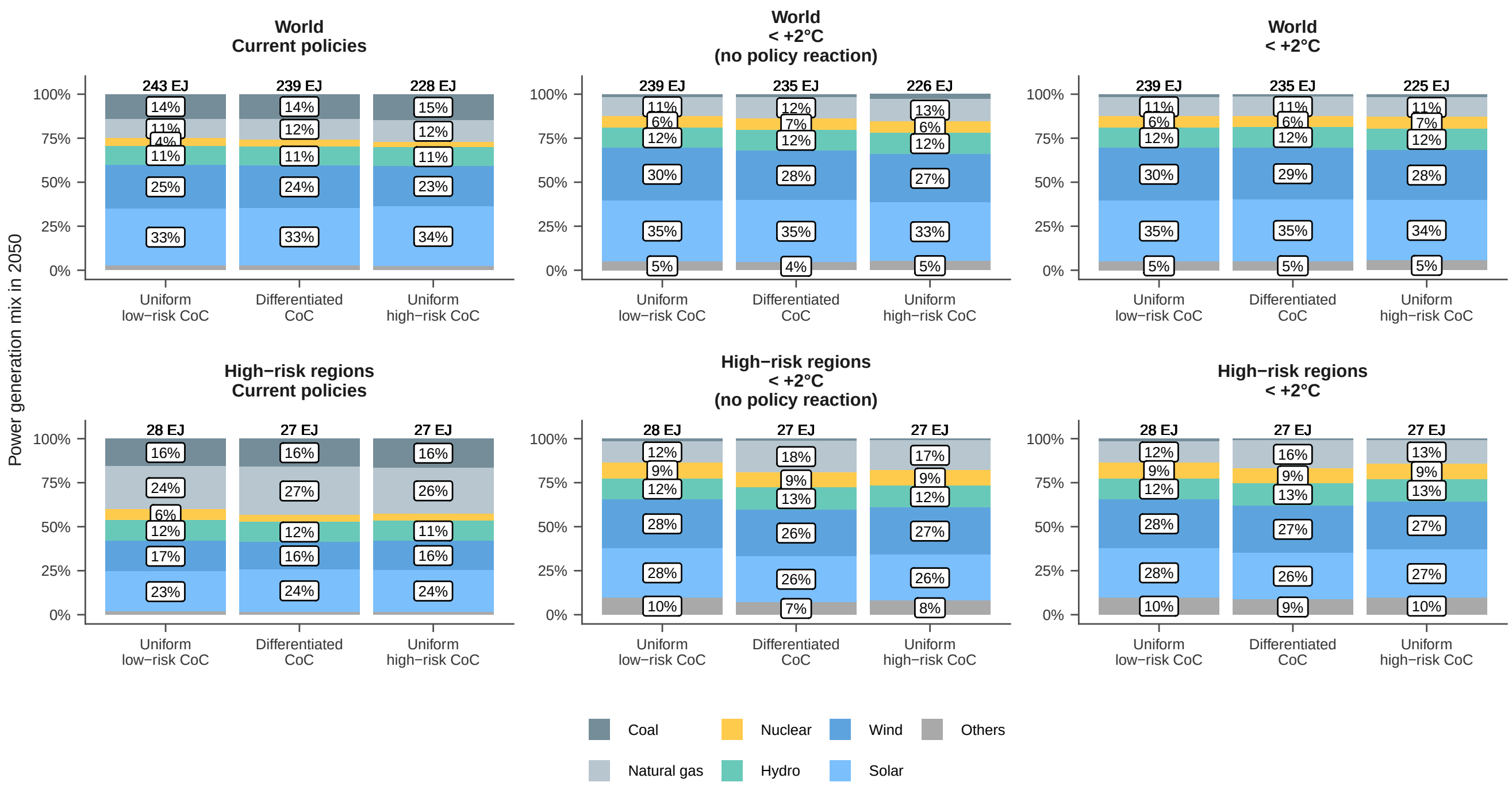

Figure S19. Power generation mix by 2050 worldwide and in high-risk regions

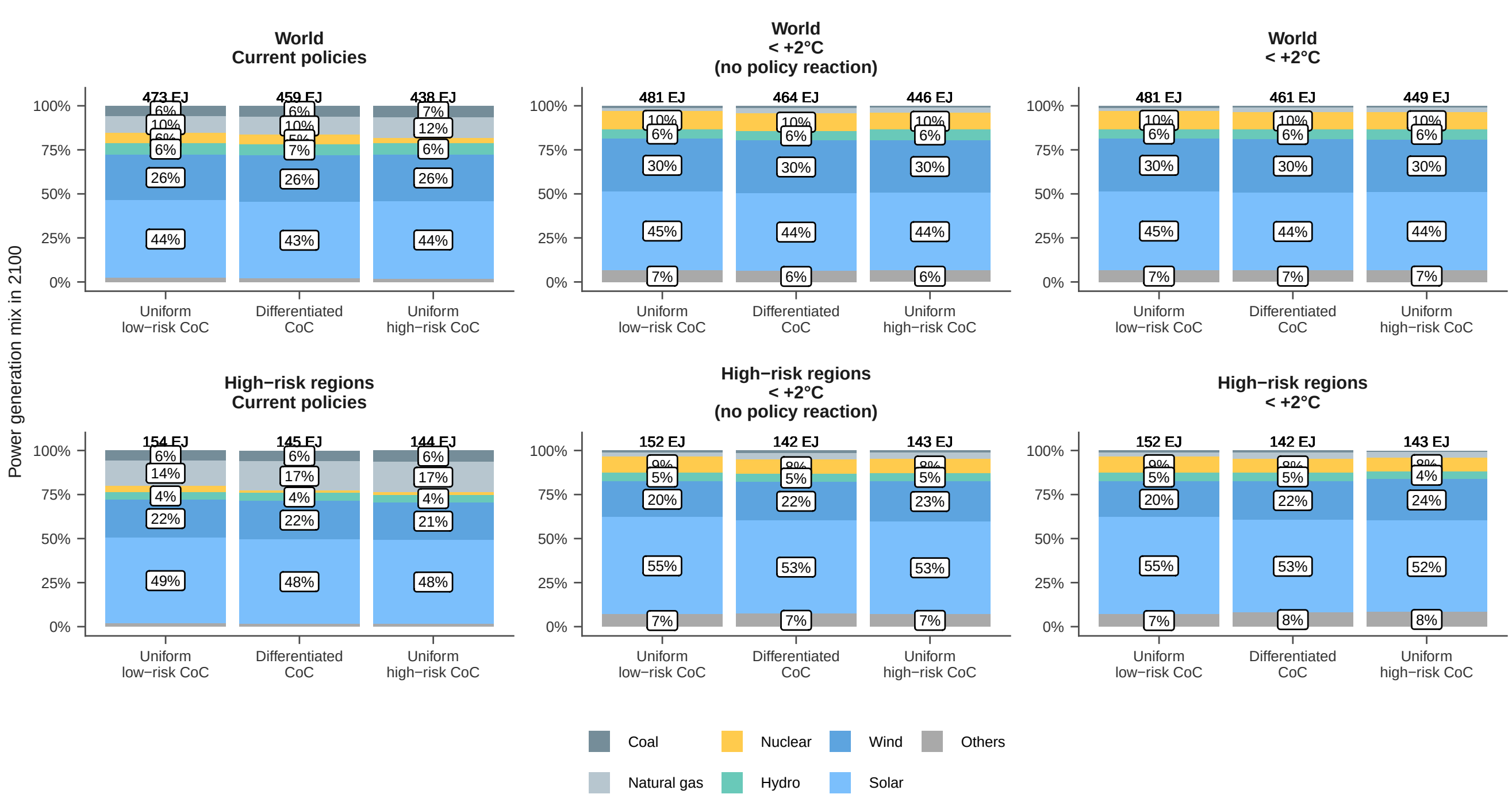

Figure S20. Power generation mix by 2100 worldwide and in high-risk regions

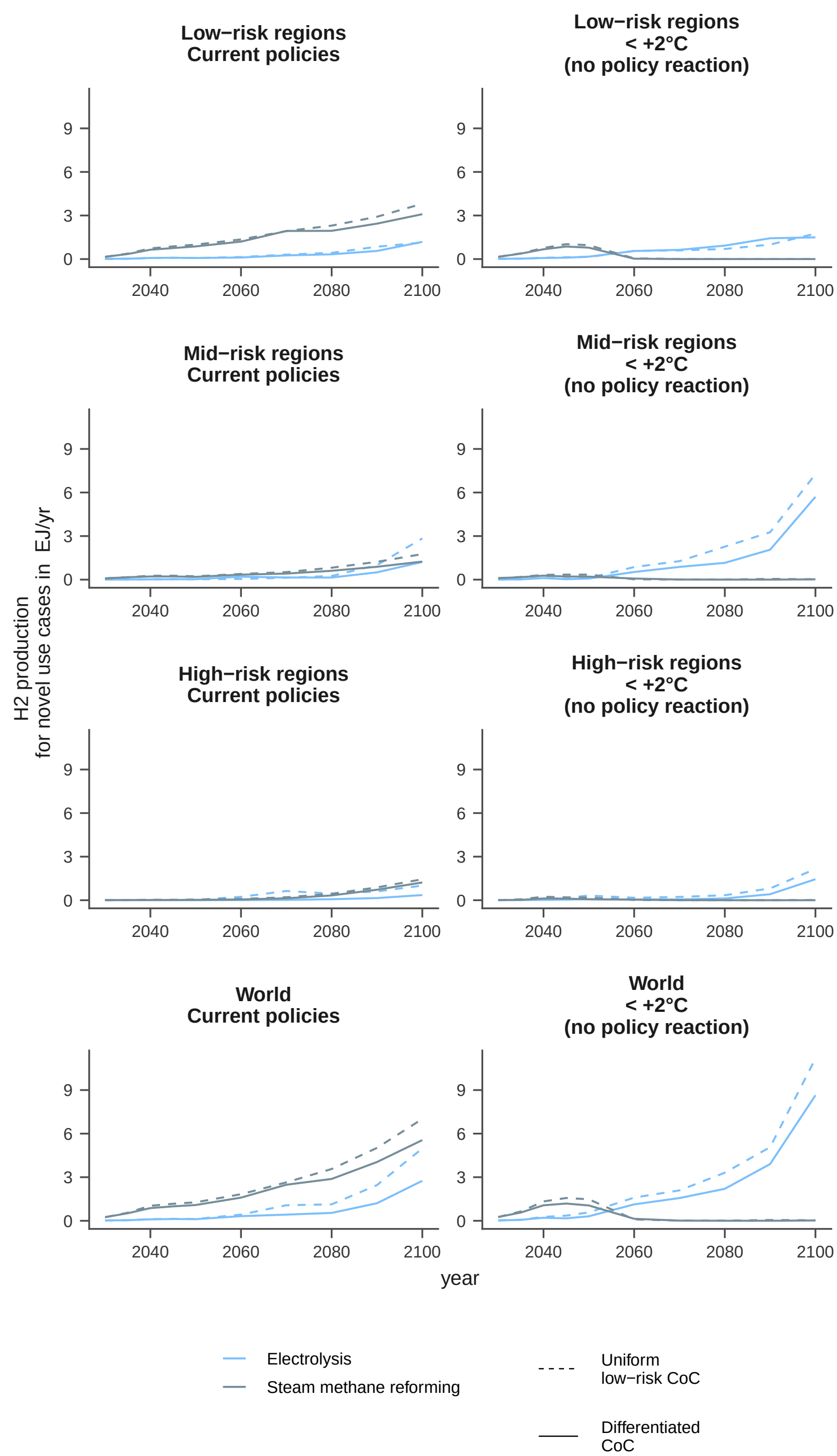

Figure S21. Hydrogen production by macro region and scenario (excluding legacy use cases, see Supplementary Note 5)

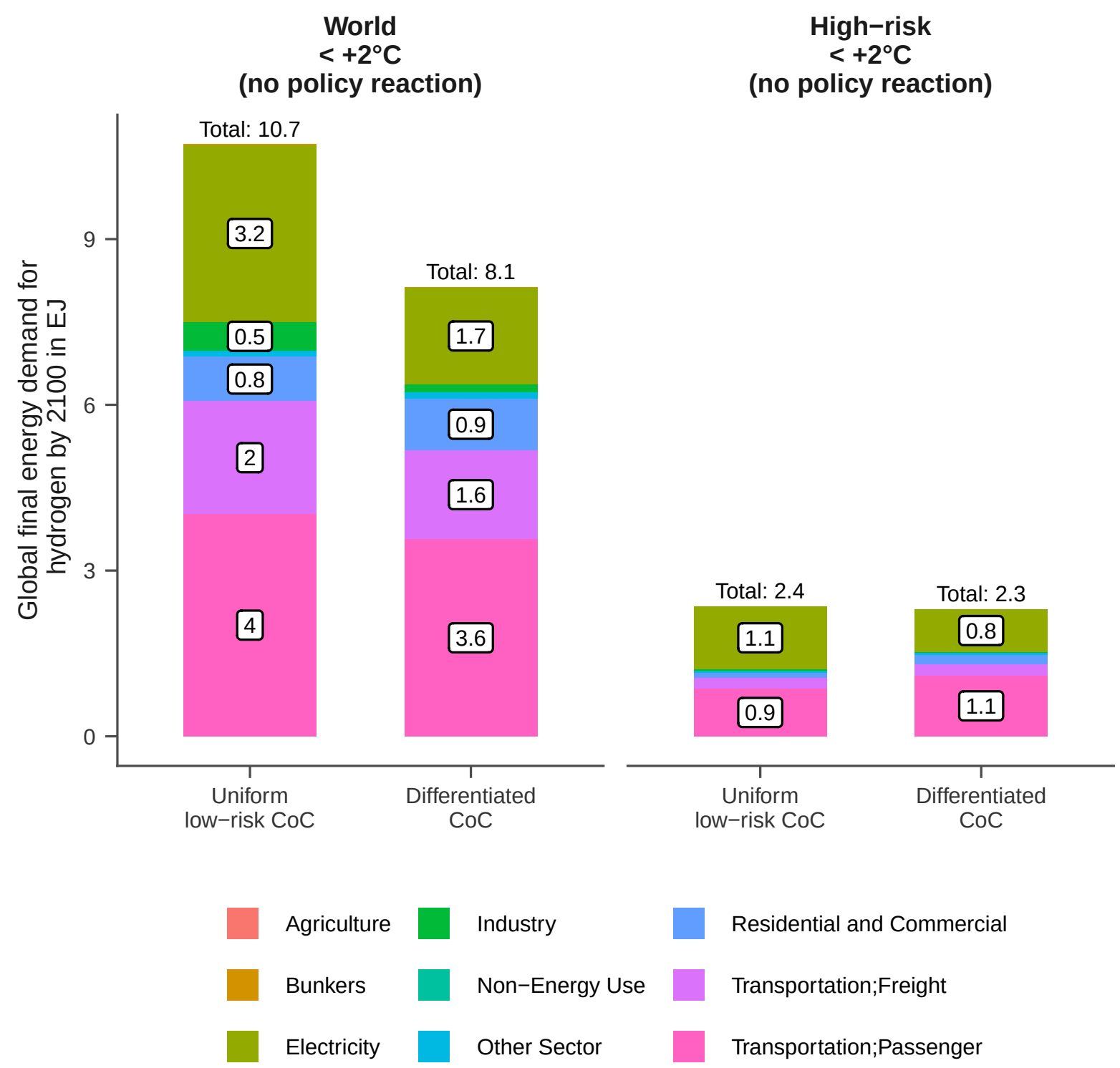

Figure S22. Final energy demand in hydrogen by sector under the well below +2°C scenario (excluding legacy use cases, see Supplementary Note 5)

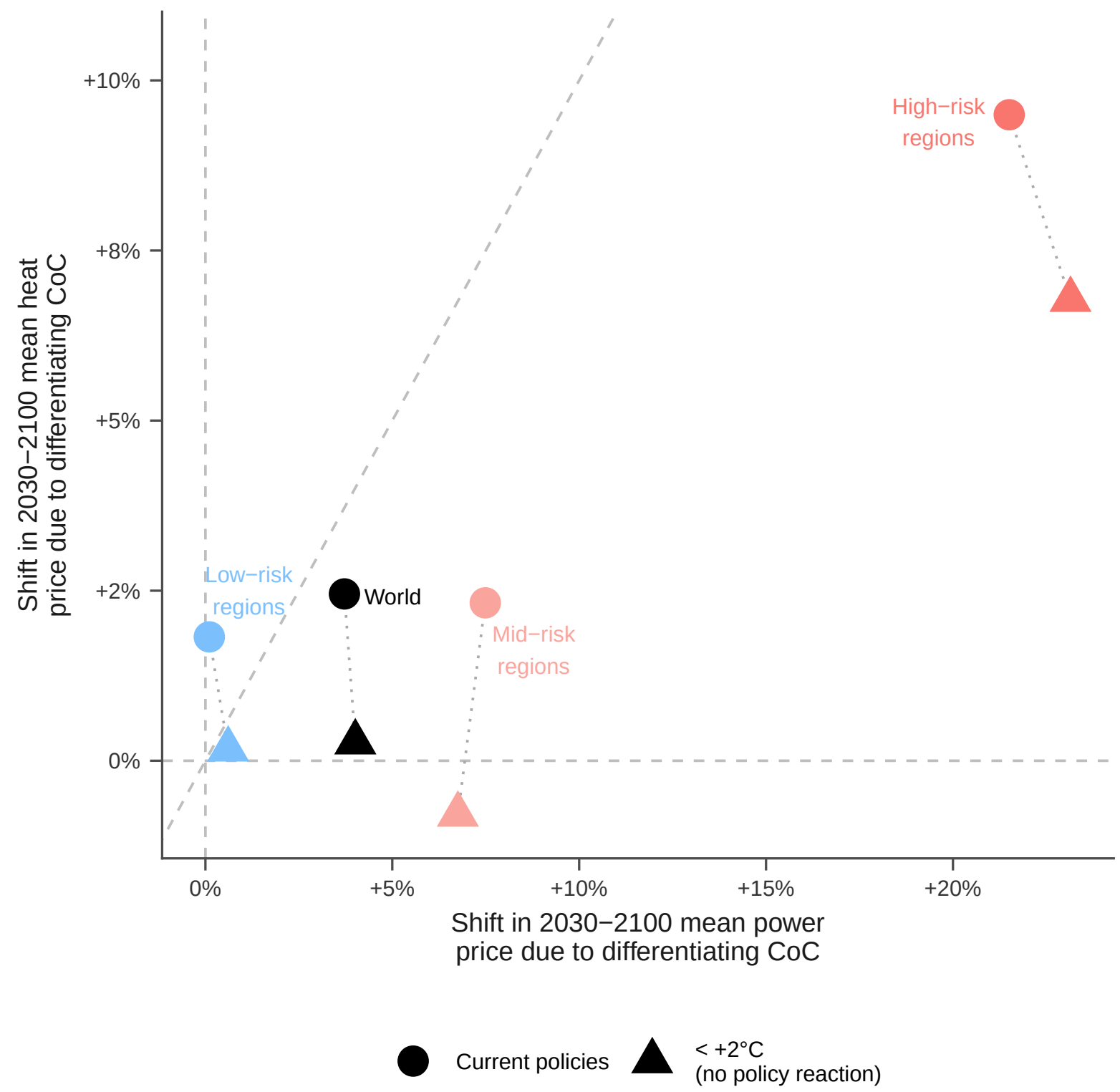

Figure S23. Impact of cost of capital differentiation on mean 2030–2100 power and heat prices

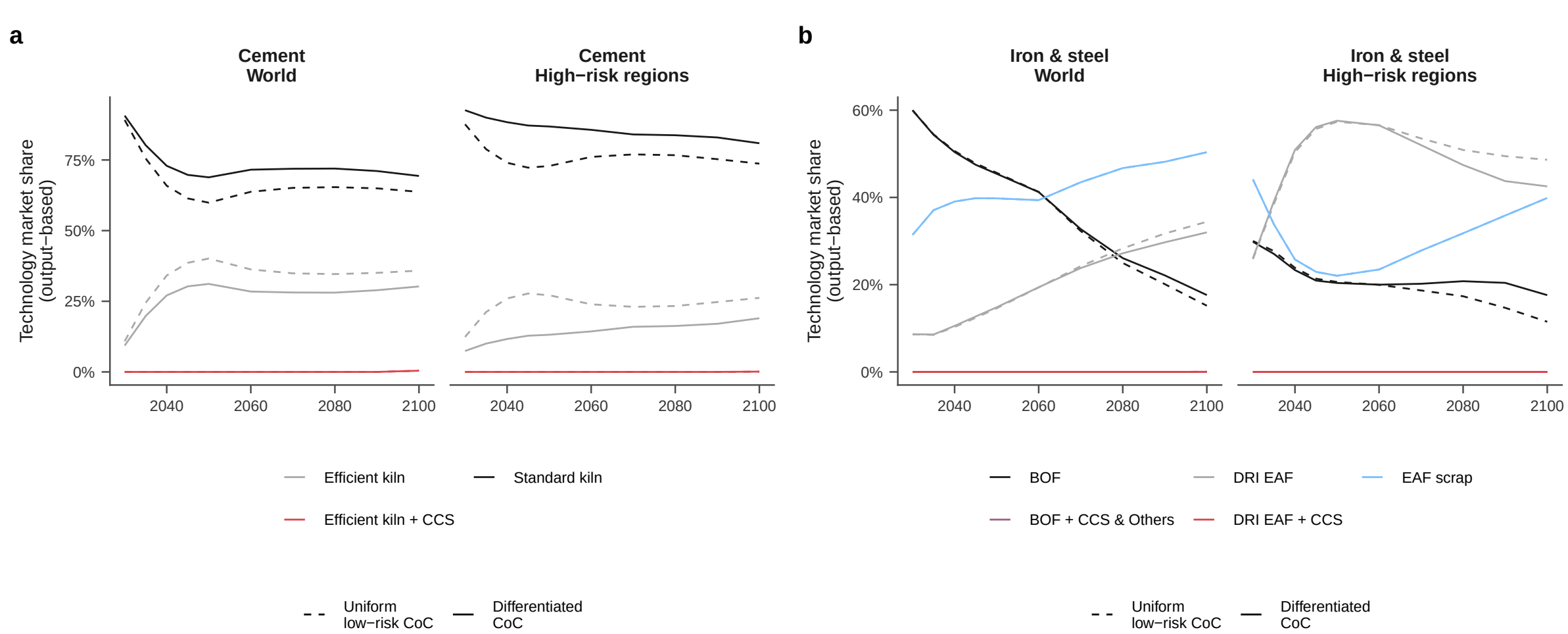

Figure S24. Cement and steel market shares by technology under the current policies scenario. “BOF” denotes the blast furnace basic oxygen furnace route. “EAF” and “DRI” denote electric arc furnace and direct reduced iron, respectively.

References for the Supplementary Information

1. Steffen, B. The importance of project finance for renewable energy projects. *Energy Economics* **69**, 280–294 (2018). DOI: 10.1016/j.eneco.2017.11.006.
2. Esty, B. C. Why Study Large Projects? An Introduction to Research on Project Finance. *European Financial Management* **10**, 213–224 (2004). DOI: 10.1111/j.1354-7798.2004.00247.x.
3. IRENA. The cost of financing for renewable power. Abu Dhabi, (2023). URL: https://www.irena.org/Publications/2023/May/The-cost-of-financing-for-renewable-power (visited on 08/03/2023).
4. Damodaran, A. Investment valuation: Tools and techniques for determining the value of any asset. Fourth edition. Hoboken, New Jersey: Wiley, (2025).
5. Steffen, B. & Waidelich, P. Determinants of cost of capital in the electricity sector. *Progress in Energy* **4**, 033001 (2022). DOI: 10.1088/2516-1083/ac7936.
6. Steffen, B. Estimating the cost of capital for renewable energy projects. *Energy Economics* **88**, 104783 (2020). DOI: 10.1016/j.eneco.2020.104783.
7. Polzin, F. et al. The effect of differentiating costs of capital by country and technology on the European energy transition. *Climatic Change* **167**, 26 (2021). DOI: 10.1007/s10584-021-03163-4.
8. Calcaterra, M. et al. Reducing the cost of capital to finance the energy transition in developing countries. *Nature Energy* **9**, 1241–1251 (2024). DOI: 10.1038/s41560-024-01606-7.
9. Steffen, B., Egli, F., Gumber, A., Đukan, M., & Waidelich, P. A global dataset of the cost of capital for renewable energy projects. *Scientific Data* **12**, 1624 (2025). DOI: 10.1038/s41597-025-05912-x.
10. Mazzucato, M. & Semieniuk, G. Financing renewable energy: Who is financing what and why it matters. *Technological Forecasting and Social Change* **127**, 8–22 (2018). DOI: 10.1016/j.techfore.2017.05.021.
11. Waidelich, P. & Steffen, B. Renewable energy financing by state investment banks: Evidence from OECD countries. *Energy Economics* **132**, 107455 (2024). DOI: 10.1016/j.eneco.2024.107455.
12. Egli, F., Steffen, B., & Schmidt, T. S. A dynamic analysis of financing conditions for renewable energy technologies. *Nature Energy* **3**, 1084–1092 (2018). DOI: 10.1038/s41560-018-0277-y.
13. Đukan, M., Gumber, A., Egli, F., & Steffen, B. The role of policies in reducing the cost of capital for offshore wind. *iScience* **26**, 106945 (2023). DOI: 10.1016/j.isci.2023.106945.
14. Censkowsky, P., Waidelich, P., Shishlov, I., & Steffen, B. Quantifying the shift of public export finance from fossil fuels to renewable energy. *Nature Communications* **16**, 900 (2025). DOI: 10.1038/s41467-025-55981-0.
15. IEA. World Energy Outlook 2025. Paris, (2025). URL: https://www.iea.org/reports/world-energy-outlook-2025 (visited on 01/27/2026).
16. IEA. ETP Clean Energy Technology Guide. Paris: International Energy Agency, (2025). URL: https://www.iea.org/data-and-statistics/data-tools/etp-clean-energy-technology-guide (visited on 08/03/2025).
17. Hatton, L., Staffell, I., Jansen, M., Oluleye, G., & Hawkes, A. Historical and future projected costs of capital for ten energy technologies across 176 countries. *Scientific Data* **12**, 1912 (2025). DOI: 10.1038/s41597-025-06177-0.
18. Damodaran, A. Damodaran dataset: Levered and Unlevered Betas by Industry (version: January 2025 update; file path: Data > Archived Data > Risk/Discount Rate > Levered and Unlevered Betas by Industry > Global > 1/25). (2025). URL: https://pages.stern.nyu.edu/~adamodar/.
19. Damodaran, A. Damodaran dataset (version: January 2025 update). (2025). URL: https://pages.stern.nyu.edu/~adamodar/.
20. Kroll. Kroll Cost of Capital Navigator (accessed: January 2026). (2026). URL: https://www.kroll.com/en/tools-and-platforms/cost-of-capital.

21. Damodaran, A. Equity Risk Premiums (ERP): Determinants, Estimation, and Implications: The 2025 Edition. *SSRN* (2025). DOI: 10.2139/ssrn.5168609.

22. Harrington, J., Nunes, C. S., & Aboulamer, A. Valuation Handbook - International Guide to Cost of Capital: 2023 Summary Edition. New York: Kroll, (2023). DOI: 10.56227/24.1.6.

23. Pratt, S. P. & Grabowski, R. J. Cost of capital: Applications and examples. Fifth edition. Wiley finance. Hoboken, New Jersey: Wiley, (2014).

24. Bachner, G., Mayer, J., & Steininger, K. W. Costs or benefits? Assessing the economy-wide effects of the electricity sector's low carbon transition – The role of capital costs, divergent risk perceptions and premiums. *Energy Strategy Reviews* **26**, 100373 (2019). DOI: 10.1016/j.esr.2019.100373.

25. OECD. Interest Rates: Long-Term Government Bond Yields: 10-Year: Main (Including Benchmark) for United States [IRLTLT01USM156N], retrieved from FRED, Federal Reserve Bank of St. Louis. Paris, (2025). URL: https://fred.stlouisfed.org/series/IRLTLT01USM156N (visited on 07/03/2025).

26. Egli, F., Steffen, B., & Schmidt, T. S. Bias in energy system models with uniform cost of capital assumption. *Nature Communications* **10**, 4588 (2019). DOI: 10.1038/s41467-019-12468-z.

27. U.S. Office of Management and Budget. Circular No. A-94 (Revised): Guidelines and Discount Rates for Benefit-Cost Analysis of Federal Programs. OMB Circular. Issued October 29, 1992. Washington, D.C.: Executive Office of the President, (Oct. 1992).

28. Ameli, N. et al. Higher cost of finance exacerbates a climate investment trap in developing economies. *Nature Communications* **12**, 4046 (2021). DOI: 10.1038/s41467-021-24305-3.

29. Ekholm, T., Ghoddusi, H., Krey, V., & Riahi, K. The effect of financial constraints on energy-climate scenarios. *Energy Policy* **59**, 562–572 (2013). DOI: 10.1016/j.enpol.2013.04.001.

30. Halstead, M., Donker, J., Longa, F. D., & Zwaan, B. van der. The importance of fossil fuel divestment and competitive procurement for financing Europe's energy transition. *Journal of Sustainable Finance & Investment* **9**, 349–355 (2019). DOI: 10.1080/20430795.2019.1619339.

31. Emmerling, J. et al. The role of the discount rate for emission pathways and negative emissions. *Environmental Research Letters* **14**, 104008 (2019). DOI: 10.1088/1748-9326/ab3cc9.

32. Krey, V. et al. Looking under the hood: A comparison of techno-economic assumptions across national and global integrated assessment models. *Energy* **172**, 1254–1267 (2019). DOI: 10.1016/J.ENERGY.2018.12.131.

33. Iyer, G. C. et al. Improved representation of investment decisions in assessments of CO2 mitigation. *Nature Climate Change* **5**, 436–440 (2015). DOI: 10.1038/nclimate2553.

34. Mandel, A., Battiston, S., & Monasterolo, I. Mapping global financial risks under climate change. *Nature Climate Change* **15**, 329–334 (2025). DOI: 10.1038/s41558-025-02244-x.

35. BVR. Cost of Capital Professional. Portland, OR, (2025). URL: https://www.bvresources.com/docs/default-source/spec-sheets/cost-of-capital-pro-spec-sheet.pdf?sfvrsn=ea3ccdb2_22 (visited on 01/27/2026).

36. Vasicek, O. A. A note on using cross-sectional information in Bayesian estimation of security betas. *The Journal of Finance* **28**, 1233–1239 (1973). DOI: 10.1111/j.1540-6261.1973.tb01452.x.

37. Hamada, R. S. The Effect of the Firm's Capital Structure on the Systematic Risk of Common Stocks. *The Journal of Finance* **27**, 435–452 (1972). DOI: 10.2307/2978486.

38. Harris, R. S. & Pringle, J. J. Risk-adjusted discount rates—Extensions from the average-risk case. *Journal of Financial Research* **8**, 237–244 (1985). DOI: 10.1111/j.1475-6803.1985.tb00406.x.

39. Erb, C. B., Harvey, C. R., & Viskanta, T. E. Expected Returns and Volatility in 135 Countries. *The Journal of Portfolio Management* **22**, 46–58 (1996). DOI: 10.3905/jpm.1996.409554.

40. Damodaran, A. Damodaran dataset: Risk Premiums for Other Markets (version: January updates for 2016–2025; file path: Data > Archived Data > Risk/Discount Rate > Risk Premiums for Other Markets > 1/25, 1/24, etc.) (2025). URL: https://pages.stern.nyu.edu/~adamodar/.

41. World Bank. World Development Indicators [GDP per capita, PPP (constant 2021 international $), series code NY.GDP.PCAP.PP.KD]. Washington, DC, (2025). URL: https://databank.worldbank.org/source/world-development-indicators.

42. Hu, Y T., Kiesel, R., & Perraudin, W. The estimation of transition matrices for sovereign credit ratings. *Journal of Banking & Finance* **26**, 1383–1406 (2002). DOI: 10.1016/S0378-4266(02)00268-6.

43. De Moor, L., Luitel, P., Sercu, P., & Vanpee, R. Subjectivity in sovereign credit ratings. *Journal of Banking & Finance* **88**, 366–392 (2018). DOI: 10.1016/j.jbankfin.2017.12.014.

44. Fuchs, A. & Gehring, K. The Home Bias in Sovereign Ratings. *Journal of the European Economic Association* **15**, 1386–1423 (2017). DOI: 10.1093/jeea/jvx009.

45. Ambrocio, G. & Hasan, I. Quid pro quo? Political ties and sovereign borrowing. *Journal of International Economics* **133**, 103523 (2021). DOI: 10.1016/j.jinteco.2021.103523.

46. Cuadros-Solas, P. J., Salvador, C., & Suárez, N. Am I riskier if I rescue my banks? Beyond the effects of bailouts. *Journal of Financial Stability* **56**, 100935 (2021). DOI: 10.1016/j.jfs.2021.100935.

47. Cuadros-Solas, P. J., Salvador, C., & Suárez, N. Banking supervisory architecture and sovereign risk. *Journal of Financial Stability* **76**, 101365 (2025). DOI: 10.1016/j.jfs.2024.101365.

48. Jin, Y., Hou, K., & Yang, G. Discrimination and its self-fulfilling nature in sovereign credit ratings. *Finance Research Letters* **85**, 107869 (2025). DOI: 10.1016/j.frl.2025.107869.

49. Cantor, R. M. & Packer, F. Determinants and Impact of Sovereign Credit Ratings. *Economic Policy Review* **2**, 37–53 (1996). DOI: 10.2139/ssrn.1028774.

50. Afonso, A., Gomes, P., & Rother, P. Short- and long-run determinants of sovereign debt credit ratings. *International Journal of Finance & Economics* **16**, 1–15 (2011). DOI: 10.1002/ijfe.416.

51. Massicotte, P., South, A., & Hufkens, K. rnaturalearth: World Map Data from Natural Earth. R package version 1.1.0. (2025). URL: https://docs.ropensci.org/rnaturalearth/.

52. van Ruijven, B. J. et al. Long-term model-based projections of energy use and CO2 emissions from the global steel and cement industries. *Resources, Conservation and Recycling* **112**, 15–36 (2016). DOI: 10.1016/j.resconrec.2016.04.016.

53. Daioglou, V., Mikropoulos, E., Gernaat, D., & van Vuuren, D. P. Efficiency improvement and technology choice for energy and emission reductions of the residential sector. *Energy* **243**, 122994 (2022). DOI: 10.1016/j.energy.2021.122994.

54. Girod, B., van Vuuren, D. P., & Deetman, S. Global travel within the 2°C climate target. *Energy Policy* **45**, 152–166 (2012). DOI: 10.1016/j.enpol.2012.02.008.

55. PBL. IMAGE framework. Den Haag, (2022). URL: https://models.pbl.nl/image/IMAGE_framework (visited on 09/09/2025).
