# Supplementary material for: The impact of financing cost differences on global energy and industry decarbonization
Source: Nat Clim Chang. 2026 Sep 24;16(10):1178–86. doi: 10.1038/s41558-026-02756-0 (PMC13638454; doi:10.1038/s41558-026-02756-0)
Supplement: Supplementary file 2 [file 41558_2026_2756_MOESM2_ESM.pdf]

Corresponding author(s): Paul Waidelich
Last updated by author(s): Aug 15, 2026

Reporting Summary

Nature Portfolio wishes to improve the reproducibility of the work that we publish. This form provides structure for consistency and transparency in reporting. For further information on Nature Portfolio policies, see our Editorial Policies and the Editorial Policy Checklist.

Statistics

For all statistical analyses, confirm that the following items are present in the figure legend, table legend, main text, or Methods section.

n/a	Confirmed	
☐	☒	The exact sample size (*n*) for each experimental group/condition, given as a discrete number and unit of measurement
☒	☐	A statement on whether measurements were taken from distinct samples or whether the same sample was measured repeatedly
☒	☐	The statistical test(s) used AND whether they are one- or two-sided<br>*Only common tests should be described solely by name; describe more complex techniques in the Methods section.*
☐	☒	A description of all covariates tested
☒	☐	A description of any assumptions or corrections, such as tests of normality and adjustment for multiple comparisons
☐	☒	A full description of the statistical parameters including central tendency (e.g. means) or other basic estimates (e.g. regression coefficient) AND variation (e.g. standard deviation) or associated estimates of uncertainty (e.g. confidence intervals)
☒	☐	For null hypothesis testing, the test statistic (e.g. *F*, *t*, *r*) with confidence intervals, effect sizes, degrees of freedom and *P* value noted<br>*Give P values as exact values whenever suitable.*
☒	☐	For Bayesian analysis, information on the choice of priors and Markov chain Monte Carlo settings
☒	☐	For hierarchical and complex designs, identification of the appropriate level for tests and full reporting of outcomes
☒	☐	Estimates of effect sizes (e.g. Cohen's *d*, Pearson's *r*), indicating how they were calculated

Our web collection on statistics for biologists contains articles on many of the points above.

Software and code

Policy information about availability of computer code

Data collection	IMAGE (version 3.4) was used.
Data analysis	RStudio (version 2025.05.2) based on R (version 4.5.2) was used.

For manuscripts utilizing custom algorithms or software that are central to the research but not yet described in published literature, software must be made available to editors and reviewers. We strongly encourage code deposition in a community repository (e.g. GitHub). See the Nature Portfolio guidelines for submitting code & software for further information.

Data

Policy information about availability of data

All manuscripts must include a data availability statement. This statement should provide the following information, where applicable:

- Accession codes, unique identifiers, or web links for publicly available datasets
- A description of any restrictions on data availability
- For clinical datasets or third party data, please ensure that the statement adheres to our policy

Data from the NYU Damodaran database is available here: https://pages.stern.nyu.edu/~adamodar/. Data from the Kroll Cost of Capital Navigator is proprietary and available only to subscribers who can obtain access here: https://www.kroll.com/en/tools-and-platforms/cost-of-capital. OECD data on US Treasury bond yields is available here: https://fred.stlouisfed.org/series/IRLTLT01USM156N. PwC data on corporate tax rates is available here: https://taxsummaries.pwc.com/interactive-map. GDP and GDP per capita data from the World Bank WDI database are available here: https://databank.worldbank.org/source/world-development-indicators.

All non-proprietary input data and derivatives used in this study (i.e., excluding Kroll-based data used only for robustness checks), including the main cost of capital estimates and the IMAGE output data, are available via Zenodo at https://doi.org/10.5281/zenodo.21922274. Source data are provided with this paper.

Research involving human participants, their data, or biological material

Policy information about studies with human participants or human data. See also policy information about sex, gender (identity/presentation), and sexual orientation and race, ethnicity and racism.

Reporting on sex and gender	N/A - No human research participants
Reporting on race, ethnicity, or other socially relevant groupings	N/A - No human research participants
Population characteristics	N/A - No human research participants
Recruitment	N/A - No human research participants
Ethics oversight	N/A - No human research participants

Note that full information on the approval of the study protocol must also be provided in the manuscript.

Field-specific reporting

Please select the one below that is the best fit for your research. If you are not sure, read the appropriate sections before making your selection.

☐ Life sciences ☐ Behavioural & social sciences ☒ Ecological, evolutionary & environmental sciences

For a reference copy of the document with all sections, see nature.com/documents/nr-reporting-summary-flat.pdf

Ecological, evolutionary & environmental sciences study design

All studies must disclose on these points even when the disclosure is negative.

Study description	The study uses the IMAGE integrated assessment model, which is described in detail in the Methods section. To estimate input parameters of interest, we estimate cost of capital by country, region and sector and conduct supplementary regression analysis.
Research sample	The regression analysis on tech-specific cost of capital estimates uses all available after-tax WACC estimates compiled by Steffen et al. (2025, Scientific Data).<br>The regression analysis on the statistical link between (log-transformed) GDP per capita and country risk premiums is based on a panel dataset constructed from the NYU Damodaran estimates for the rating-based country risk premium and World Bank estimates for GDP, GDP per capita, and population.
Sampling strategy	No sampling procedure was applied. All statistical calculations informing our IMAGE input parameters use all available observations.
Data collection	Data collected for the statistical calculations were directly downloaded from the listed database sources and, in the case of the CRP-GDPpc sample, merged at the country-year level.
Timing and spatial scale	IMAGE runs from 1970 through 2100 in yearly time steps; however, in line with the harmonized IAM reporting framework, we use output data in five-year (ten-year) intervals through (after) 2050 and, where necessary, interpolate linearly. IMAGE covers 26 aggregated regions.<br>The observations for the regression analysis are annual and at the country level, using all available data in the respective sources.
Data exclusions	No data were excluded from the analysis.
Reproducibility	N/A - No experiments conducted
Randomization	N/A - No experiments conducted
Blinding	N/A - No experiments conducted

Did the study involve field work? ☐ Yes ☒ No

Reporting for specific materials, systems and methods

We require information from authors about some types of materials, experimental systems and methods used in many studies. Here, indicate whether each material, system or method listed is relevant to your study. If you are not sure if a list item applies to your research, read the appropriate section before selecting a response.

Materials & experimental systems

n/a	Involved in the study
☒	☐ Antibodies
☒	☐ Eukaryotic cell lines
☒	☐ Palaeontology and archaeology
☒	☐ Animals and other organisms
☒	☐ Clinical data
☒	☐ Dual use research of concern
☒	☐ Plants

Methods

n/a	Involved in the study
☒	☐ ChIP-seq
☒	☐ Flow cytometry
☒	☐ MRI-based neuroimaging

Plants

Seed stocks	N/A - No plants used
Novel plant genotypes	N/A - No plants used
Authentication	N/A - No plants used
